# A Computational Study of a [2]Rotaxane Molecular Shuttle with All‐Atoms Molecular Dynamics and Density Functional Theory Simulations in Solution

**DOI:** 10.1002/cphc.202500660

**Published:** 2025-11-18

**Authors:** Costantino Zazza, Nico Sanna, Stefano Borocci, Felice Grandinetti

**Affiliations:** ^1^ Department for Innovation in Biological, Agro‐food and Forest systems Università della Tuscia (DIBAF) L.go dell’Università, s.n.c. 01100 Viterbo Italy; ^2^ CNR‐ISTP (Istituto per la Scienza e Tecnologia dei Plasmi) Via G. Amendola 122/D 70126 Bari Italy; ^3^ Istituto per i Sistemi Biologici del CNR (ISB) Sede di Roma–Meccanismi di Reazione c/o Dipartimento di Chimica Sapienza Università di Roma P.le A. Moro 5 001855 Rome Italy

**Keywords:** density functional theories, molecular shuttles, nanoscale devices, quantum theory of atoms in molecules

## Abstract

A rigid H‐shaped [2]rotaxane shuttle composed by a mechanically interlocked 24‐crown‐8(**24C8**) macrocycle on a thread containing two symmetrical benzimidazole (Bzi) stations bound with a central 2,2’‐bipyridyl (Bipy) core is addressed in CH_2_Cl_2_ solution with all‐atoms molecular dynamics simulations. The experimentally observed conformational preferences of the **24C8** ring quantitatively characterizing the free‐energy landscape driving its reversible translocation over the synthetic Stop‐[Bzi‐Bipy‐Bzi]‐Stop thread at room temperature have been reproduced. Also, this analysis to a translationally inactive form in N,N‐dimethylformamide (DMF) dilute solution following the coordination of PtCl_2_ to the Bipy chelate site is extended. In this respect, in the presence of PtCl_2_, the optimized geometry within the density functional theory (DFT) framework is fully characterized in terms of quantum theory of atoms in molecules (QTAIM) descriptors. Converged DFT wavefunctions in a continuum environment are analytically investigated by means of electron density *ρ(**r**)*, local electronic energy density, *H(**r**),* electron localization function (ELF), and delocalization index δ(X,Y) analysis. The derived picture highlights that the contextual presence of supramolecular contacts confining the **24C8** ring over its primary recognition site, and of a planar square (Bipy)‐N_2_‐Pt^(II)^Cl_2_ coordination environment parallel to the axle should actually be effective in suppressing the shutting movement as hypothesized via ^1^H‐nuclear magnetic resonance measurements.

## Graphical abstract

A H‐shaped [2]rotaxane is investigated in CH_2_Cl_2_ with all‐atoms molecular dynamics simulations combining quantum theory of atoms in molecules (QTAIM) descriptors with density functional theory (DFT) algorithms. Also, the nature of the chemical interactions modulating the formation of a square planar complex following the coordination of a PtCl_2_ moiety over a 2,2’‐bipyridyl chelate site in N,N‐dimethylformamide (DMF) is fully characterized.

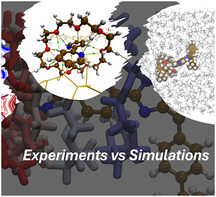

## 1 Introduction

Molecular rotaxanes represent a captivating class of mechanically interlocked molecules (MIMs), characterized by a dumbbell‐shaped component threaded through a macrocyclic ring, with bulky end groups—known as stoppers—preventing dissociation.^[^ ^1^, ^2^, ^3^ ^]^ Unlike covalently bonded structures, the subcomponents of rotaxanes are held together through mechanical rather than chemical bonds, allowing for controlled motion and conformational freedom at the molecular scale. This unique topology offers exciting opportunities to design systems with dynamic functionalities, setting rotaxanes apart from traditional molecules in both behavior and applications.^[^ ^4^, ^5^, ^6^, ^7^, ^8^, ^9^ ^]^ Since their first synthetic realization in the 1960s,^[^ ^10^ ^]^ the field of rotaxane chemistry has experienced remarkable growth. The development of efficient and selective synthetic methodologies—particularly template‐directed strategies such as metal‐ion coordination, hydrogen bonding, radical pairing, and hydrophobic interactions—has enabled the precise construction of complex rotaxane architectures with high yields and increasing functionalities.^[^ ^11^, ^12^ ^]^ These advances have transformed rotaxanes from synthetic curiosities into reliable platforms for probing fundamental questions in supramolecular chemistry. Rotaxanes have then become pivotal in the development of molecular machines, where the controlled movement of their components (such as shuttling or pirouetting of the macrocycle along the axle) can be modulated by external stimuli including light, pH, redox changes, or chemical fuels.^[^ ^13^, ^14^, ^15^, ^16^, ^17^, ^18^, ^19^, ^20^, ^21^, ^22^, ^23^ ^]^ Furthermore, the tunable host–guest interactions and spatially defined architecture of rotaxanes make them promising candidates for applications in catalysis, targeted drug delivery, stimuli‐responsive materials, and molecular electronics. As a result, a complete understanding of their structural dynamics, supramolecular interaction patterns, electronic transitions, and spectroscopic signatures under different environments is crucial for optimizing their performance.^[^ ^24^, ^25^ ^]^

Still remaining in this fascinating field, Loeb et al. recently proposed a synthetic interlocked H‐shaped [2]molecular rotaxane composed by a 24‐crown‐8 ether (**24C8**) macrocycle in noncovalent interaction with a molecular thread composed by a central 2,2’‐bipyridyl (Bipy) core and two symmetrical benzimidazole (Bzi) recognition sites.^[^ ^26^ ^]^ Based on transient ^1^H‐nuclear magnetic resonance (NMR) spectra, it was hypothesized that the central unit was capable of slowing down the **24C8** shuttling movement between the two Bzi stations as a result of the electronic repulsion involving Bipy N‐atoms and the crown ether O‐atoms.^[^ ^27^ ^]^ The addition of Zn(II) ions in N,N‐dimethylformamide (DMF), after complexation with the nitrogen atoms of the Bipy chelate, still promoted the shuttling of the **24C8** via the formation of an octahedral intermediate also involving solvent molecules and the macrocycle itself.^[^ ^26^, ^28^ ^]^ On the contrary, the coordination of a PtCl_2_ moiety to the same chelating unit resulted in a complex featuring a square planar geometry, which generated an insurmountable steric barrier to shuttling motion.^[^ ^26^ ^]^ Herein, we addressed for the first time such an H‐shaped [2]rotaxane shuttle in CH_2_Cl_2_ solution at room temperature conditions with all‐atom molecular dynamics (MD) simulations^[^ ^29^ ^]^ reproducing the experimentally observed conformational preferences. The derived analysis of the supramolecular contacts between the **24C8** and the thread via the quantum theory of atoms in molecules (QTAIM)^[^ ^30^, ^31^, ^32^ ^]^ at density functional theory (DFT)^[^ ^33^ ^]^ cost has revealed the presence of a middle point transient species potentially capable of modulating the observed shuttling process. Finally, we also characterize the coordination of a PtCl_2_ moiety to the Bipy unit in a square planar geometry in conjunction with converged DFT electron density *ρ(**r**)*, local electronic energy density *H(**r**)*, and Bader's topology analysis descriptors.^[^ ^28^, ^34^, ^35^, ^36^, ^37^, ^38^ ^]^

## 2 Computational Section

Solvation and thermal effects modulating the conformational shaping in the investigated H‐shaped [2]rotaxane molecular shuttle are addressed by using an all‐atom classical MD technique.^[^ ^29^ ^]^ We considered as reference condition the X‐ray structure showing the **24C8** cycle encircling one of two Bzi stations.^[^ ^26^ ^]^ Such a structure was minimized using the hybrid Becke 3‐parameters Lee–Yang–Parr (B3LYP)^[^ ^39^, ^40^, ^41^ ^]^ functional in conjunction with the 6–31 G** basis set.^[^ ^42^ ^]^ The Grimme's dispersion term with the original D3 damping function was also used to improve the description of van der Waals (vdW) contacts.^[^ ^43^ ^]^ Moreover, we modeled medium and polarizable CH_2_Cl_2_ solvation effects via conductor‐like polarizable continuum (C‐PCM) method.^[^ ^44^ ^]^ Afterward, the derived geometry was used to extract bonds, angles, dihedrals, and Lennard‐Jones OPLS‐AA parameters from the LigParGen web‐based service.^[^ ^45^, ^46^, ^47^ ^]^ Atomic point charges were estimated using the molecular electrostatic potential (MEP) method of Merz‐Kollman as implemented in the Gaussian 16 code;^[^ ^48^ ^]^ all bonds involving hydrogen atoms were constrained using the LINCS algorithm.^[^ ^49^ ^]^ For the organic solvent (i.e., CH_2_Cl_2_), we used the parameters proposed by Van der Spoel and coworkers.^[^ ^50^ ^]^ MD simulations were performed using GROMACS Software (release 2022.6)^[^ ^51^ ^]^ considering the synthetic rotaxane at the centre of a cubic box of edge length of 48 Å. Afterward, a 200 ns classical MD simulation (*T = *298 K, *p = *1 bar) was carried out following an equilibration trajectory of 1 ns. Also, we investigated the free‐energy landscape driving the reversible translocation of the **24C8** ring over the Stop‐[Bzi‐Bipy‐Bzi]‐Stop thread and characterized via ^1^H‐NMR signals.^[^ ^26^ ^]^ We selected, as internal coordinate, the distance between the center‐of‐mass (COM) of the **24C8** ring and that of the opposite free Bzi group.^[^ ^52^, ^53^ ^]^

In addition, we focused our attention on the characterization of a proposed translationally inactive form of such a [2]rotaxane in DMF following the coordination of PtCl_2_ to the Bipy chelate site. The Stop‐[Bzi(**24C8**)‐Bipy(PtCl_2_)‐Bzi]‐Stop system was optimized at C‐PCM(DMF)/B3LYP(D3) level. The H, C, O, and N atoms were treated with Dunning's correlation consistent‐polarized cc‐pVTZ basis sets,^[^ ^42^ ^]^ while the Pt atom with its effective core pseudopotential (PP) form (cc‐pVTZ‐PP).^[^ ^54^ ^]^ This system was then optimized and the converged electronic wavefunction analyzed in terms of the electron density *ρ*(***r***), the electronic energy density *H*(***r***) and its kinetic and potential components *G*(***r***) and *V*(***r***), respectively. The *ρ(**r**)* is defined by the equation
(1)

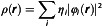

where $\eta_{i}$ is the occupation number of the natural orbital $\varphi_{i}$, in turn, expanded as a linear combination of the atomic basis functions.^[^ ^33^ ^]^ The *H(**r**)* is the sum of the kinetic energy density *G(**r**)* and the potential energy density *V(**r**)*.^[^ ^30^, ^31^, ^32^ ^]^

(2) H(r)=G(r)+  V(r)

The presently‐employed definition^[^ ^30^, ^31^, ^32^, ^55^ ^]^ of the *G(**r**)* is given by the equation
(3)

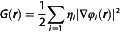

with the sum running over all the occupied natural orbitals $\varphi_{i}$ of occupation numbers $\eta_{i}$. The potential energy density *V(**r**)* is evaluated from the local form of the virial theorem

(4) $$V\left(r\right)=\frac{1}{4}\nabla^{2}\rho \left(r\right)-2G\left(r\right)$$

The aforementioned indicators were estimated using the Multiwfn program (version 3.8.dev).^[^ ^56^ ^]^ A visual study of the underlying supramolecular interactions is also accomplished by using a variant of the independent gradient model (IGM)^[^ ^57^ ^]^ which substitutes the free‐state atomic densities (density under promolecular approximation) with those directly derived by a Hirshfeld partitioning (IGMH)^[^ ^58^, ^59^ ^]^ of actual molecular electron density reflecting a more rigorous physical meaning of the electronic structure of the system investigated. Furthermore, the coordination of PtCl_2_ to the Bipy site in a planar square arrangement was investigated by plotting the contour lines associated with the derived *H(**r**)* values. Such a function—as successfully observed in the presence of an octahedral intermediate following the coordination of Zn(II) cation—partitions the atomic space into inner regions featuring negative values, indicated as *H*_−_ *(**r**)*, and outer regions of positive values, indicated as *H* ^*+*^ *(**r**)*.^[^ ^28^ ^]^ When two atoms form a chemical bond, these regions combine in ways that signal the nature of the interaction.^[^ ^28^, ^34^, ^35^, ^36^, ^37^, ^38^ ^]^ This provides a simple and intuitive picture to rationalize the nature of the chemical interactions driving the complexation pathway. Finally, this analysis was also supported by the ELF plot, which, by providing a measure of the localization of electrons in any sub‐part of a given total electron density (i.e., *ρ(**r**)*), allows to identify the local regions of space where it is possible to find electron pairs.^[^ ^60^, ^61^ ^]^

## 3 Results and Discussion

### 3.1 The [2]Rotaxane in CH_2_Cl_2_ Dilute Solution at 298K

**Figure 1:**
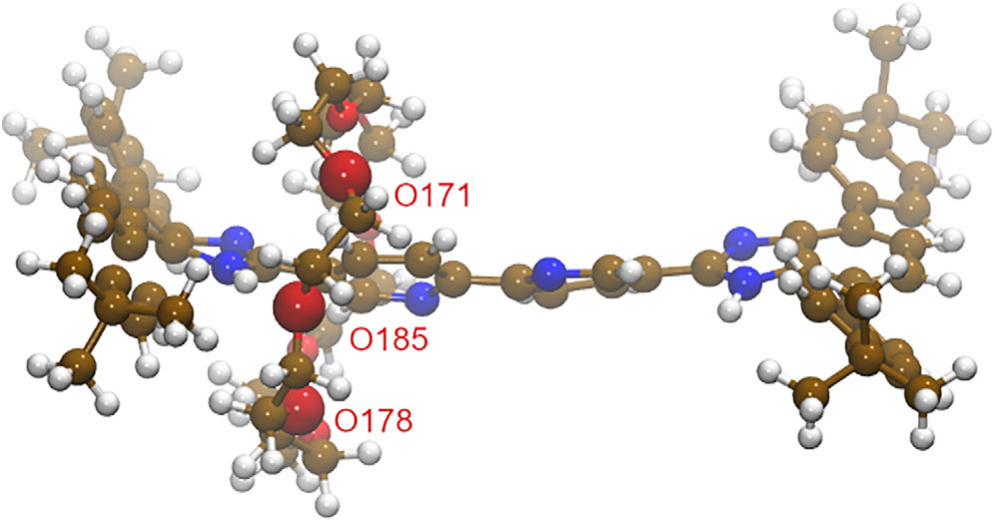
The optimized structure of the Stop‐[Bzi‐Bipy‐Bzi]‐Stop molecular thread hosting the **24C8** ether ring at C‐PCM(CH_2_Cl_2_)‐B3LYP(D3)/cc‐pVTZ level. The proximal oxygen atoms in electrostatic interaction with the N–H chemical unit of the Bzi station are highlighted following the indexing used during the classical MD sampling (see Figure 2).

**Figure 2:**
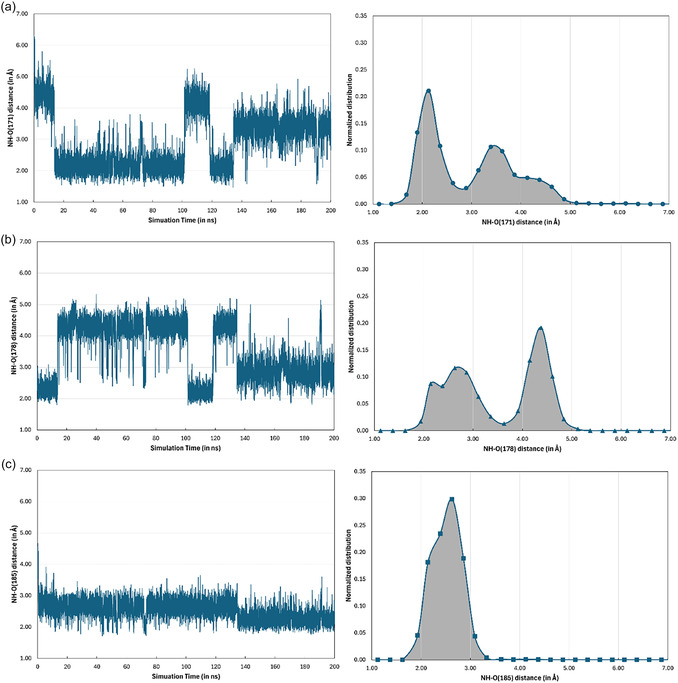
a) (Bzi)N–H—O_171_(**24C8**) interatomic distance trajectory as extracted from the 200 ns MD at 298 K in CH_2_Cl_2_ with the associated normalized distribution; b) (Bzi)N–H—O_178_(**24C8**) interatomic distance trajectory with the associated normalized distribution; c) (Bzi)N–H—O_185_(**24C8**) interatomic distance trajectory with the associated normalized distribution.

We first investigated the conformational shaping adopted by the synthetic Stop‐[Bzi(**24C8**)‐Bipy‐Bzi]‐Stop [2]rotaxane in CH_2_Cl_2_ solution at finite‐room temperature. At first, our MD trajectory substantially shows that the Stop‐[Bzi‐Bipy‐Bzi]‐Stop molecular thread results are rather rigid during the sampling and contextually, at least within the simulated time domain (200 ns), the encircling **24C8** macrocycle remains in electrostatic interactions with the Bzi station selected for hosting the cycle on the basis of the initial coordinates displayed in **Figure** **1**. As a matter of fact, looking at the (Bzi)N‐H—O171/178/185(**24C8**) interatomic distances as extracted from the 200 ns MD at 298 K in CH_2_Cl_2_, it is easy to realize the H‐shaped network connecting—via supramolecular interactions—the **24C8** and Bzi moieties. A triad of oxygen atoms from the ether group of the macrocycle effectively participates in the formation of hydrogen bonds with the N–H group of the Bzi station. More in details, looking at the derived noncovalent interaction patterns depicted in **Figure** **2**a,b, an electrostatic mutual switch between the extremal O171 and O178 is observed with respect to the secondary N–H amine constituting the Bzi station; this is also witnessed by the presence of a sort of trimodal distribution modulating the (Bzi)N–H—O171(**24C8**) and (Bzi)N–H—O178(**24C8**) intermoiety distances along the classical sampling: i) the normalized distribution of the N–H—O171 distance features a well‐defined peak a shorter distances with a maximum lying at 2.13 Å; subsequently the distribution shows a second peak at 3.39 Å with a shoulder within 4.13 and 5.00 Å. ii) The N–H—O178 values at shorter distances result characterized by the convolution of two resolved peaks at 2.14 and 2.63 Å, with the latter showing a slightly larger population; such a distribution also reveals the presence of a subset of MD snapshots showing N–H—O178 distances larger than 3.70 Å and peaked at 4.38 Å. iii) In addition, the central oxygen ether atom (i.e., O185 in Figure 1) results systematically in electrostatic interaction via H‐bond with the H–N(Bzi) moiety. From a statistical point of view, the (Bzi)N–H—O185(**24C8**) interatomic distance trajectory span values in the range between 1.92 and 3.09 Å with a maximum peaked at 2.63 Å (the most recurring value estimated from the normalized distribution displayed in Figure 2c). It is therefore evident that a cooperative network of electrostatic contacts between Bzi and **24C8** units plays a key role in maintaining the macrocycle in proximity of the associated station, as already revealed via ^1^H‐NMR experiments in the same solvent.^[^ ^26^ ^]^

**Figure 3:**
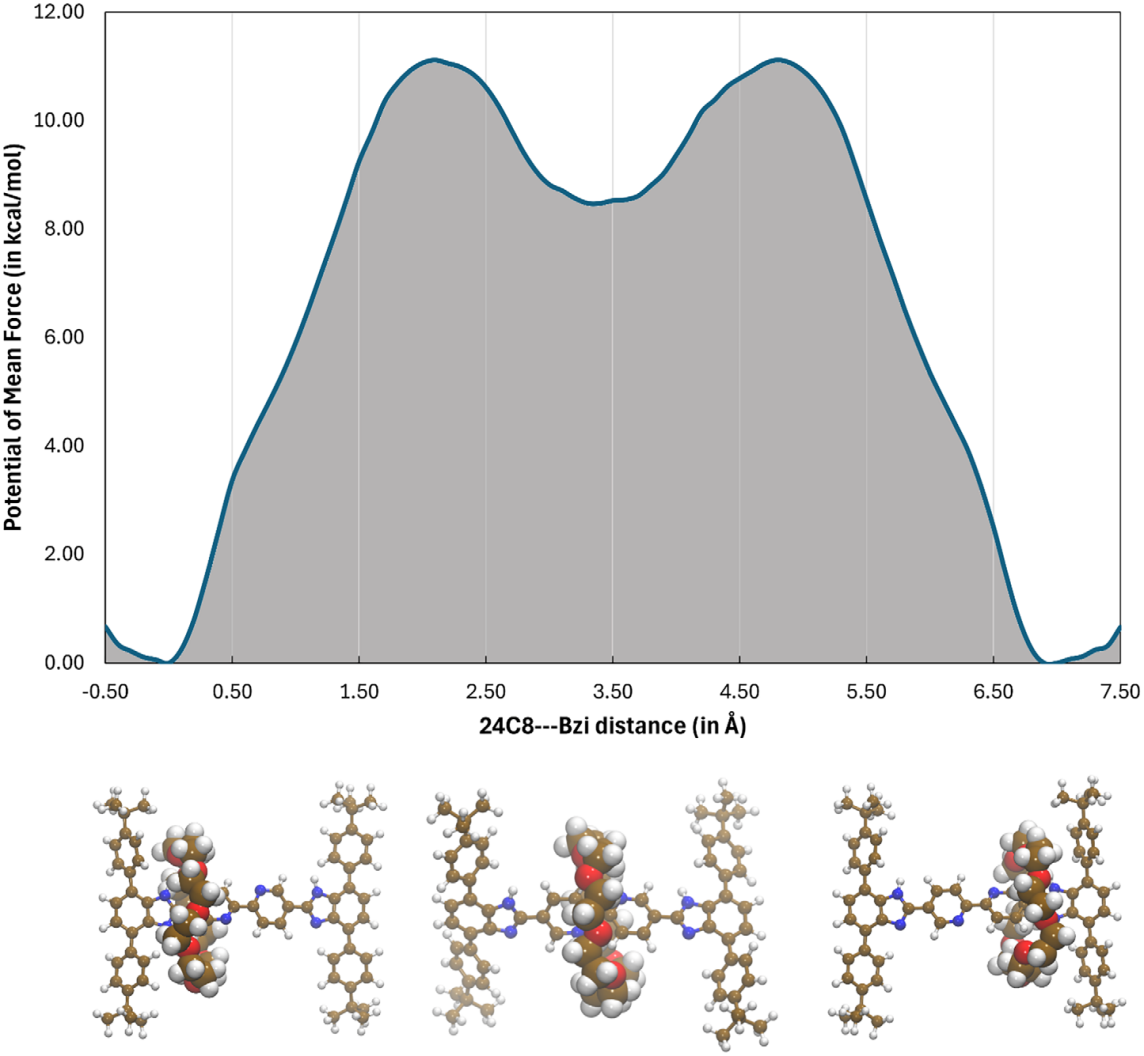
Free‐energy profile (in kcal mol^−1^) of the Stop‐[Bzi(24C8)‐Bipy(PtCl_2_)‐Bzi]‐Stop [2]rotaxane along the Bzi→Bzi shuttling coordinate (see the text) in CH_2_Cl_2_ dilute solution as estimated via all‐atoms classical MD simulations at finite room temperature conditions; representative structures of the two end minima and of the transient mechanical species—lying at the mid‐point between the two Bzi recognition sites—are also reported in the lower panel.

Afterward, always inspired by experiments, we focused our effort on modeling associated with the characterization of the symmetrical translational movement of the macrocycle between the two Bzi recognition sites of a predominantly electrostatic nature.^[^ ^26^, ^27^, ^28^ ^]^ For doing this, as reported in Section 2, we applied the umbrella sampling technique as proposed in the Gromacs Software^[^ ^51^ ^]^ to compute the potential of mean force (PMF) along such a shuttling dynamical process with the popular weighted histogram analysis method (WHAM).^[^ ^52^ ^]^ We generated a series of configurations along the shuttling coordinate that, in this specific case, is represented by the center‐of‐mass distance between the **24C8** cycle and the free Bzi station localized over the right side of the Stop‐[Bzi‐Bipy‐Bzi]‐Stop molecular thread (see Figure 1). A harmonic restraint potential of 30 kJ mol^−1^ Å^−2^ was applied to pull the macrocycle toward the other symmetric Bzi recognition site within a region of ≈8 Å and composed of 16 evenly spaced umbrella sampling windows. Afterward, a classical MD simulation (at 298 K and 1 bar) of 20 ns was carried out selecting a representative structure of the investigated [2]rotaxane for each interval. Please note that the same procedure was already applied by one of us (CZ) to investigate a light‐driven rotor featuring an acid–base‐triggered self‐complexing lock in CH_2_Cl_2_ solution.^[^ ^23^ ^]^ The free‐energy profile, in solution at 298 K, along the Bzi[**24C8**]→Bzi motion reported in **Figure** **3** shows, as it would have been expected, a symmetric shape with a significant penalty accompanying the translocation of the macrocycle. As a matter of fact, the derived free‐energy profile evidences the presence of a thermodynamics barrier of ≈11.2 ± 0.2_7_ kcal mol^−1^ at 2.2 and 4.8 Å, with statistical errors estimated with a bootstrap analysis assigning random weights to the histograms along the imposed shuttling coordinate, arising from a subtle balance between noncovalent energetic and entropic contributions concerning both the investigated [2]rotaxane and the encircling solvent.^[^ ^50^ ^]^ In particular, the breaking of the intermoiety (Bzi/**24C8**) H‐bonding network, as previously described in Figure 2, and the solvent stabilization of the released Bzi station do play a crucial role in modulating the observed barrier. Always remaining in this context, variable temperature ^1^H‐NMR spectra had been used for estimating a shuttling kinetic of 61.2 s^−1^ associated with an activation free energy barrier (ΔG^‡^) of 15.1 kcal mol^−1^; the observed trend results were then well reproduced by numerical simulations. In this respect, it is worth noting that previous quantum mechanics computations in DMF solvent had overestimated the shuttling potential energy barrier, providing a value of ≈24 kcal mol^−1^.^[^ ^28^ ^]^ Furthermore, the profile shown in Figure 3 reveals another interesting aspect that needs to be discussed. At the middle point with a shuttling coordinate of ≈3.4 Å, we can appreciate a local minimum basin with a free energy increase of 8.6 ± 0.1_6_ kcal mol^−1^ with respect to the reference condition in which the cycle resides at one of the Bzi recognition sites.

**Figure 4:**
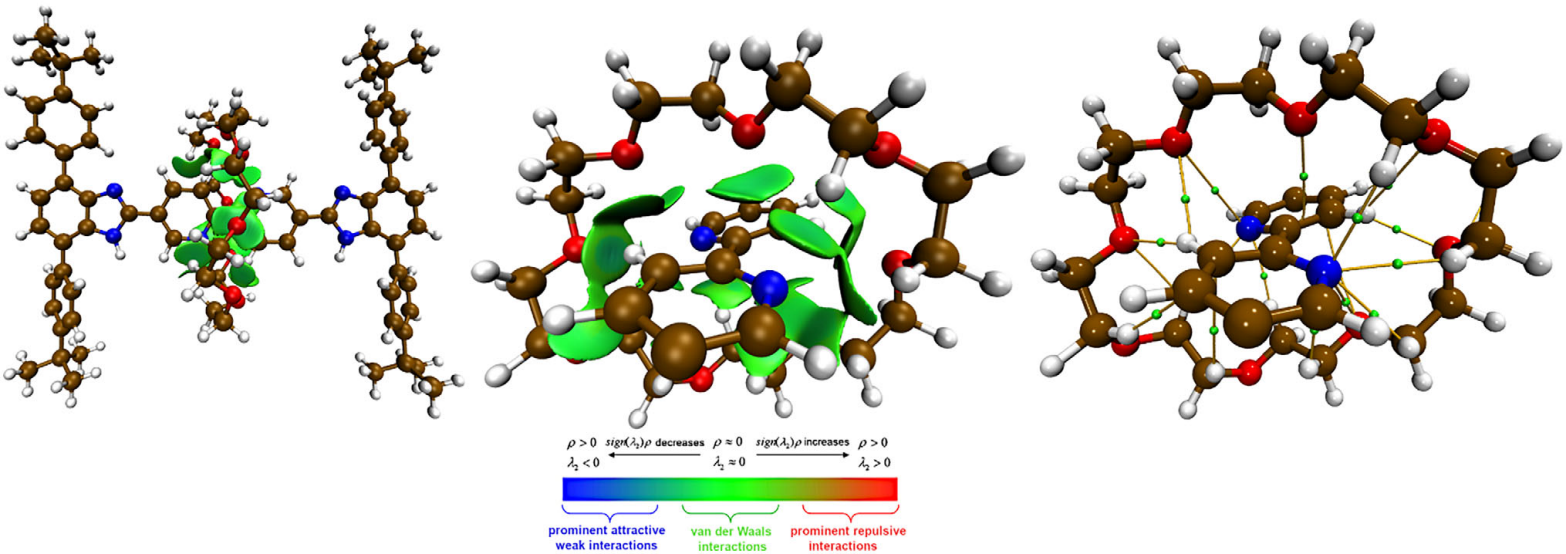
From left to right: i) *sign*(*λ*_2_)*ρ* colored isosurfaces of *δ*g^inter^ *= *0.005 a.u. –at C‐PCM(CH_2_Cl_2_)B3LYP(D3)/cc‐pVTZ level– corresponding to IGMH analysis carried out over the investigated [2]rotaxane in its relaxed structure at the middle point between the two Bzi chemical units; ii) local view of the interacting Bipy‐**24C8** region; and iii) a view of the BPs modulating Bipy‐**24C8** interactions highlighted using green points analyzed in Table 1.

A more punctual analysis of the noncovalent interaction patterns may be derived looking at the data collected in **Table** **1**. The interface region between the rigid molecular thread and the **24C8** cycle actually features numerous *ρ(**r***_***cp***_ *)* bond critical points (BCPs) of the type (+3,−1).^[^ ^62^, ^63^ ^]^ More specifically, the indipendent gradient model with Hirshfeld partitioning (IGMH) isosurfaces^[^ ^58^, ^59^ ^]^ with a NCIs coloring method ^[^ ^55^ ^]^ of *δ*g^inter^ and the underlying BCPs connecting the Bipy‐**24C8** interacting couple are highlighted in **Figure** **4** and fully characterized in Table 1 in terms of QTAIM‐derived indicators: i) the Laplacian of the density [$\nabla^{2}$ *ρ(**r**)*] as derived from the trace of the Hessian matrix of the *ρ(**r**)*; this allows an analysis of the curvatures of the *ρ(**r**)* at any estimated BCPs along the principal axes. ii) The values of the *G(**r**)* and *V(* **r**) to analyze the kinetic and potential energy densities; this for evaluating if interactions are controlled by a local reduction of the potential energy or by a local excess in the kinetic energy [*G(**r**) *> 0 and *V(**r**) *< 0, see Equation (3) and (4)]. iii) The total energy density *H(* **r**), see Equation (2), which is negative for interactions featuring a significant sharing of electrons, so to detect the covalent or partially covalent character of the estimated interaction patterns. Also, it should be mentioned that, at the estimated middle point, no BCPs are observed between the **24C8** macrocycle and the terminal units of Bzi, as clearly displayed in Figure 4. QTAIM and IGMH analyses reveal that noncovalent forces act as a sort of glue stabilizing the cycle in proximity to the central Bipy unit. For most estimated BCPs involving different functional groups, the *ρ(**r**)* is within 0.012 *e*·*a*_*0*_^−3^, the $\nabla^{2}$ *ρ(**r**)* and the *H*(***r***) are invariably positive, and the *–G(**r**)/V(**r**)* ratio results greater than one. Six bond paths (BPs)^[^ ^62^, ^63^ ^]^ of the type N–H–C(*sp* ^2^) are established by the **24C8** cycle (Mac) with the encircling Bipy chemical unit featuring interatomic distances between 2.44 and 2.75 Å.

**Table 1:** Electron density *ρ(**r**)* (*e*·*a*_*0*_^−3^), Laplacian of electron density ∇^2^ *ρ(**r**)* (*e*·*a*_*0*_^−5^), electron kinetic energy density *G(**r**)* (hartree·*a*_*0*_^−3^), electron potential energy density *V(**r**)* (hartree·*a*_*0*_^−3^), and electron energy density *H(**r**)* (hartree·*a*_*0*_^−3^) for BCPs on selected bonds of the optimized [2]molecular rotaxane with the macrocycle hosting the Bipy station at the middle point along the shuttling coordinate at C‐PCM(CH_2_Cl_2_)/B3LYP(D3)/cc‐pVTZ level.

BCP	Moieties	Distance [Å]	*ρ*(***r***)	∇^2^ *ρ*(***r***)	*G*(***r***)	*V*(***r***)	*−G*(***r***)/ *V*(***r***)	*H*(**r**)
N–H–C(*sp* ^2^)	Bipy‐Mac	2.44	0.012	0.033	0.008	−0.007	1.103	0.001
N–H–C(*sp* ^2^)	Bipy‐Mac	2.45	0.011	0.033	0.008	−0.007	1.090	0.001
N–H–C(*sp* ^2^)	Bipy‐Mac	2.61	0.009	0.027	0.006	−0.006	1.102	0.001
N–H–C(*sp* ^2^)	Bipy‐Mac	2.65	0.008	0.028	0.006	−0.005	1.163	0.001
N–H–C(*sp* ^2^)	Bipy‐Mac	2.69	0.008	0.021	0.005	−0.004	1.090	0.000
N–H–C(*sp* ^2^)	Bipy‐Mac	2.75	0.007	0.023	0.005	−0.004	1.210	0.001
C(sp^2^)–H–O	Bipy‐Mac	2.18	0.018	0.049	0.013	−0.013	0.979	0.000
C(sp^2^)–H–O	Bipy‐Mac	2.23	0.016	0.047	0.012	−0.012	0.990	0.000
C(sp^2^)–H–O	Bipy‐Mac	2.51	0.010	0.035	0.008	−0.007	1.138	0.001
C(*sp* ^2^)–H–O	Bipy‐Mac	2.53	0.009	0.029	0.007	−0.007	1.056	0.000
C(sp^2^)–H–O	Bipy‐Mac	3.01	0.010	0.035	0.007	−0.006	1.271	0.002
C(sp^2^)–H–O	Bipy‐Mac	3.21	0.006	0.020	0.004	−0.003	1.313	0.001
N–O	Bipy‐Mac	3.15	0.007	0.025	0.006	−0.005	1.121	0.001
N–O	Bipy‐Mac	3.19	0.006	0.021	0.005	−0.004	1.182	0.001
HC(*sp* ^3^)–H–C(sp^2^)	Bipy‐Mac	2.71	0.007	0.072	0.005	−0.004	1.361	0.001
HC(*sp* ^3^)‐H–C(*sp* ^2^)	Bipy‐Mac	3.15	0.004	0.011	0.002	−0.002	1.367	0.001

The remaining BPs are seen to involve C(*sp* ^2^)–H–O, N–O and HC(*sp* ^3^)–H–C(*sp* ^2^) interacting moieties. In the present context, we are actually referring to weak van der Waals (vdW) interactions of different nature; the only exception is a shorter pair of C(*sp* ^2^)–H–O contacts, with distances of ≈2.2 Å, featuring a negative value of the associated *H*(***r***) at the BCP. These contacts may be viewed as the strongest NCIs, as evidenced by the highest values of the estimated *ρ(**r**)* at the CP (0.018 and 0.016 *e*·*a*_*0*_^−3^, see data collected in Table 1]. Besides, for the sake of clarity, the BCPs connecting the Bipy‐Mac couple are highlighted in Figure 4. The herein proposed analysis then should plausibly highlight the existence of a rather complex supramolecular network in CH_2_Cl_2_ dilute solution allowing, although temporary at 298 K due to a free energy barrier of the same order of magnitude of the thermal fluctuations (see Figure 3), the guesting of the central Bipy unit via the **24C8** ether ring along the Bzi‐Bipy‐Bzi thread. Furthermore, this result is consistent with what has been observed,^[^ ^26^, ^27^ ^]^ with regard to the slowing down of the cycle translocation mechanism in the presence of the Bipy station (61.2 s^−1^ versus 6.2 × 10^3^ s^−1^); this should therefore be due to a dual effect: i) the “Pauli” electronic repulsion between the lone pairs localized over the N‐atoms of the Bipy unit and those of the crown ether O‐atoms (corresponding with the symmetrical barrier of ≈11.2 kcal mol^−1^ shown in Figure 3); and ii) the presence of a transient supramolecular aggregate between Bipy(guest)‐**24C8**(host) species in the middle between the two preferred recognition sites.

### 3.2 Theoretical Characterization of the [2]Rotaxane in the Presence of Pt(II)

Exchange spectroscopy spectrum unequivocally reported that the addition of [Zn(NTf_2_)_2_] in the presence of the [2]rotaxane in DMF solution preserves the translocation of the **24C8** macrocycle, although with a slower shuttling rate of 2.2 s^−1^ than that measured only considering the free rotaxane form (i.e., 61.2 s^−1^).^[^ ^26^ ^]^ This trend is attributed to the formation of an octahedral intermediate in which the Zn(II) cation, after attachment to the nitrogen atoms of the Bipy chelate, can bind easily in a reversible way, the crown ether during the translocation towards the two end‐points (i.e., Bzi units). In this respect, QTAIM‐based descriptors derived from converged electronic wavefunctions at conductor‐like polarizable continuum (C‐PCM)/DFT level provided a self‐consistent theoretical picture supporting the prediction of a ligand exchange mechanism in such a [2]rotaxane following the addition of Zn(II) ions.^[^ ^28^ ^]^ In this further investigation, we focused our attention on another intriguing result, namely the absence of a translocation mechanism downstream of the addition of PtCl_2_. This was qualitatively explained in terms of an insurmountable steric barrier to translation associated with the coordination of a PtCl_2_ moiety to the Bipy unit in a square planar conformation.^[^ ^26^ ^]^ Consequently, based on this experimental evidence, we have then fully analyzed the relaxed geometry of a Stop‐[Bzi(**24C8**)‐Bipy(PtCl_2_)‐Bzi]‐Stop compound by means of QTAIM‐based descriptors with the target of addressing the nature of the chemical interactions characterizing such a complexation in DMF solution media.

**Figure 5:**
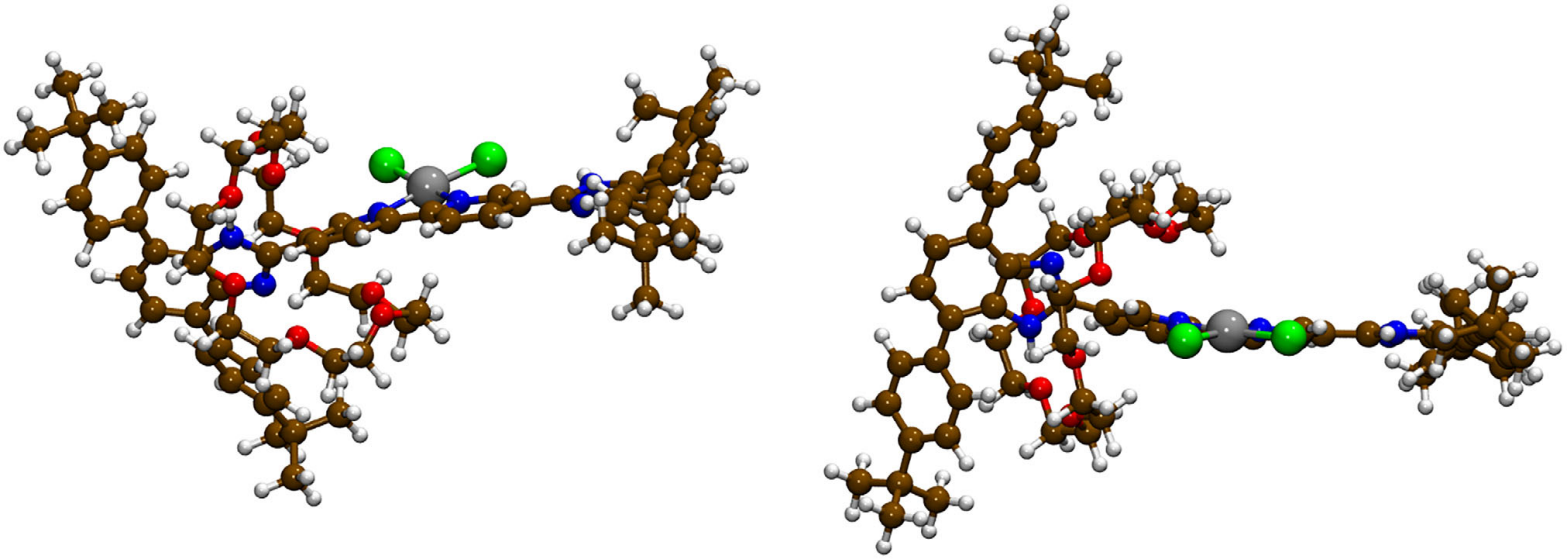
Two different views showing the optimized molecular structure of the investigated [2]molecular shuttle with PtCl_2_ moiety at C‐PCM(DMF)/B3LYP(D3)/cc‐pVTZ level of computation; chlorine atoms are reported in green while the platinum atom in silver.

The C‐PCM(DMF)/B3LYP(D3)/cc‐pVTZ optimized geometry of the Stop‐[Bzi(**24C8**)‐Bipy(PtCl_2_)‐Bzi]‐Stop complex is displayed in **Figure** **5** **,** while the QTAIM‐based indicators, which analyze the electron density distribution in conjunction with Bader's topological parameters^[^ ^30^, ^31^, ^32^ ^]^ to understand the underlying bonding characteristics, are explicitly collected in **Table** **2**. At first, the *H*(***r***) collected in this table reports negative values for the (+3,‐1) BCPs connecting the Pt(II) cation with the Bipy chelating unit in DMF solvent media [*H*(***r***) = −0.042 and −0.041 hartree·*a*_*0*_^−3^, distance ≈2.04 Å]; the associated *ρ(**r**)* is estimated—at the BCPs—at almost 0.12 *e*·*a*_*0*_^−3^. These data reveal that the most prominent chemical interactions showing a covalent character are those connecting the Pt(II) species with the N atoms of the central unit in its *cis* conformation. In this respect, we would like to point out that a similar trend was observed in the case of the Zn(II) cation (always in DMF solvent) with the estimated *H*(***r***) values at the BCP[Zn^(II)^—N(Bipy)] equal to −0.012 and −0.011 hartree·*a*_*0*_^−3^.^[^ ^28^ ^]^ Yet, the Pt(II)–Cl_2_ interaction patterns showing a distance of 2.34 and 2.36 Å still suggest clear evidence for a covalence character, albeit of a lesser extent since *H*(***r***_***cp***_) takes values at around −0.02 hartree·*a*_*0*_^−3^, *ρ(**r***_***cp***_ *) *≈ 0.09 *e*·*a*_*0*_^−3^. In addition, the delocalization index, *δ*(X,Y), taking into account the aforementioned contacts within the proposed QTAIM picture at C‐PCM/DFT cost, are also computed. This basically because this indicator provides a direct measure of the number of electrons that are exchanged between X and Y atomic species, thus giving, in essence, the fractional number of electron pairs shared by two interacting atoms.^[^ ^64^, ^65^, ^66^ ^]^ In turn, based on the combined use of *δ*(X,Y) and *ρ(**r***_***cp***_ *),* the Pt^(II)^—N and Pt^(II)^—Cl bonds shown in Figure 5 can be assigned as covalent, reflecting *δ*(X,Y) values ≥ 0.8 *e* (see Table 2).^[^ ^37^, ^67^ ^]^ As a final result, the present QTAIM analysis of the C‐PCM(DMF)/B3LYP(D3)/cc‐pVTZ electronic wavefunction demonstrates, in a self‐consistent manner, that a PtCl_2_ chemical unit can firmly bind to the central Bipy chelating site, forming a planar square complex as hypothesized experimentally by Loeb and coworkers.^[^ ^26^ ^]^ The formation in silico environment of such a stable Stop‐[Bzi(**24C8**)‐Bipy(PtCl_2_)‐Bzi]‐Stop compound (see Figure 5) is supposed to be crucial for effectively blocking the translational motion of the **24C8** macrocycle in DMF solvent.^[^ ^26^, ^68^ ^]^

**Table 2:** Electron density *ρ(**r**)* (*e*·*a*_*0*_^−3^), Laplacian of electron density ∇^2^ *ρ(**r**)* (*e*·*a*_*0*_^−5^), electron kinetic energy density *G(**r**)* (hartree·*a*_*0*_^−3^), electron potential energy density *V(**r**)* (hartree·*a*_*0*_^−3^), electron energy density *H(**r**)* (hartree·*a*_*0*_^−3^) and QTAIM delocalization index δ(X,Y) (*e*) for BCPs on selected bonds of the optimized [2]molecular rotaxane with the PtCl_2_ moiety at C‐PCM(DMF)/B3LYP(D3)/cc‐pVTZ level. Data refer to geometrical structure shown in Figure 5.

BCP	Distance [Å]	*ρ*(***r***)	∇^2^ *ρ*(***r***)	*G*(***r***)	*V*(***r***)	*−G*(***r***)/ *V*(***r***)	*H*(**r**)	*δ*(X,Y)
Pt(II)–N	2.04	0.123	0.414	0.143	−0.186	0.772	−0.042	0.806
Pt(II)–N	2.04	0.121	0.428	0.145	−0.186	0.780	−0.041	0.801
Pt(II)–Cl	2.34	0.090	0.213	0.078	−0.104	0.751	−0.026	0.916
Pt(II)–Cl	2.36	0.085	0.214	0.075	−0.098	0.769	−0.023	0.884

**Figure 6:**
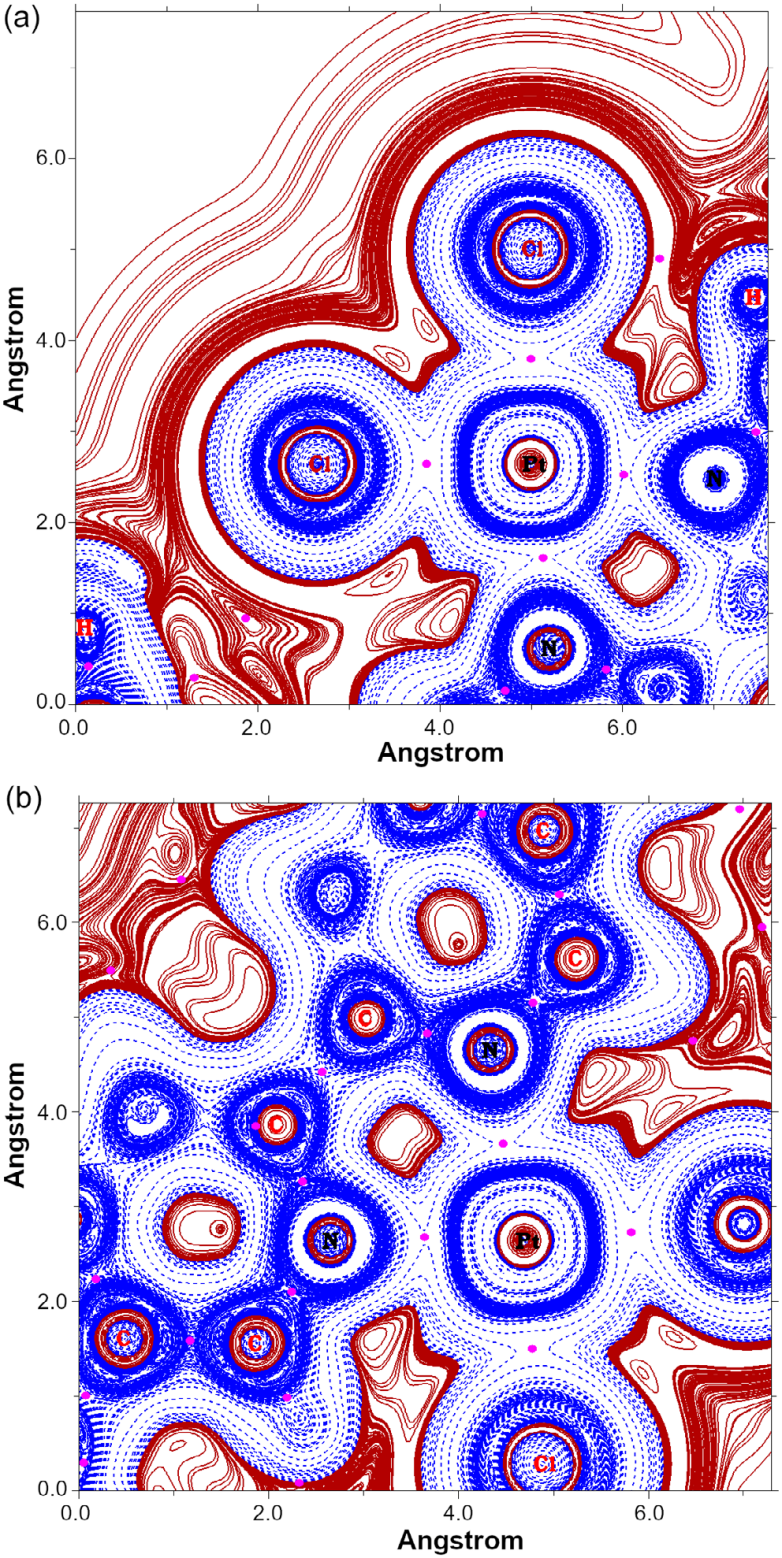
a) 2D plot (in hartree·*a*_*0*_^−3^) of the C‐PCM‐B3LYP(D3)/cc‐pVTZ *H(**r**)* in the σ plane defined by the position of Cl–Pt^2+^–Cl atoms in the optimized geometry of the Stop‐[Bzi(24C8)‐Bipy(PtCl_2_)‐Bzi]‐Stop complex. Solid/brown and dashed/blue lines correspond to positive and negative values of the *H(**r**)* function. The dots in magenta indicate the CPs (+3,‐1) from the topological analysis of the *H(**r**)*; and b) 2D plot of the *H(**r**)* function over a plane defined by N(Bzi)‐Pt^2+^‐N(Bzi) atoms.

**Figure 7:**
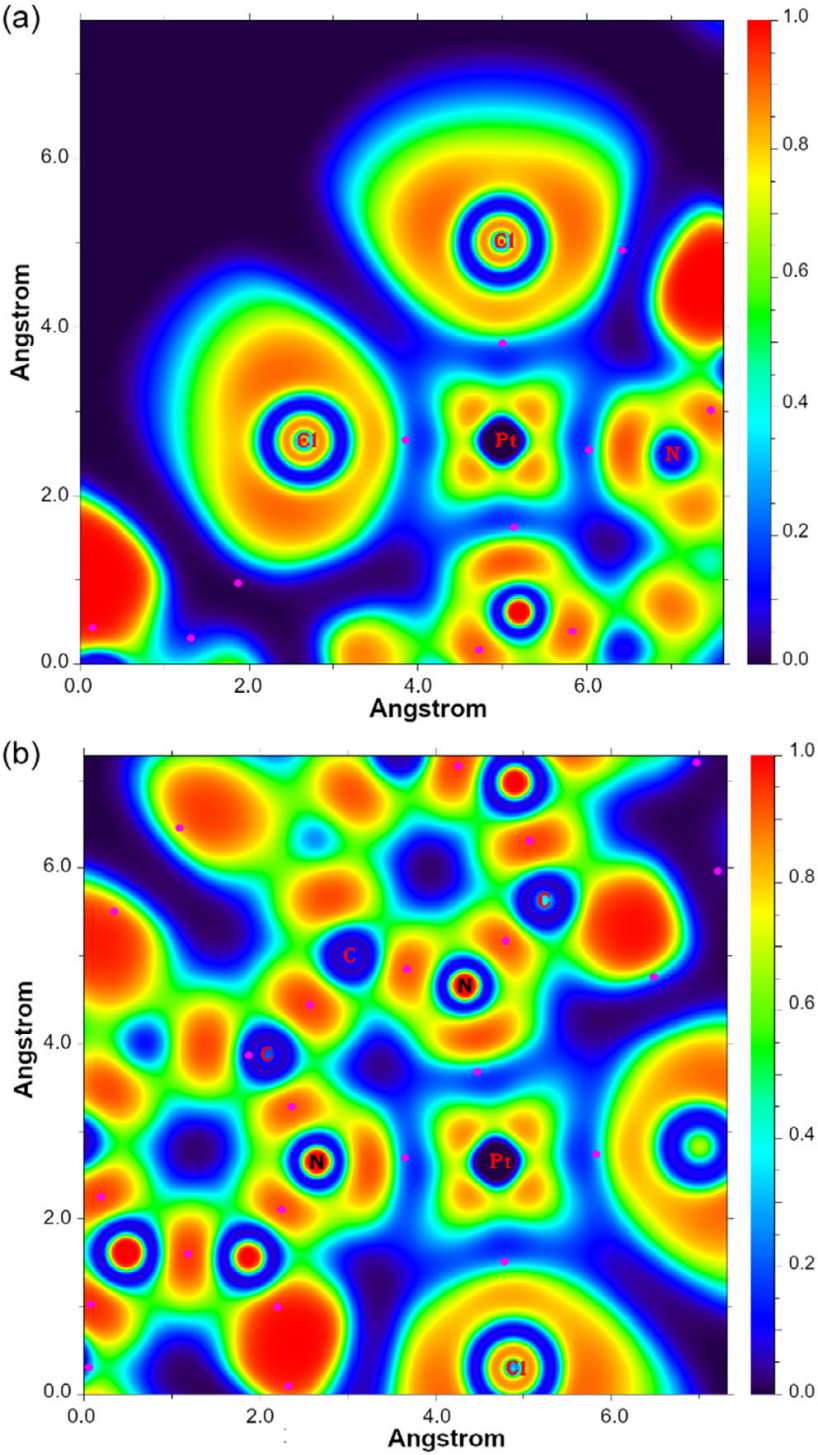
a) 2D plot of the C‐PCM‐B3LYP(D3)/cc‐pVTZ ELF in the σ plane defined by the position of Cl–Pt^2+^–Cl atoms in the optimized geometry of the Stop‐[Bzi(24C8)‐Bipy(PtCl_2_)‐Bzi]‐Stop complex; the color bar is also shown for ELF values ranging from 0 to 1. The dots in magenta indicate the CPs (+3,−1) arising from the topological analysis of the *H(**r**),* see also Figure 4; and b) 2D plot of the ELF function over a plane defined by N(Bzi)‐Pt^2+^‐N(Bzi) atoms.

Ultimately, we further investigated the nature of the chemical interactions stabilizing the structure of the Stop‐[Bzi(**24C8**)‐Bipy(PtCl_2_)‐Bzi]‐Stop complex by investigating, with major detail, the topology of the *H*(***r***) function. In **Figure** **6**, we report the plot of the *H(**r**)* over either the *σ*[Cl‐Pt^2+^‐Cl] or *σ*[N(Bipy)‐Pt^2+^‐N(Bipy)] nanoscale plane, respectively; typical *H(**r**)* contour lines belonging to the pattern *±k* × *10* ^*n*^ (*k = *0,1,2,4,8; *n = *−5 to 6) are used accordingly to previous studies.^[^ ^34^, ^35^, ^36^ ^]^ In this regard, we would like to clarify that we used two different planes because we observed a slight variation (≈8 degrees) with respect to a perfect planar square local structure. The two analytical plots of the local *H(**r**)* function allow a visual discretization of the bonding motifs characterizing the observed Bipy(PtCl_2_) complex: i) as it can be observed in Figure 6a, the BCPs (reported using magenta dots) along the Pt^2+^—Cl bond paths are clearly immersed in a continuous region of negative values of *H(**r**); H(**r***_***cp***_ *)* equal to −0.026 and −0.023 hartree·*a*_*0*_^−3^ (see Table 2). ii) In essence, the same theoretical picture is observed looking at the 2D plot of the *H(**r**)* over the chelating site unit. As a matter of fact, also in this case, the graphical representation displayed in Figure 6b evidences that the estimated Pt^2+^‐N(Bipy) BCPs result confined in a region where the *H(**r**)* systematically samples negative values. As a result, the observed partitioning of the local space into regions of negative values along the detected bond paths—indicated here as *H* ^*−*^ *(**r**)—*can be distinctively correlated with the formation of a cooperative covalent bonding pattern.^[^ ^34^, ^35^, ^36^, ^37^, ^69^ ^]^ The same methodology was successfully used for estimating the nature of the mutual interactions characterizing a transient octahedral complex in the presence of a Zn(II) cation over the same chelating moiety.^[^ ^28^ ^]^ The existence of a covalently bound planar square complex is also supported by the electron localization function (ELF) plot reported in panels a,b of **Figure** **7**. As a matter of fact, at the derived C‐PCM(DMF)/B3LYP(D3) BCPs, the ELF features values no lesser than 0.36. Ultimately, the analyses reported were supported by further calculations carried out using a model system, namely a Bzi‐Bipy‐Bzi wire, and Douglas‐Kroll‐Hess 2nd order scalar relativistic calculations (see Table S1, and Figure S1 and S2, Supporting Information).

Finally, we would like to point out that the structures reported in this study were also characterized in terms of normal mode analysis. All the estimated frequencies are found to be > 0 as explicitly reported in the additional material. Interestingly, the computed normal modes for the PtCl_2_ moiety bound to organic Bipy chelating unit in a Bzi‐Bipy(PtCl_2_)‐Bzi model system in DMF solvent results in line with the literature:^[^ ^70^, ^71^, ^72^ ^]^ stretching frequencies are found at ≈308 cm^−1^ (asymmetric) and ≈320 cm^−1^ (symmetric). Furthermore, these signals are found at 312 and 326 cm^−1^ in the presence of the Stop‐[Bzi(**24C8**)‐Bipy(PtCl_2_)‐Bzi]‐Stop complex. Another interesting point to mention that has emerged is that the stretching of the N–H group of the Bzi stations might be useful for addressing the mutual position of the macrocycle along the Bzi‐Bipy‐Bzi axle. In fact, as highlighted by the calculated Infrared spectra reported as additional material, this normal mode results shifted toward red (≈3630 versus ≈3410 cm^−1^) due to the lack of complexation with the **24C8** macrocycle.

## 4 Conclusion

A recently proposed H‐shaped [2]rotaxane shuttle is addressed in CH_2_Cl_2_ solution via all‐atoms MD simulations and DFT‐based computations in conjunction with QTAIM descriptors. We focused on this synthetic supramolecular assembly since driven by the curiosity of addressing the nature of the chemical interactions opening up the fascinating possibility of modulating translocation mechanisms of interlocked macrocycles via metal cations. The modification of rotaxane structures through metal complexation and selective electrochemical reduction represents an intriguing perspective to enhance the functionalities of molecular machines and devices operating in liquid environments. The proposed computational scenario at room‐temperature conditions provides a reproduction of the conformational preferences of the **24C8** ring along the Stop‐[Bzi‐Bipy‐Bzi]‐Stop molecular axis. Moreover, the real time shuttling kinetic arising from the free‐energy landscape driving the reversible translocation of the macrocycle over the Stop‐[Bzi‐Bipy‐Bzi]‐Stop thread is also well reproduced (ΔG^‡^ of 15.1 versus 11.2 kcal mol^−1^) with an umbrella sampling method. Further analyses suggest that a labile supramolecular aggregate can be appreciated at the middle point along the shuttling movement connecting the ether ring with the central Bipy unit. Also, we extended our combined C‐PCM/DFT+QTAIM analysis to a translationally inactive form in DMF following the coordination of a PtCl_2_ moiety to the Bipy chelate site. From these data, we obtain that the contextual presence of supramolecular contacts confining the **24C8** ring over its primary recognition site, and, of a planar square (Bipy)‐N_2_‐Pt^2+^Cl_2_ complex parallel to the axle and held together by covalent chemical bonds should be actually effective in suppressing the shutting movement as hypothesized via ^1^ H‐NMR measurements. In summary, the proposed work suggests once again that theoretical modeling can be useful for obtaining—in a computationally affordable and relatively accurate way—analytical information finely modulating the conformational shaping of molecular machines and devices of a certain complexity. Consequently, we expect that these investigation methods will become part of a more general protocol that will enable the identification of molecular systems for advanced nanotechnological applications with progressively higher efficiency and functionalities.

## Supporting Information

Optimized geometries at C‐PCM/DFT level also in the presence of Pt(II), classical molecular force fields applied, QTAIM derived properties and 3D plot of the *H(0,ISO)* of the Stop‐[Bzi(**24C8**)‐Bipy(PtCl_2_)‐Bzi]‐Stop complex.

## Conflict of Interest

The authors declare no conflict of interest.

## Supporting information

Supplementary Material

## Data Availability

The data that support the findings of this study are available in the supplementary material of this article.
