# Supplementary material for: A Computational Study of a [2]Rotaxane Molecular Shuttle with All‐Atoms Molecular Dynamics and Density Functional Theory Simulations in Solution
Source: Chemphyschem. 2025 Nov 18;27(1):e202500660. doi: 10.1002/cphc.202500660 (PMC12810620; doi:10.1002/cphc.202500660)
Supplement: Supplementary file 1 — Supplementary Material [file CPHC-27-e202500660-s001.pdf]

A Computational Study of a [2]Rotaxane Molecular Shuttle with all-Atoms MD and DFT Simulations in Solution

Costantino Zazza,*[a] Nico Sanna,[a,b] Stefano Borocci[a,c] and Felice Grandinetti[a,c]

[a] Dr. Costantino Zazza, Prof. Nico Sanna, Prof. Stefano Borocci, and Prof. Felice Grandinetti
Department for Innovation in Biological, Agro-food and Forest systems, Università della Tuscia (DIBAF), L.go dell'Università, s.n.c., 01100 Viterbo, Italy.
E-mail: costantino.zazza@unitus.it

[b] Prof. Nico Sanna
CNR-ISTP (Istituto per la Scienza e Tecnologia dei Plasmi), Via G. Amendola 122/D, 70126 Bari, Italy.

[c] Prof. Stefano Borocci and Prof. Felice Grandinetti
Istituto per i Sistemi Biologici del CNR (ISB), Sede di Roma - Meccanismi di Reazione c/o Dipartimento di Chimica, Sapienza Università di Roma, P.le A. Moro 5, Rome (Italy).

Electronic Supporting Information (ESI)

Table S1. Electron density $\rho(\mathbf{r})$ ($e\cdot a_0^{-3}$), Laplacian of electron density $\nabla^2\rho(\mathbf{r})$ ($e\cdot a_0^{-5}$), electron kinetic energy density $G(\mathbf{r})$ (hartree$\cdot a_0^{-3}$), electron potential energy density $V(\mathbf{r})$ (hartree$\cdot a_0^{-3}$) and electron energy density $H(\mathbf{r})$ (hartree$\cdot a_0^{-3}$) for bond critical points on selected bonds of the optimized Bzi-Bipy($PtCl_2$)-Bzi model system (see Figure S1) of the investigated [2]molecular rotaxane at C-PCM(DMF)/B3LYP(D3,DKH2)/def2-TZVP level. Data refer to geometrical structure shown in Fig. S1.

BCP [a]	Distance (Å)	$\rho(\mathbf{r})$	$\nabla^2\rho(\mathbf{r})$	$G(\mathbf{r})$	$V(\mathbf{r})$	$-G(\mathbf{r})/V(\mathbf{r})$	$H(\mathbf{r})$
Pt(II) – N	2.04	0.191	0.088	0.209	-0.398	0.524	-0.189
Pt(II) – N	2.04	0.198	0.088	0.200	-0.406	0.494	-0.205
Pt(II) – Cl	2.35	0.121	0.431	0.149	-0.189	0.787	-0.040
Pt(II) – Cl	2.36	0.121	0.433	0.148	-0.189	0.786	-0.040

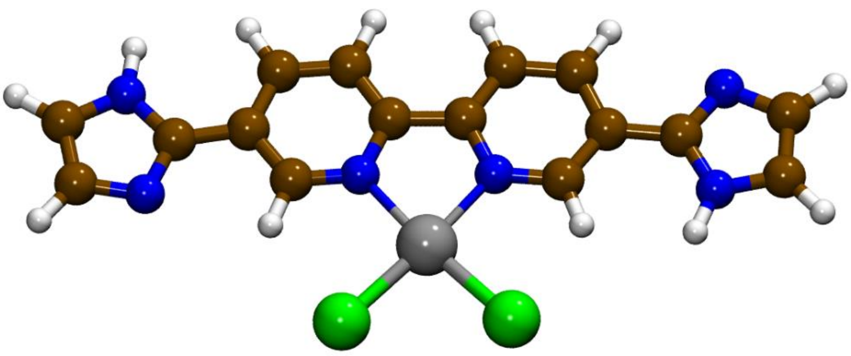

Figure S1. Bzi-Bipy($PtCl_2$)-Bzi model system at C-PCM(DMF)/B3LYP(D3,DKH2)/def2-TZVP level of theory. This system was simulated with an all-electrons approach for the Pt atom with an imposed def2-TZVP basis set and a relativistic hamiltonian (Douglas–Kroll approximation, 2nd order).

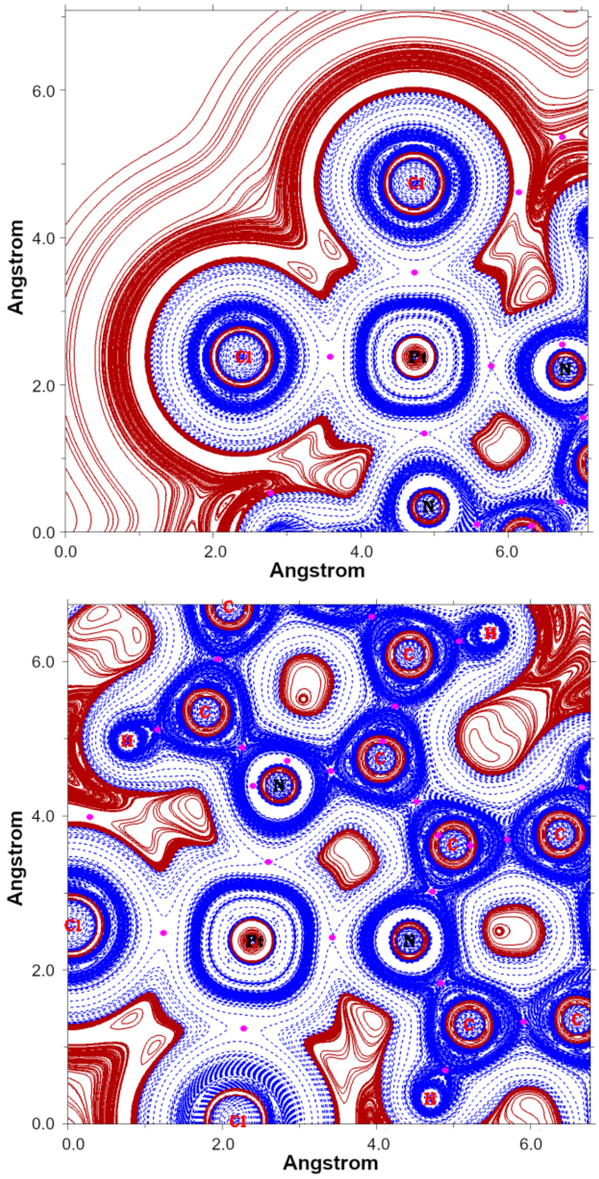

Figure S2. 2D-plot (in hartree·a_0^{-3}) of the C-PCM-B3LYP(D3,DKH2)/def2-TZVP *H(**r**)* in the σ plane defined by the position of Cl-Pt^{2+}-Cl atoms in the optimized geometry of the Bzi-Bipy($PtCl_2$)-Bzi complex model. Solid/brown and dashed/blue lines correspond to positive and negative values of the *H(**r**)* function. The dots in magenta indicate the CPs (+3,-1) from the topological analysis of the *H(**r**)*; b) 2D-plot of the *H(**r**)* function over a plane defined by N(Bzi)-Pt^{2+}-N(Bzi) atoms.

Optimized geometry - at C-PCM(CH_2Cl_2)/B3LYP(D3)/cc-pVTZ level of theory - of the investigated Stop-[Bzi(24C8)-Bipy-Bzi]-Stop [2] rotaxane molecular shuttle:

Atom list:

1(N) --> Charge: 7.000000 x,y,z(Bohr): 13.018760 1.584755 -0.944829
2(N) --> Charge: 7.000000 x,y,z(Bohr): 12.918621 -2.296605 0.747056
3(H) --> Charge: 1.000000 x,y,z(Bohr): 12.233021 -3.874883 1.576368
4(N) --> Charge: 7.000000 x,y,z(Bohr): 4.757690 -2.267552 0.497470
5(N) --> Charge: 7.000000 x,y,z(Bohr): -0.415638 1.871252 -1.217479
6(N) --> Charge: 7.000000 x,y,z(Bohr): -8.309821 2.199010 0.698095
7(H) --> Charge: 1.000000 x,y,z(Bohr): -7.371438 3.766927 1.311914
8(N) --> Charge: 7.000000 x,y,z(Bohr): -8.835607 -1.648743 -0.998883
9(C) --> Charge: 6.000000 x,y,z(Bohr): 3.559057 -0.199806 -0.382592
10(C) --> Charge: 6.000000 x,y,z(Bohr): -14.205748 -13.810812 -2.310058
11(H) --> Charge: 1.000000 x,y,z(Bohr): -12.345479 -13.514667 -1.420048
12(H) --> Charge: 1.000000 x,y,z(Bohr): -14.342993 -15.811680 -2.878495
13(H) --> Charge: 1.000000 x,y,z(Bohr): -15.661663 -13.458331 -0.866719

14(C) --> Charge: 6.000000 x,y,z(Bohr): 4.895653 1.934505 -1.251233
15(H) --> Charge: 1.000000 x,y,z(Bohr): 3.830256 3.546889 -1.958403
16(C) --> Charge: 6.000000 x,y,z(Bohr): 7.514772 1.922078 -1.186204
17(H) --> Charge: 1.000000 x,y,z(Bohr): 8.624338 3.530012 -1.843870
18(C) --> Charge: 6.000000 x,y,z(Bohr): 8.783330 -0.223027 -0.255597
19(C) --> Charge: 6.000000 x,y,z(Bohr): 7.274125 -2.257847 0.545367
20(H) --> Charge: 1.000000 x,y,z(Bohr): 8.139748 -3.995830 1.268019
21(C) --> Charge: 6.000000 x,y,z(Bohr): 11.543572 -0.272502 -0.163187
22(C) --> Charge: 6.000000 x,y,z(Bohr): 15.470863 0.781525 -0.516167
23(C) --> Charge: 6.000000 x,y,z(Bohr): 15.449171 -1.679808 0.550540
24(C) --> Charge: 6.000000 x,y,z(Bohr): 17.637260 -3.020754 1.237361
25(C) --> Charge: 6.000000 x,y,z(Bohr): 19.905203 -1.723432 0.801565
26(H) --> Charge: 1.000000 x,y,z(Bohr): 21.683844 -2.653741 1.280340
27(C) --> Charge: 6.000000 x,y,z(Bohr): 19.974112 0.706599 -0.268018
28(H) --> Charge: 1.000000 x,y,z(Bohr): 21.812748 1.604566 -0.524887
29(C) --> Charge: 6.000000 x,y,z(Bohr): 17.792113 2.039748 -0.974789
30(C) --> Charge: 6.000000 x,y,z(Bohr): 16.099210 6.446166 -1.653609
31(H) --> Charge: 1.000000 x,y,z(Bohr): 14.491943 5.991038 -0.451915
32(C) --> Charge: 6.000000 x,y,z(Bohr): 16.295549 8.859822 -2.705527
33(H) --> Charge: 1.000000 x,y,z(Bohr): 14.817639 10.224074 -2.262669
34(C) --> Charge: 6.000000 x,y,z(Bohr): 18.316337 9.537975 -4.282125
35(C) --> Charge: 6.000000 x,y,z(Bohr): 20.141893 7.663841 -4.761122
36(H) --> Charge: 1.000000 x,y,z(Bohr): 21.740830 8.078490 -5.998361
37(C) --> Charge: 6.000000 x,y,z(Bohr): 19.963789 5.256793 -3.714327
38(H) --> Charge: 1.000000 x,y,z(Bohr): 21.399591 3.850640 -4.183627
39(C) --> Charge: 6.000000 x,y,z(Bohr): 17.938581 4.589236 -2.120753
40(C) --> Charge: 6.000000 x,y,z(Bohr): 18.587382 12.172138 -5.480249
41(C) --> Charge: 6.000000 x,y,z(Bohr): 18.586877 11.916035 -8.390184
42(H) --> Charge: 1.000000 x,y,z(Bohr): 20.155259 10.725577 -9.061346
43(H) --> Charge: 1.000000 x,y,z(Bohr): 18.784199 13.788370 -9.284869
44(H) --> Charge: 1.000000 x,y,z(Bohr): 16.809569 11.060053 -9.056465
45(C) --> Charge: 6.000000 x,y,z(Bohr): 21.103433 13.376264 -4.613205
46(H) --> Charge: 1.000000 x,y,z(Bohr): 21.149824 13.581813 -2.541597
47(H) --> Charge: 1.000000 x,y,z(Bohr): 21.334804 15.266583 -5.460524
48(H) --> Charge: 1.000000 x,y,z(Bohr): 22.744145 12.226872 -5.173979
49(C) --> Charge: 6.000000 x,y,z(Bohr): 16.419647 13.957950 -4.726250
50(H) --> Charge: 1.000000 x,y,z(Bohr): 14.570008 13.230832 -5.344552
51(H) --> Charge: 1.000000 x,y,z(Bohr): 16.695664 15.816917 -5.622020
52(H) --> Charge: 1.000000 x,y,z(Bohr): 16.346273 14.256300 -2.667545
53(C) --> Charge: 6.000000 x,y,z(Bohr): 17.538474 -5.582030 2.359958
54(C) --> Charge: 6.000000 x,y,z(Bohr): 19.068457 -6.241888 4.423197
55(H) --> Charge: 1.000000 x,y,z(Bohr): 20.326750 -4.824104 5.239836
56(C) --> Charge: 6.000000 x,y,z(Bohr): 18.976033 -8.659069 5.482948
57(H) --> Charge: 1.000000 x,y,z(Bohr): 20.199307 -9.056376 7.091052
58(C) --> Charge: 6.000000 x,y,z(Bohr): 17.350462 -10.531260 4.541322
59(C) --> Charge: 6.000000 x,y,z(Bohr): 15.826702 -9.863573 2.466172
60(H) --> Charge: 1.000000 x,y,z(Bohr): 14.549797 -11.258009 1.639500
61(C) --> Charge: 6.000000 x,y,z(Bohr): 15.914767 -7.459021 1.396039
62(H) --> Charge: 1.000000 x,y,z(Bohr): 14.754112 -7.049669 -0.262689
63(C) --> Charge: 6.000000 x,y,z(Bohr): 17.187396 -13.207859 5.663226
64(C) --> Charge: 6.000000 x,y,z(Bohr): 0.750476 -0.250954 -0.406159
65(C) --> Charge: 6.000000 x,y,z(Bohr): -0.590371 -2.406031 0.369311
66(H) --> Charge: 1.000000 x,y,z(Bohr): 0.454736 -4.074282 0.967717
67(C) --> Charge: 6.000000 x,y,z(Bohr): -3.219099 -2.351592 0.363523
68(H) --> Charge: 1.000000 x,y,z(Bohr): -4.357705 -3.951234 0.974957
69(C) --> Charge: 6.000000 x,y,z(Bohr): -4.428103 -0.135527 -0.418434
70(C) --> Charge: 6.000000 x,y,z(Bohr): -2.939025 1.901560 -1.235758
71(H) --> Charge: 1.000000 x,y,z(Bohr): -3.849421 3.613308 -1.939214
72(C) --> Charge: 6.000000 x,y,z(Bohr): -7.182475 0.085750 -0.302050
73(C) --> Charge: 6.000000 x,y,z(Bohr): -10.878989 1.787602 0.686606
74(C) --> Charge: 6.000000 x,y,z(Bohr): -11.177401 -0.628714 -0.445320
75(C) --> Charge: 6.000000 x,y,z(Bohr): -13.613508 -1.665382 -0.833673
76(C) --> Charge: 6.000000 x,y,z(Bohr): -15.650224 -0.158631 -0.045329
77(H) --> Charge: 1.000000 x,y,z(Bohr): -17.573236 -0.848888 -0.334134
78(C) --> Charge: 6.000000 x,y,z(Bohr): -15.317821 2.220370 1.096294

79(H) --> Charge: 1.000000 x,y,z(Bohr): -16.982530 3.271889 1.715800
80(C) --> Charge: 6.000000 x,y,z(Bohr): -12.920456 3.266860 1.530351
81(C) --> Charge: 6.000000 x,y,z(Bohr): -12.509845 5.730517 2.789614
82(C) --> Charge: 6.000000 x,y,z(Bohr): -13.985636 7.863670 2.206679
83(H) --> Charge: 1.000000 x,y,z(Bohr): -15.489100 7.698801 0.801240
84(C) --> Charge: 6.000000 x,y,z(Bohr): -13.508823 10.193606 3.338902
85(H) --> Charge: 1.000000 x,y,z(Bohr): -14.679546 11.805170 2.798467
86(C) --> Charge: 6.000000 x,y,z(Bohr): -11.552629 10.514816 5.114464
87(C) --> Charge: 6.000000 x,y,z(Bohr): -10.119672 8.367380 5.718837
88(H) --> Charge: 1.000000 x,y,z(Bohr): -8.587919 8.474456 7.089982
89(C) --> Charge: 6.000000 x,y,z(Bohr): -10.571872 6.032297 4.577164
90(H) --> Charge: 1.000000 x,y,z(Bohr): -9.380255 4.428773 5.087135
91(C) --> Charge: 6.000000 x,y,z(Bohr): -11.028990 13.138610 6.248540
92(C) --> Charge: 6.000000 x,y,z(Bohr): -13.426049 14.129233 7.589000
93(H) --> Charge: 1.000000 x,y,z(Bohr): -15.033718 14.295058 6.279575
94(H) --> Charge: 1.000000 x,y,z(Bohr): -13.077118 16.015852 8.402686
95(H) --> Charge: 1.000000 x,y,z(Bohr): -13.991719 12.850115 9.131349
96(C) --> Charge: 6.000000 x,y,z(Bohr): -10.287988 14.971981 4.097310
97(H) --> Charge: 1.000000 x,y,z(Bohr): -8.575396 14.304732 3.116323
98(H) --> Charge: 1.000000 x,y,z(Bohr): -9.899627 16.873213 4.857845
99(H) --> Charge: 1.000000 x,y,z(Bohr): -11.801625 15.151561 2.681387
100(C) --> Charge: 6.000000 x,y,z(Bohr): -8.859100 13.089042 8.184173
101(H) --> Charge: 1.000000 x,y,z(Bohr): -9.281547 11.828108 9.785128
102(H) --> Charge: 1.000000 x,y,z(Bohr): -8.553422 15.000567 8.949276
103(H) --> Charge: 1.000000 x,y,z(Bohr): -7.068596 12.466051 7.323643
104(C) --> Charge: 6.000000 x,y,z(Bohr): -13.932947 -4.222922 -1.922776
105(C) --> Charge: 6.000000 x,y,z(Bohr): -12.226955 -5.179050 -3.720425
106(H) --> Charge: 1.000000 x,y,z(Bohr): -10.662821 -4.001676 -4.347115
107(C) --> Charge: 6.000000 x,y,z(Bohr): -12.429917 -7.651987 -4.612682
108(H) --> Charge: 1.000000 x,y,z(Bohr): -11.027193 -8.293562 -5.976688
109(C) --> Charge: 6.000000 x,y,z(Bohr): -14.328639 -9.294325 -3.764144
110(C) --> Charge: 6.000000 x,y,z(Bohr): -16.059263 -8.315044 -1.995538
111(H) --> Charge: 1.000000 x,y,z(Bohr): -17.570517 -9.514907 -1.262808
112(C) --> Charge: 6.000000 x,y,z(Bohr): -15.872226 -5.847894 -1.096024
113(H) --> Charge: 1.000000 x,y,z(Bohr): -17.206506 -5.195371 0.337401
114(C) --> Charge: 6.000000 x,y,z(Bohr): -14.531803 -12.061517 -4.627278
115(C) --> Charge: 6.000000 x,y,z(Bohr): -12.493246 -12.759947 -6.580603
116(H) --> Charge: 1.000000 x,y,z(Bohr): -10.573706 -12.534767 -5.806212
117(H) --> Charge: 1.000000 x,y,z(Bohr): -12.639934 -11.594418 -8.298797
118(H) --> Charge: 1.000000 x,y,z(Bohr): -12.712106 -14.751170 -7.147303
119(C) --> Charge: 6.000000 x,y,z(Bohr): -17.149767 -12.524770 -5.834488
120(H) --> Charge: 1.000000 x,y,z(Bohr): -17.326746 -14.507367 -6.453133
121(H) --> Charge: 1.000000 x,y,z(Bohr): -17.424764 -11.298077 -7.494353
122(H) --> Charge: 1.000000 x,y,z(Bohr): -18.696310 -12.139763 -4.497731
123(C) --> Charge: 6.000000 x,y,z(Bohr): 18.984516 -13.569864 7.920534
124(H) --> Charge: 1.000000 x,y,z(Bohr): 18.801420 -15.506798 8.660192
125(H) --> Charge: 1.000000 x,y,z(Bohr): 18.544485 -12.259715 9.477041
126(H) --> Charge: 1.000000 x,y,z(Bohr): 20.974414 -13.292010 7.376843
127(C) --> Charge: 6.000000 x,y,z(Bohr): 17.895965 -15.160420 3.609288
128(H) --> Charge: 1.000000 x,y,z(Bohr): 19.836062 -14.842861 2.923442
129(H) --> Charge: 1.000000 x,y,z(Bohr): 16.618078 -15.059381 1.970865
130(H) --> Charge: 1.000000 x,y,z(Bohr): 17.785928 -17.093962 4.378726
131(C) --> Charge: 6.000000 x,y,z(Bohr): 14.459030 -13.707780 6.578783
132(H) --> Charge: 1.000000 x,y,z(Bohr): 14.304023 -15.623116 7.385016
133(H) --> Charge: 1.000000 x,y,z(Bohr): 13.082446 -13.564256 5.025579
134(H) --> Charge: 1.000000 x,y,z(Bohr): 13.907600 -12.338541 8.047480
135(O) --> Charge: 8.000000 x,y,z(Bohr): -6.717262 -7.124534 -1.737068
136(C) --> Charge: 6.000000 x,y,z(Bohr): -5.273181 -8.216051 -3.685130
137(H) --> Charge: 1.000000 x,y,z(Bohr): -6.266515 -9.870688 -4.518159
138(H) --> Charge: 1.000000 x,y,z(Bohr): -3.411772 -8.871756 -2.974427
139(C) --> Charge: 6.000000 x,y,z(Bohr): -4.876072 -6.245945 -5.730506
140(H) --> Charge: 1.000000 x,y,z(Bohr): -3.953594 -7.155898 -7.383532
141(H) --> Charge: 1.000000 x,y,z(Bohr): -6.739805 -5.517792 -6.339300
142(O) --> Charge: 8.000000 x,y,z(Bohr): -3.351535 -4.299834 -4.730740
143(C) --> Charge: 6.000000 x,y,z(Bohr): -2.335715 -2.570857 -6.491393

144(H) --> Charge: 1.000000 x,y,z(Bohr): -1.109989 -1.314733 -5.378727
145(H) --> Charge: 1.000000 x,y,z(Bohr): -1.145125 -3.570156 -7.906401
146(C) --> Charge: 6.000000 x,y,z(Bohr): -4.239714 -0.977809 -7.946848
147(H) --> Charge: 1.000000 x,y,z(Bohr): -5.520884 -2.193449 -9.082232
148(H) --> Charge: 1.000000 x,y,z(Bohr): -3.160359 0.197276 -9.312979
149(O) --> Charge: 8.000000 x,y,z(Bohr): -5.657640 0.560686 -6.286331
150(C) --> Charge: 6.000000 x,y,z(Bohr): -7.275332 2.230410 -7.604305
151(H) --> Charge: 1.000000 x,y,z(Bohr): -6.162680 3.558109 -8.787801
152(H) --> Charge: 1.000000 x,y,z(Bohr): -8.551561 1.156236 -8.878899
153(C) --> Charge: 6.000000 x,y,z(Bohr): -8.894094 3.722505 -5.780393
154(H) --> Charge: 1.000000 x,y,z(Bohr): -10.395512 4.686031 -6.884214
155(H) --> Charge: 1.000000 x,y,z(Bohr): -9.821064 2.423555 -4.432587
156(O) --> Charge: 8.000000 x,y,z(Bohr): -7.397327 5.535907 -4.483309
157(C) --> Charge: 6.000000 x,y,z(Bohr): -8.886078 7.213750 -3.022254
158(H) --> Charge: 1.000000 x,y,z(Bohr): -10.271980 6.170734 -1.867235
159(H) --> Charge: 1.000000 x,y,z(Bohr): -9.964157 8.521896 -4.261162
160(C) --> Charge: 6.000000 x,y,z(Bohr): -7.195699 8.765942 -1.315786
161(H) --> Charge: 1.000000 x,y,z(Bohr): -5.800751 9.831633 -2.458205
162(H) --> Charge: 1.000000 x,y,z(Bohr): -8.380304 10.130497 -0.257989
163(O) --> Charge: 8.000000 x,y,z(Bohr): -5.913250 7.122854 0.374736
164(C) --> Charge: 6.000000 x,y,z(Bohr): -4.409982 8.377284 2.200125
165(H) --> Charge: 1.000000 x,y,z(Bohr): -5.632350 9.357157 3.584820
166(H) --> Charge: 1.000000 x,y,z(Bohr): -3.175205 9.803559 1.289714
167(C) --> Charge: 6.000000 x,y,z(Bohr): -2.758326 6.431065 3.489816
168(H) --> Charge: 1.000000 x,y,z(Bohr): -1.490253 5.544608 2.086280
169(H) --> Charge: 1.000000 x,y,z(Bohr): -1.567292 7.389708 4.927043
170(O) --> Charge: 8.000000 x,y,z(Bohr): -4.317788 4.575297 4.646980
171(C) --> Charge: 6.000000 x,y,z(Bohr): -2.872533 2.526991 5.620272
172(H) --> Charge: 1.000000 x,y,z(Bohr): -1.384111 3.256539 6.904734
173(H) --> Charge: 1.000000 x,y,z(Bohr): -1.937306 1.485812 4.071441
174(C) --> Charge: 6.000000 x,y,z(Bohr): -4.532465 0.744815 7.114815
175(H) --> Charge: 1.000000 x,y,z(Bohr): -3.277792 -0.557305 8.178532
176(H) --> Charge: 1.000000 x,y,z(Bohr): -5.649678 1.834374 8.518441
177(O) --> Charge: 8.000000 x,y,z(Bohr): -6.144161 -0.642507 5.494554
178(C) --> Charge: 6.000000 x,y,z(Bohr): -7.467478 -2.563396 6.816544
179(H) --> Charge: 1.000000 x,y,z(Bohr): -8.701786 -1.709170 8.282290
180(H) --> Charge: 1.000000 x,y,z(Bohr): -6.116645 -3.843714 7.782257
181(C) --> Charge: 6.000000 x,y,z(Bohr): -9.093761 -4.099322 5.036855
182(H) --> Charge: 1.000000 x,y,z(Bohr): -10.435123 -5.218832 6.201148
183(H) --> Charge: 1.000000 x,y,z(Bohr): -10.217394 -2.828444 3.818681
184(O) --> Charge: 8.000000 x,y,z(Bohr): -7.569073 -5.737052 3.564095
185(C) --> Charge: 6.000000 x,y,z(Bohr): -9.055367 -7.417869 2.087475
186(H) --> Charge: 1.000000 x,y,z(Bohr): -10.522942 -6.380466 1.035014
187(H) --> Charge: 1.000000 x,y,z(Bohr): -10.001797 -8.816277 3.334977
188(C) --> Charge: 6.000000 x,y,z(Bohr): -7.403206 -8.809457 0.211647
189(H) --> Charge: 1.000000 x,y,z(Bohr): -5.708462 -9.582272 1.178439
190(H) --> Charge: 1.000000 x,y,z(Bohr): -8.497473 -10.424226 -0.566143

Note: Orbital   341 is HOMO, energy: -0.191493 a.u.   -5.210777 eV
Orbital   342 is LUMO, energy: -0.066755 a.u.   -1.816499 eV
HOMO-LUMO gap: 0.124737 a.u.   3.394278 eV   327.498085 kJ/mol

Molecular geometry of the transient species at the midpoint along the shuttling translocation of the 24C8 ring investigated at C-PCM(CH_2Cl_2)/B3LYP(D3)/cc-pVTZ level of theory. In this configuration - sampled during MD sampling with the umbrella sampling technique and then optimized at C-PCM/DFT – the ether ring guests the Bipy central unit (see Figures 3 and 4 into the associated manuscript). AIM descriptors are then subsequently estimated and reported in Table 1.

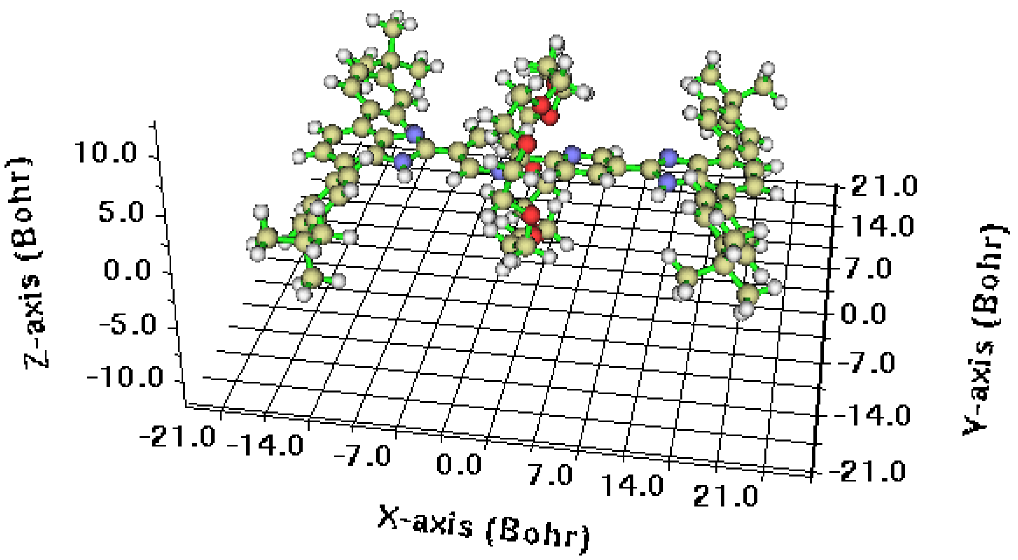

Atom list:

1(H) --> Charge: 1.000000 x,y,z(Bohr): -1.417292   3.771705   0.526774
2(C) --> Charge: 6.000000 x,y,z(Bohr): -2.520086   2.045404   0.389672
3(C) --> Charge: 6.000000 x,y,z(Bohr): -5.139830   2.075605   0.397288
4(C) --> Charge: 6.000000 x,y,z(Bohr): -1.257041 -0.287655   0.217614
5(H) --> Charge: 1.000000 x,y,z(Bohr): -6.191187   3.841388   0.552037
6(C) --> Charge: 6.000000 x,y,z(Bohr): -6.480264 -0.212190   0.199453
7(N) --> Charge: 7.000000 x,y,z(Bohr): -2.516774 -2.493271   0.077183
8(C) --> Charge: 6.000000 x,y,z(Bohr):   1.544175 -0.367515   0.191421
9(C) --> Charge: 6.000000 x,y,z(Bohr): -5.037615 -2.436322   0.057733
10(C) --> Charge: 6.000000 x,y,z(Bohr): -9.242536 -0.196498   0.151622
11(N) --> Charge: 7.000000 x,y,z(Bohr):   2.732386   1.819298 -0.353524
12(C) --> Charge: 6.000000 x,y,z(Bohr):   2.865664 -2.607129   0.699998
13(H) --> Charge: 1.000000 x,y,z(Bohr): -5.955989 -4.287204 -0.066936
14(N) --> Charge: 7.000000 x,y,z(Bohr): -10.633442   1.880800   0.268321
15(N) --> Charge: 7.000000 x,y,z(Bohr): -10.705659 -2.343244 -0.018844
16(C) --> Charge: 6.000000 x,y,z(Bohr):   5.247836   1.847181 -0.373720
17(H) --> Charge: 1.000000 x,y,z(Bohr):   1.849428 -4.332446   1.129772
18(C) --> Charge: 6.000000 x,y,z(Bohr):   5.487934 -2.559440   0.677146
19(C) --> Charge: 6.000000 x,y,z(Bohr): -13.120396   1.081302   0.145237
20(H) --> Charge: 1.000000 x,y,z(Bohr): -10.087096 -4.140029 -0.231751
21(C) --> Charge: 6.000000 x,y,z(Bohr): -13.207570 -1.596240 -0.019481
22(H) --> Charge: 1.000000 x,y,z(Bohr):   6.160334   3.641258 -0.835526
23(C) --> Charge: 6.000000 x,y,z(Bohr):   6.753846 -0.285745   0.137627
24(H) --> Charge: 1.000000 x,y,z(Bohr):   6.529715 -4.286697   1.116265
25(C) --> Charge: 6.000000 x,y,z(Bohr): -15.380771   2.521315   0.154223
26(C) --> Charge: 6.000000 x,y,z(Bohr): -15.463571 -2.995658 -0.179967
27(C) --> Charge: 6.000000 x,y,z(Bohr):   9.506821 -0.059160   0.088891
28(C) --> Charge: 6.000000 x,y,z(Bohr): -17.628591   1.123724 -0.021852
29(C) --> Charge: 6.000000 x,y,z(Bohr): -15.392699   5.316492   0.333619
30(C) --> Charge: 6.000000 x,y,z(Bohr): -17.671457 -1.528827 -0.187717
31(C) --> Charge: 6.000000 x,y,z(Bohr): -15.500256 -5.790009 -0.350914
32(N) --> Charge: 7.000000 x,y,z(Bohr): 10.761202   2.105408   0.008546
33(N) --> Charge: 7.000000 x,y,z(Bohr): 11.105330 -2.114649   0.137282
34(H) --> Charge: 1.000000 x,y,z(Bohr): -19.428331   2.130255 -0.066749
35(C) --> Charge: 6.000000 x,y,z(Bohr): -13.514257   6.803004 -0.816038
36(C) --> Charge: 6.000000 x,y,z(Bohr): -17.321814   6.597723   1.649070
37(H) --> Charge: 1.000000 x,y,z(Bohr): -19.495933 -2.485970 -0.298283
38(C) --> Charge: 6.000000 x,y,z(Bohr): -17.156764 -7.042692 -2.013988
39(C) --> Charge: 6.000000 x,y,z(Bohr): -13.904676 -7.295181   1.146357

40(C) --> Charge: 6.000000 x,y,z(Bohr): 13.296407   1.457696 -0.016808
41(H) --> Charge: 1.000000 x,y,z(Bohr): 10.594304 -3.952584 -0.001932
42(C) --> Charge: 6.000000 x,y,z(Bohr): 13.554614 -1.213254   0.058185
43(H) --> Charge: 1.000000 x,y,z(Bohr): -11.986911   5.882776 -1.844426
44(C) --> Charge: 6.000000 x,y,z(Bohr): -13.571059   9.440037 -0.667763
45(H) --> Charge: 1.000000 x,y,z(Bohr): -18.792011   5.518000   2.614643
46(C) --> Charge: 6.000000 x,y,z(Bohr): -17.363386   9.226555   1.792298
47(H) --> Charge: 1.000000 x,y,z(Bohr): -18.405627 -5.935671 -3.228445
48(C) --> Charge: 6.000000 x,y,z(Bohr): -17.193639 -9.670608 -2.169069
49(H) --> Charge: 1.000000 x,y,z(Bohr): -12.644100 -6.406604   2.519252
50(C) --> Charge: 6.000000 x,y,z(Bohr): -13.947713 -9.932786   0.976748
51(C) --> Charge: 6.000000 x,y,z(Bohr): 15.460673   3.031345 -0.173238
52(C) --> Charge: 6.000000 x,y,z(Bohr): 15.893171 -2.477356 -0.022730
53(H) --> Charge: 1.000000 x,y,z(Bohr): -12.068622 10.488424 -1.608575
54(C) --> Charge: 6.000000 x,y,z(Bohr): -15.490599 10.724600   0.637369
55(H) --> Charge: 1.000000 x,y,z(Bohr): -18.891816 10.124204   2.848892
56(H) --> Charge: 1.000000 x,y,z(Bohr): -18.501621 -10.552874 -3.498610
57(C) --> Charge: 6.000000 x,y,z(Bohr): -15.586980 -11.190219 -0.685899
58(H) --> Charge: 1.000000 x,y,z(Bohr): -12.674849 -10.998637   2.194432
59(C) --> Charge: 6.000000 x,y,z(Bohr): 17.791397   1.766373 -0.267637
60(C) --> Charge: 6.000000 x,y,z(Bohr): 15.298725   5.826190 -0.254291
61(C) --> Charge: 6.000000 x,y,z(Bohr): 18.002753 -0.881576 -0.201636
62(C) --> Charge: 6.000000 x,y,z(Bohr): 16.108723 -5.267259   0.061030
63(C) --> Charge: 6.000000 x,y,z(Bohr): -15.605424 13.621044   0.850769
64(C) --> Charge: 6.000000 x,y,z(Bohr): -15.684763 -14.084552 -0.933725
65(H) --> Charge: 1.000000 x,y,z(Bohr): 19.527570   2.877840 -0.332890
66(C) --> Charge: 6.000000 x,y,z(Bohr): 13.430571   7.161910   1.081670
67(C) --> Charge: 6.000000 x,y,z(Bohr): 17.044551   7.261207 -1.664299
68(H) --> Charge: 1.000000 x,y,z(Bohr): 19.884647 -1.727007 -0.225174
69(C) --> Charge: 6.000000 x,y,z(Bohr): 17.835969 -6.557802 -1.485907
70(C) --> Charge: 6.000000 x,y,z(Bohr): 14.607755 -6.729999   1.703803
71(C) --> Charge: 6.000000 x,y,z(Bohr): -15.486372 14.385932   3.667952
72(C) --> Charge: 6.000000 x,y,z(Bohr): -13.405253 14.918815 -0.539165
73(C) --> Charge: 6.000000 x,y,z(Bohr): -18.111030 14.577143 -0.307474
74(H) --> Charge: 1.000000 x,y,z(Bohr): -17.069702 13.584886   4.753859
75(H) --> Charge: 1.000000 x,y,z(Bohr): -15.571757 16.459377   3.857341
76(H) --> Charge: 1.000000 x,y,z(Bohr): -13.712846 13.727178   4.538787
77(H) --> Charge: 1.000000 x,y,z(Bohr): -13.570211 16.983684 -0.335808
78(H) --> Charge: 1.000000 x,y,z(Bohr): -13.413254 14.483591 -2.574650
79(H) --> Charge: 1.000000 x,y,z(Bohr): -11.559066 14.355179   0.239734
80(H) --> Charge: 1.000000 x,y,z(Bohr): -18.240401 14.059519 -2.320670
81(H) --> Charge: 1.000000 x,y,z(Bohr): -18.227891 16.652539 -0.162123
82(H) --> Charge: 1.000000 x,y,z(Bohr): -19.767236 13.780149   0.666966
83(C) --> Charge: 6.000000 x,y,z(Bohr): -18.370814 -15.019312 -0.266230
84(C) --> Charge: 6.000000 x,y,z(Bohr): -13.792270 -15.407757   0.833718
85(C) --> Charge: 6.000000 x,y,z(Bohr): -15.050315 -14.838513 -3.684060
86(H) --> Charge: 1.000000 x,y,z(Bohr): -19.809973 -14.203946 -1.527894
87(H) --> Charge: 1.000000 x,y,z(Bohr): -18.867891 -14.507077   1.690251
88(H) --> Charge: 1.000000 x,y,z(Bohr): -18.476189 -17.092815 -0.441279
89(H) --> Charge: 1.000000 x,y,z(Bohr): -13.930523 -17.469709   0.587468
90(H) --> Charge: 1.000000 x,y,z(Bohr): -11.829170 -14.854536   0.417406
91(H) --> Charge: 1.000000 x,y,z(Bohr): -14.178391 -14.986256   2.835208
92(H) --> Charge: 1.000000 x,y,z(Bohr): -15.118172 -16.910054 -3.896672
93(H) --> Charge: 1.000000 x,y,z(Bohr): -16.396477 -14.018948 -5.041950
94(H) --> Charge: 1.000000 x,y,z(Bohr): -13.139589 -14.194897 -4.205936
95(H) --> Charge: 1.000000 x,y,z(Bohr): 12.036369   6.119777   2.180631
96(C) --> Charge: 6.000000 x,y,z(Bohr): 13.325194   9.800957   1.023433
97(H) --> Charge: 1.000000 x,y,z(Bohr): 18.495012   6.302862 -2.776511
98(C) --> Charge: 6.000000 x,y,z(Bohr): 16.924880   9.890829 -1.716883
99(H) --> Charge: 1.000000 x,y,z(Bohr): 19.010741 -5.490711 -2.805549
100(C) --> Charge: 6.000000 x,y,z(Bohr): 18.048020 -9.190052 -1.405966
101(H) --> Charge: 1.000000 x,y,z(Bohr): 13.290183 -5.800777   2.993312
102(C) --> Charge: 6.000000 x,y,z(Bohr): 14.824824 -9.354217   1.769465
103(H) --> Charge: 1.000000 x,y,z(Bohr): 11.839792 10.728063   2.107512
104(C) --> Charge: 6.000000 x,y,z(Bohr): 15.063788 11.238031 -0.372984

105(H) --> Charge: 1.000000 x,y,z(Bohr): 18.313558 10.910402 -2.852832
106(H) --> Charge: 1.000000 x,y,z(Bohr): 19.412184 -10.086609 -2.660968
107(C) --> Charge: 6.000000 x,y,z(Bohr): 16.545813 -10.659107 0.214883
108(H) --> Charge: 1.000000 x,y,z(Bohr): 13.626859 -10.400608 3.083378
109(C) --> Charge: 6.000000 x,y,z(Bohr): 14.997177 14.141456 -0.491214
110(C) --> Charge: 6.000000 x,y,z(Bohr): 16.710270 -13.558384 0.341616
111(C) --> Charge: 6.000000 x,y,z(Bohr): 14.621992 14.986327 -3.262681
112(C) --> Charge: 6.000000 x,y,z(Bohr): 17.524884 15.207822 0.512525
113(C) --> Charge: 6.000000 x,y,z(Bohr): 12.838418 15.262436 1.102470
114(H) --> Charge: 1.000000 x,y,z(Bohr): 12.828069 14.250996 -4.023409
115(H) --> Charge: 1.000000 x,y,z(Bohr): 16.161741 14.315911 -4.490532
116(H) --> Charge: 1.000000 x,y,z(Bohr): 14.574386 17.066159 -3.383223
117(H) --> Charge: 1.000000 x,y,z(Bohr): 19.145043 14.540773 -0.609031
118(H) --> Charge: 1.000000 x,y,z(Bohr): 17.512525 17.290077 0.432571
119(H) --> Charge: 1.000000 x,y,z(Bohr): 17.835372 14.636232 2.491072
120(H) --> Charge: 1.000000 x,y,z(Bohr): 12.869542 17.338810 0.960402
121(H) --> Charge: 1.000000 x,y,z(Bohr): 10.973839 14.614059 0.442543
122(H) --> Charge: 1.000000 x,y,z(Bohr): 13.025824 14.765699 3.115164
123(C) --> Charge: 6.000000 x,y,z(Bohr): 17.425856 -14.368588 3.055664
124(C) --> Charge: 6.000000 x,y,z(Bohr): 14.113941 -14.700335 -0.358673
125(C) --> Charge: 6.000000 x,y,z(Bohr): 18.702140 -14.631753 -1.484899
126(H) --> Charge: 1.000000 x,y,z(Bohr): 19.273463 -13.577609 3.601401
127(H) --> Charge: 1.000000 x,y,z(Bohr): 16.018979 -13.733481 4.450283
128(H) --> Charge: 1.000000 x,y,z(Bohr): 17.546502 -16.444998 3.178775
129(H) --> Charge: 1.000000 x,y,z(Bohr): 13.561314 -14.152636 -2.290539
130(H) --> Charge: 1.000000 x,y,z(Bohr): 12.616025 -14.069373 0.939913
131(H) --> Charge: 1.000000 x,y,z(Bohr): 14.194157 -16.780599 -0.271439
132(H) --> Charge: 1.000000 x,y,z(Bohr): 18.750724 -16.706254 -1.324949
133(H) --> Charge: 1.000000 x,y,z(Bohr): 18.266969 -14.163598 -3.465814
134(H) --> Charge: 1.000000 x,y,z(Bohr): 20.610307 -13.922123 -1.051215
135(H) --> Charge: 1.000000 x,y,z(Bohr): 0.650504 4.918953 -3.831638
136(C) --> Charge: 6.000000 x,y,z(Bohr): -0.150541 5.482959 -5.670326
137(H) --> Charge: 1.000000 x,y,z(Bohr): 1.415356 5.973730 -6.952447
138(O) --> Charge: 8.000000 x,y,z(Bohr): -1.649378 7.722753 -5.442003
139(C) --> Charge: 6.000000 x,y,z(Bohr): -1.656540 3.324436 -6.909115
140(C) --> Charge: 6.000000 x,y,z(Bohr): -3.345535 7.780370 -3.353190
141(H) --> Charge: 1.000000 x,y,z(Bohr): -2.950520 2.425467 -5.535409
142(H) --> Charge: 1.000000 x,y,z(Bohr): -2.817065 4.149526 -8.428027
143(O) --> Charge: 8.000000 x,y,z(Bohr): -0.066250 1.498455 -8.093471
144(H) --> Charge: 1.000000 x,y,z(Bohr): -4.000816 5.867754 -2.857435
145(H) --> Charge: 1.000000 x,y,z(Bohr): -5.013187 8.875347 -3.957948
146(C) --> Charge: 6.000000 x,y,z(Bohr): -2.241345 9.068646 -1.033388
147(C) --> Charge: 6.000000 x,y,z(Bohr): 1.021838 -0.335517 -6.445642
148(H) --> Charge: 1.000000 x,y,z(Bohr): -1.617438 11.005116 -1.533422
149(H) --> Charge: 1.000000 x,y,z(Bohr): -3.744812 9.232450 0.418645
150(O) --> Charge: 8.000000 x,y,z(Bohr): -0.153758 7.641798 -0.127202
151(H) --> Charge: 1.000000 x,y,z(Bohr): 1.532531 0.490960 -4.609678
152(H) --> Charge: 1.000000 x,y,z(Bohr): 2.776697 -0.992749 -7.353492
153(C) --> Charge: 6.000000 x,y,z(Bohr): -0.733722 -2.594510 -6.072756
154(C) --> Charge: 6.000000 x,y,z(Bohr): 0.876787 8.518858 2.198576
155(H) --> Charge: 1.000000 x,y,z(Bohr): -2.493039 -2.010992 -5.100988
156(H) --> Charge: 1.000000 x,y,z(Bohr): -1.273292 -3.361639 -7.948546
157(O) --> Charge: 8.000000 x,y,z(Bohr): 0.579206 -4.429946 -4.625362
158(H) --> Charge: 1.000000 x,y,z(Bohr): 0.576375 10.576196 2.404148
159(H) --> Charge: 1.000000 x,y,z(Bohr): 2.927215 8.163251 2.126297
160(C) --> Charge: 6.000000 x,y,z(Bohr): -0.188469 7.163310 4.501849
161(C) --> Charge: 6.000000 x,y,z(Bohr): -0.847943 -6.671561 -4.270215
162(H) --> Charge: 1.000000 x,y,z(Bohr): 0.346731 8.237555 6.222529
163(H) --> Charge: 1.000000 x,y,z(Bohr): -2.283550 7.109812 4.421164
164(O) --> Charge: 8.000000 x,y,z(Bohr): 0.837190 4.688893 4.597314
165(H) --> Charge: 1.000000 x,y,z(Bohr): -2.611924 -6.258756 -3.230711
166(H) --> Charge: 1.000000 x,y,z(Bohr): -1.384552 -7.477961 -6.132171
167(C) --> Charge: 6.000000 x,y,z(Bohr): 0.722172 -8.644195 -2.881851
168(C) --> Charge: 6.000000 x,y,z(Bohr): 0.341664 3.396489 6.895036
169(H) --> Charge: 1.000000 x,y,z(Bohr): 2.628728 -8.670589 -3.717477

170(H) --> Charge: 1.000000 x,y,z(Bohr): -0.156947 -10.519036 -3.202784
171(O) --> Charge: 8.000000 x,y,z(Bohr): 1.080099 -8.249505 -0.248089
172(H) --> Charge: 1.000000 x,y,z(Bohr): -1.702269 2.983239 7.109737
173(H) --> Charge: 1.000000 x,y,z(Bohr): 0.917830 4.594145 8.517580
174(C) --> Charge: 6.000000 x,y,z(Bohr): 1.859644 0.944698 6.935697
175(C) --> Charge: 6.000000 x,y,z(Bohr): -1.073103 -8.781773 1.271869
176(H) --> Charge: 1.000000 x,y,z(Bohr): 3.650546 1.287372 5.927966
177(H) --> Charge: 1.000000 x,y,z(Bohr): 2.312209 0.444783 8.913197
178(O) --> Charge: 8.000000 x,y,z(Bohr): 0.643391 -1.115771 5.712811
179(H) --> Charge: 1.000000 x,y,z(Bohr): -2.552586 -7.338915 0.995120
180(H) --> Charge: 1.000000 x,y,z(Bohr): -1.873048 -10.650830 0.756588
181(C) --> Charge: 6.000000 x,y,z(Bohr): -0.259576 -8.880590 4.031251
182(C) --> Charge: 6.000000 x,y,z(Bohr): -0.679210 -2.755355 7.371544
183(H) --> Charge: 1.000000 x,y,z(Bohr): -1.794742 -9.765962 5.148218
184(H) --> Charge: 1.000000 x,y,z(Bohr): 1.428364 -10.091989 4.171742
185(O) --> Charge: 8.000000 x,y,z(Bohr): 0.432625 -6.515373 5.099895
186(H) --> Charge: 1.000000 x,y,z(Bohr): -2.311917 -1.786361 8.259746
187(H) --> Charge: 1.000000 x,y,z(Bohr): 0.587649 -3.412003 8.905965
188(C) --> Charge: 6.000000 x,y,z(Bohr): -1.650890 -5.021228 5.917592
189(H) --> Charge: 1.000000 x,y,z(Bohr): -2.883783 -6.138997 7.192247
190(H) --> Charge: 1.000000 x,y,z(Bohr): -2.792077 -4.376935 4.300099

Note: Orbital 341 is HOMO, energy: -0.206005 a.u. -5.605668 eV
Orbital 342 is LUMO, energy: -0.080277 a.u. -2.184451 eV
HOMO-LUMO gap: 0.125727 a.u. 3.421217 eV 330.097294 kJ/mol

Frequencies in normal mode approximation

Frequency calculations within normal mode approximation were carried out to characterize, as local minimum, the optimized geometries derived within the applied C-PCM/DFT computational scenario. More specifically, the optimized geometry of Stop-Bzi-Bipy(24C8)-Bzi-Stop and Stop-Bzi(24C8)-Bipy($PtCl_2$)-Bzi-Stop supramolecular aggregates are then considered as starting coordinates to estimate the associated normal modes at C-PCM/B3LYP(D3)/cc-pVTZ level of theory. In this respect, we would like to mention that all the computed frequencies feature values grater than zero within the applied approximations. In addition, we also investigated a model system composed by Bzi-Bipy($PtCl_2$)-Bzi wire with the basic idea of detecting the bending and stretching movements involving $Pt^{(II)}Cl_2$ chemical moiety. Furthermore, for the sake of completeness, we report below some of the normal modes characterized with particular emphasis on the normal modes of $PtCl_2$ and on the stretching motion of the N-H chemical group of the Bzi recognition site. The latter to analyze the effect of hydrogen bonding contacts with the ether oxygen atoms of the macrocycle over the associated frequency. At last, we also explicitly report a comparison between the computed IR spectra of Stop-Bzi-Bipy(24C8)-Bzi-Stop and Stop-Bzi(24C8)-Bipy($PtCl_2$)-Bzi-Stop systems highlighting the shift to lower frequencies observed for N-H stretching downstream of complexation.

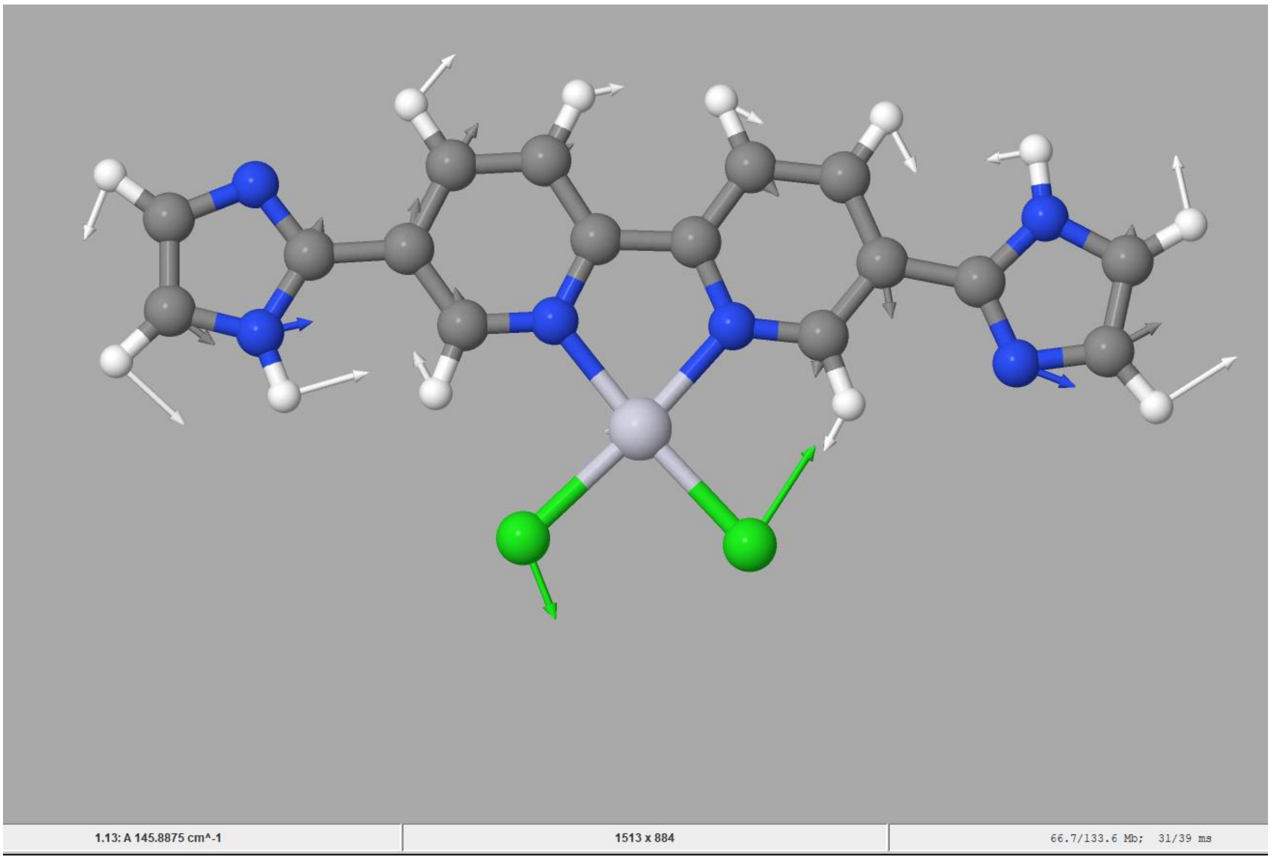
1.13: A 145.8875 cm^-1
1513 x 884
66.7/133.6 Mb; 31/39 ms

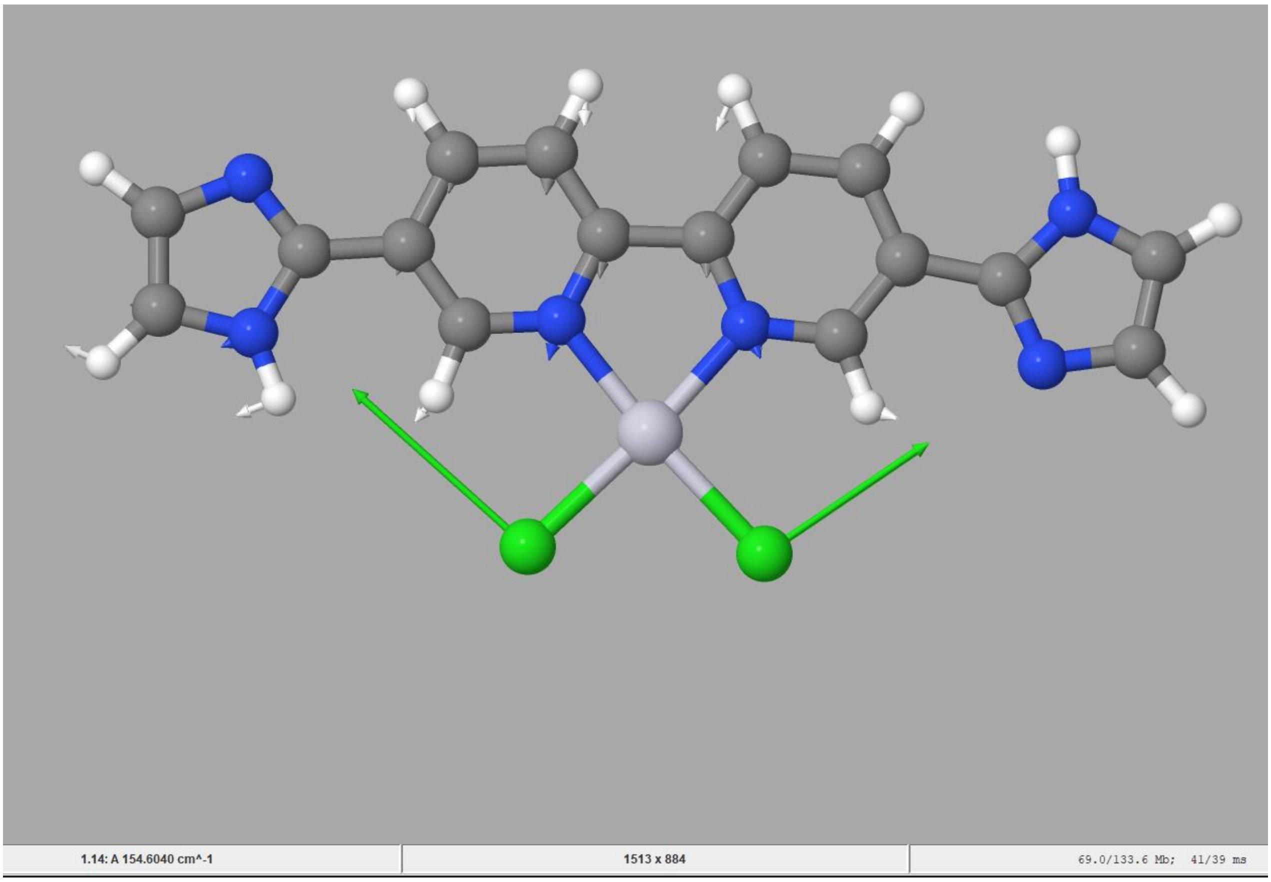
1.14: A 154.6040 cm^-1
1513 x 884
69.0/133.6 Mb; 41/39 ms

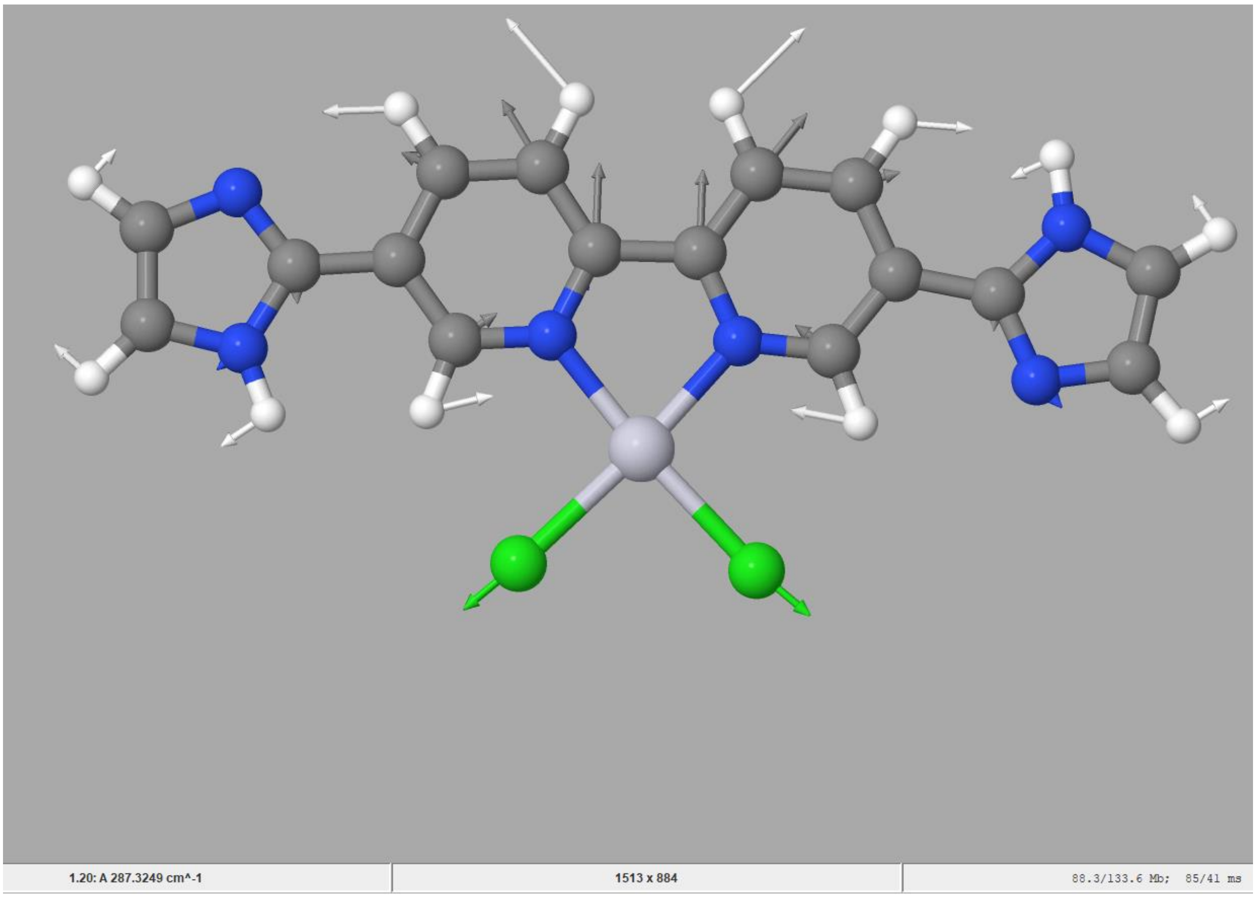
1.20: A 287.3249 cm^-1
1513 x 884
88.3/133.6 Mb; 85/41 ms

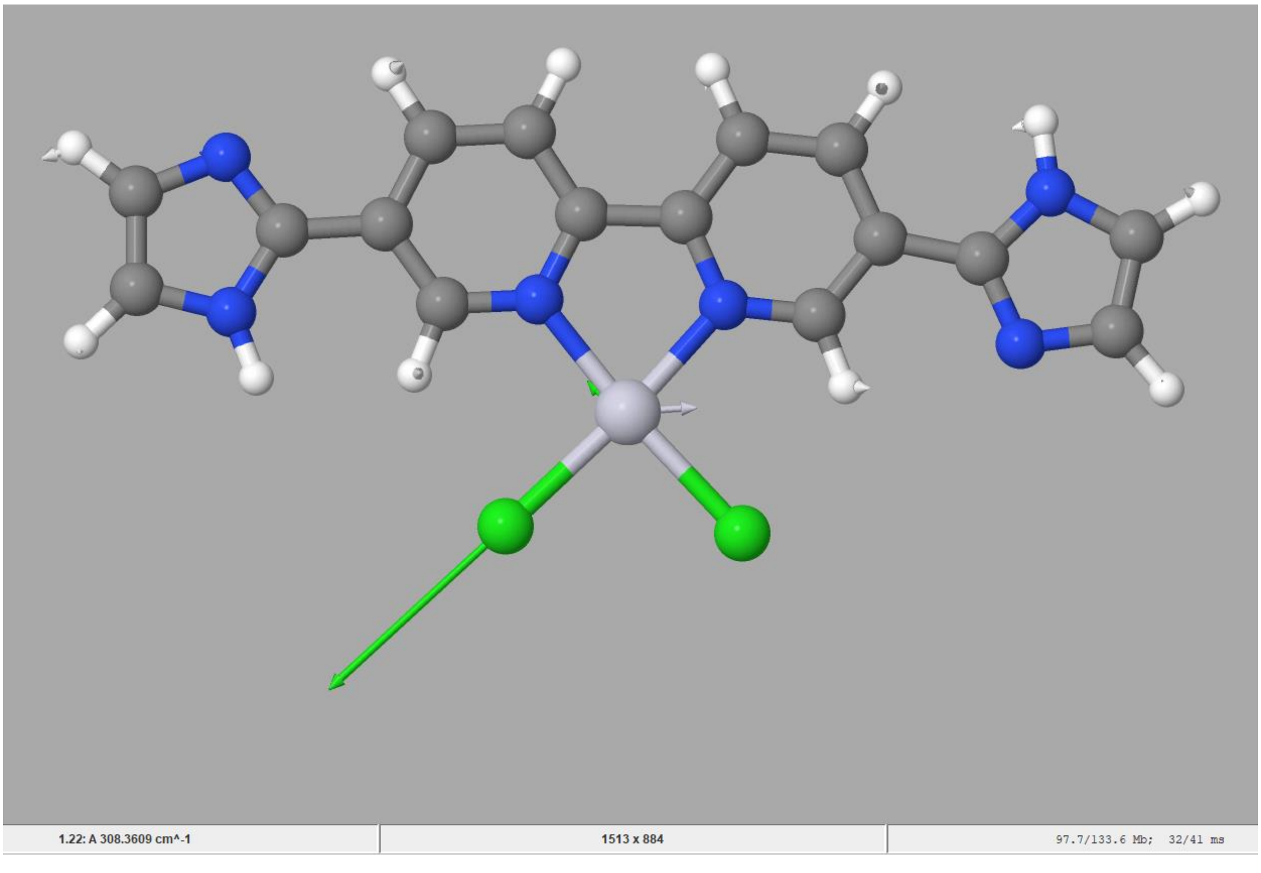
1.22: A 308.3609 cm^-1
1513 x 884
97.7/133.6 Mb; 32/41 ms

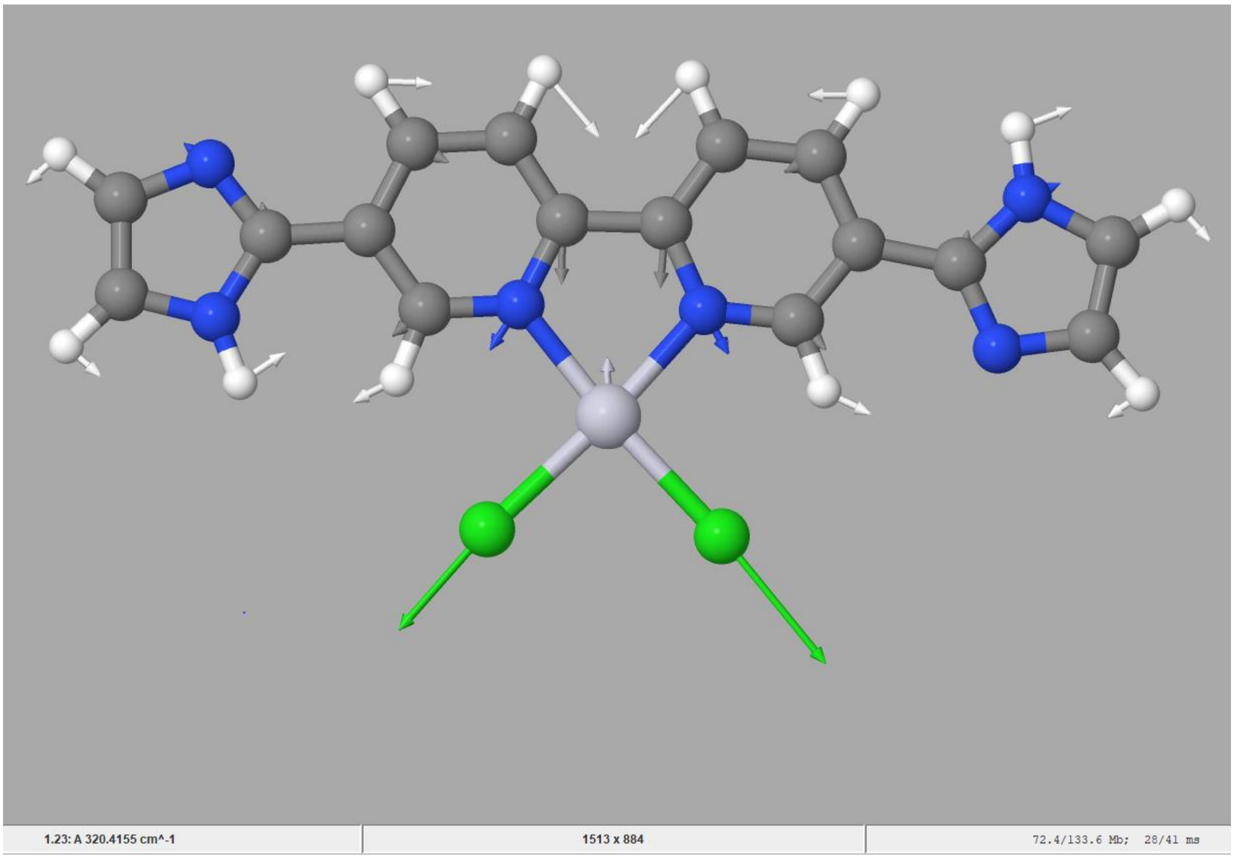
1.23: A 320.4155 cm^-1
1513 x 884
72.4/133.6 Mb; 28/41 ms

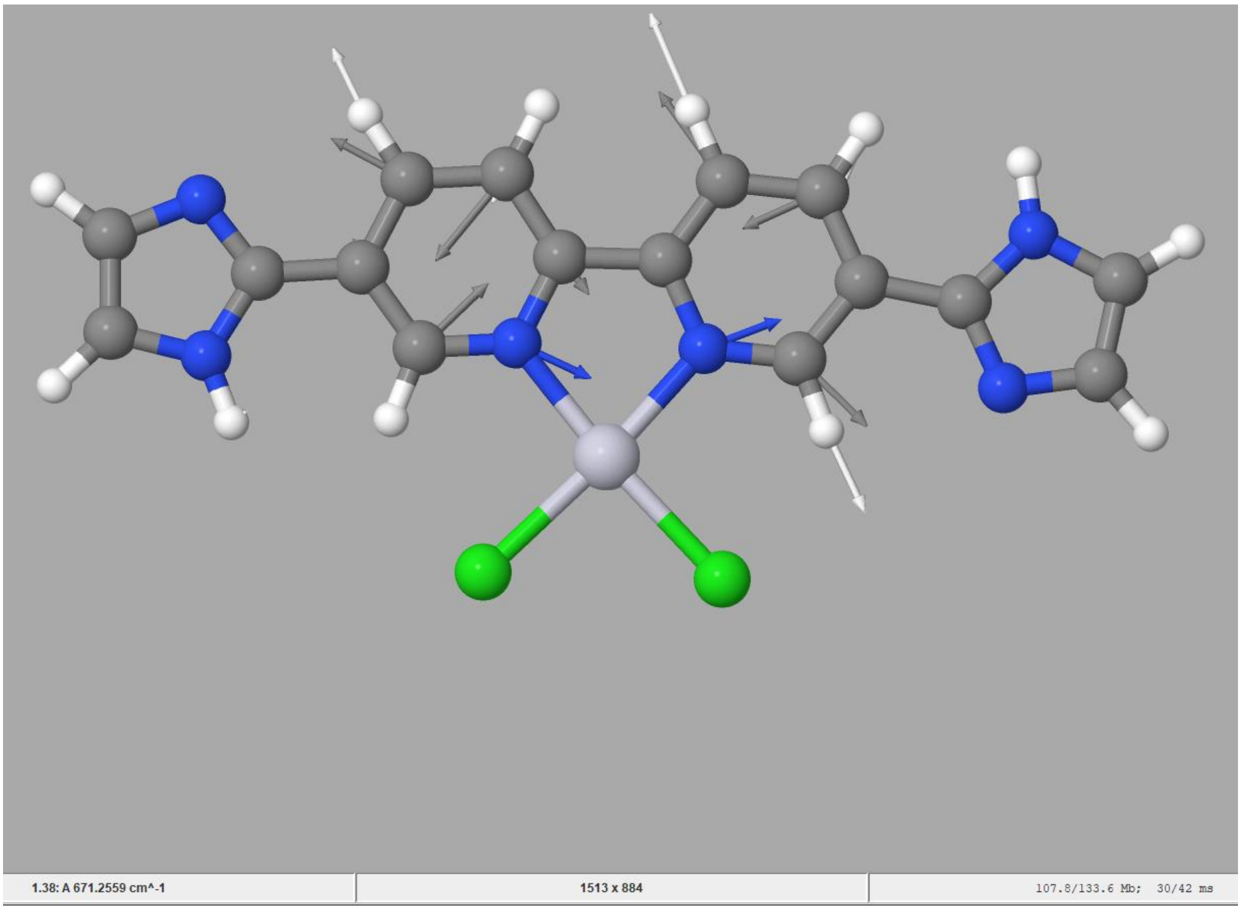
1.38: A 671.2559 cm^-1
1513 x 884
107.8/133.6 Mb; 30/42 ms

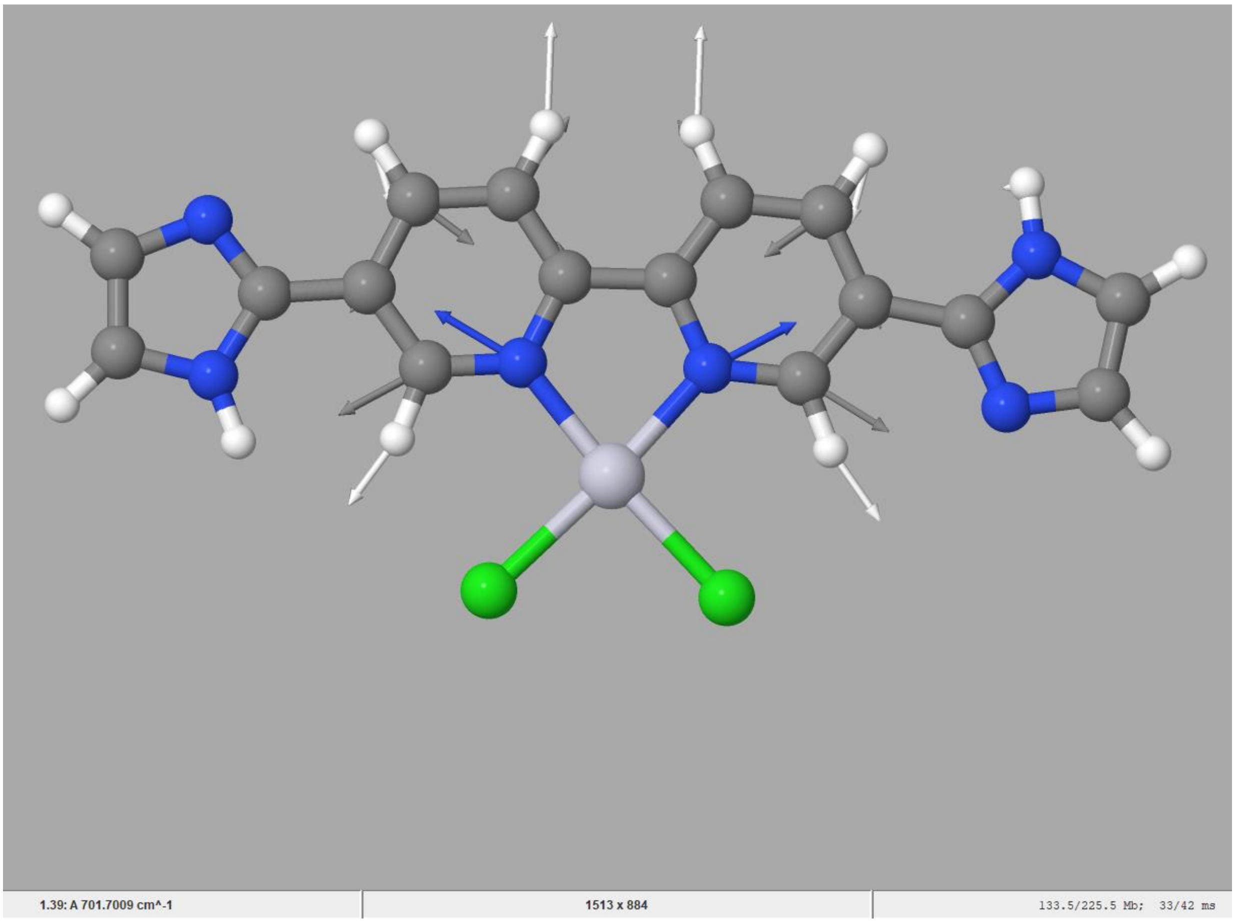
1.39: A 701.7009 cm^-1
1513 x 884
133.5/225.5 Mb; 33/42 ms

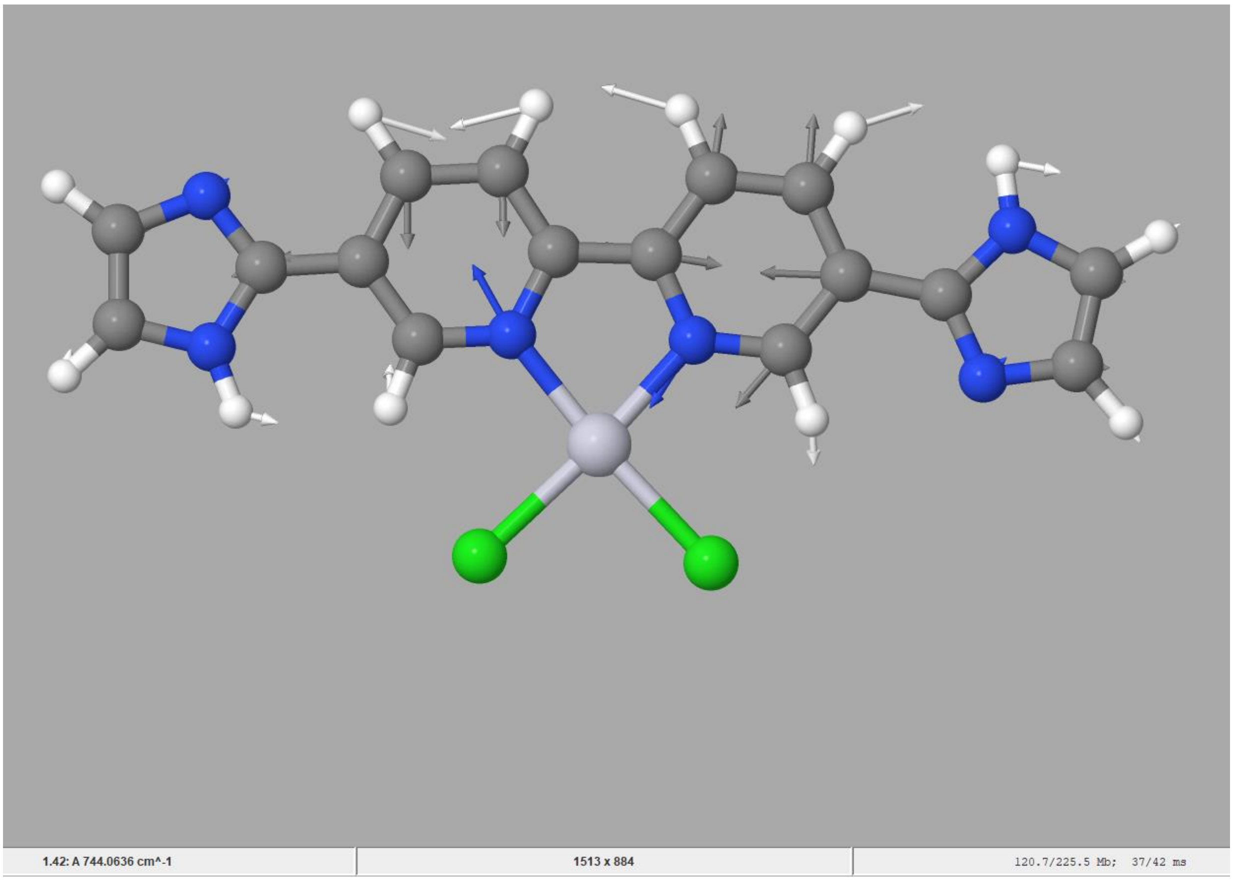
1.42: A 744.0636 cm^-1
1513 x 884
120.7/225.5 Mb; 37/42 ms

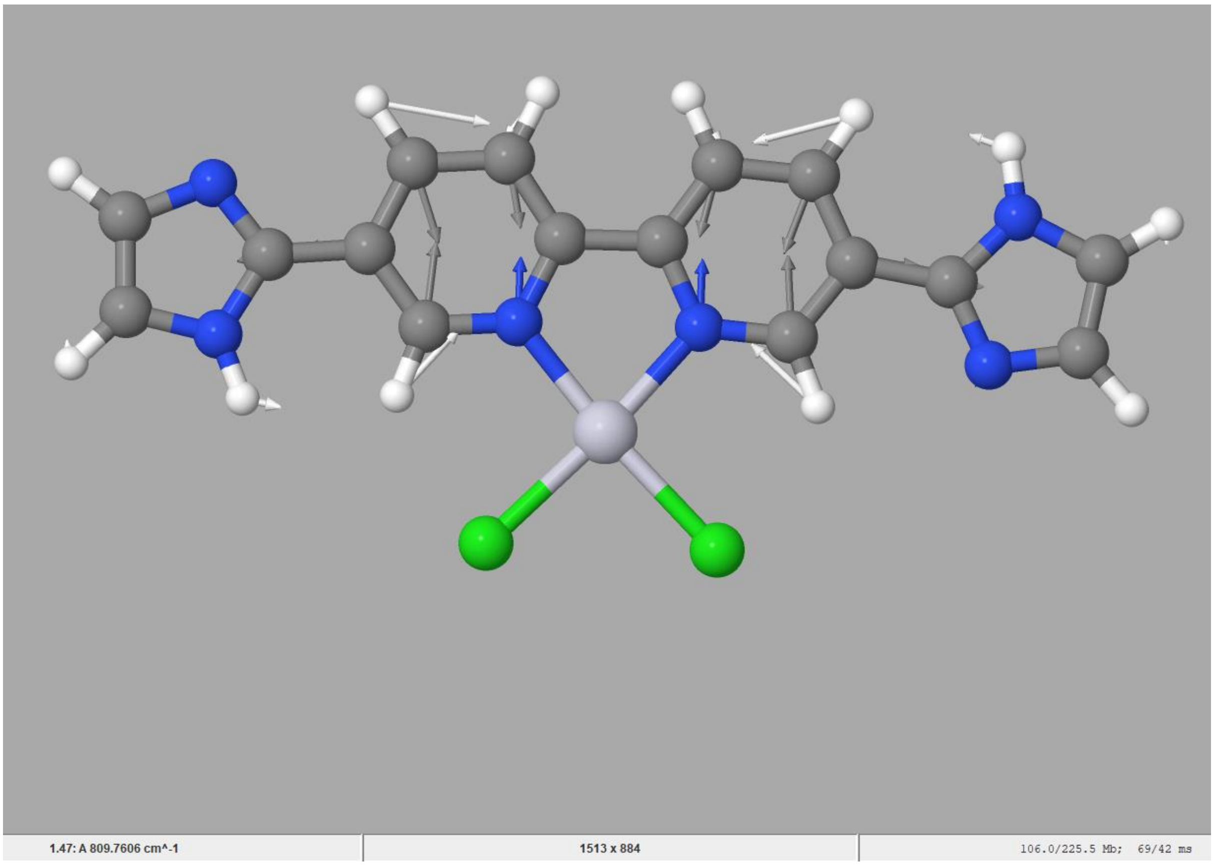
1.47: A 809.7606 cm^-1
1513 x 884
106.0/225.5 Mb; 69/42 ms

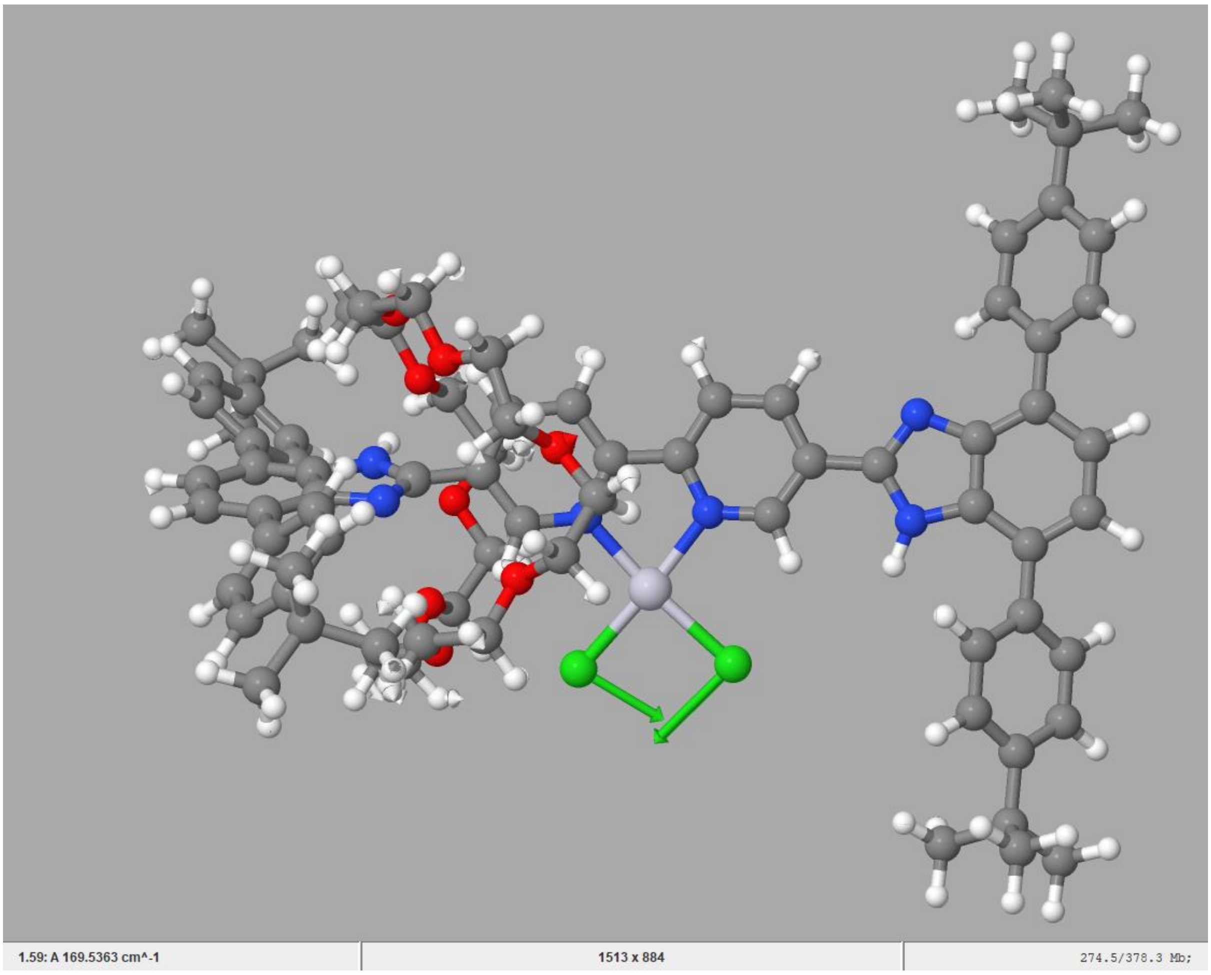
1.59: A 169.5363 cm^-1
1513 x 884
274.5/378.3 Mb;

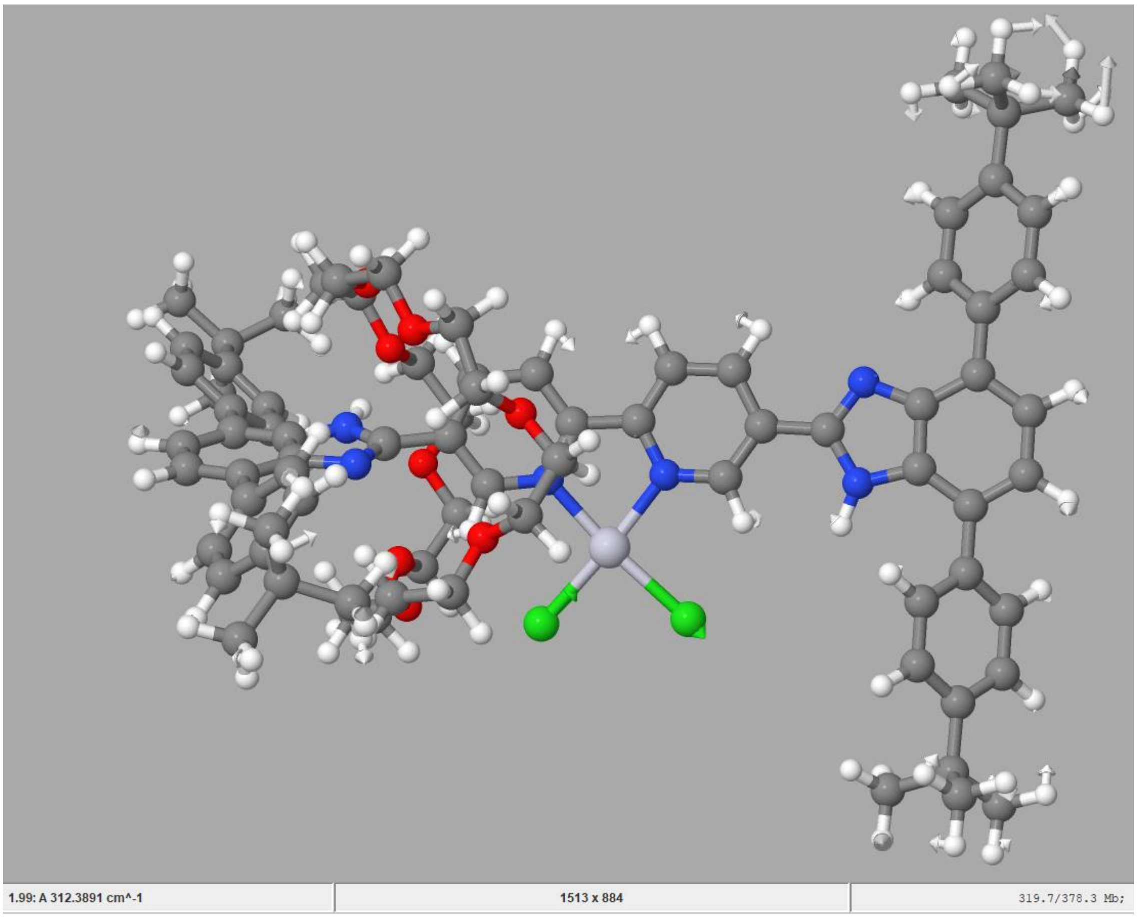
1.99: A 312.3891 cm^-1
1513 x 884
319.7/378.3 Mb;

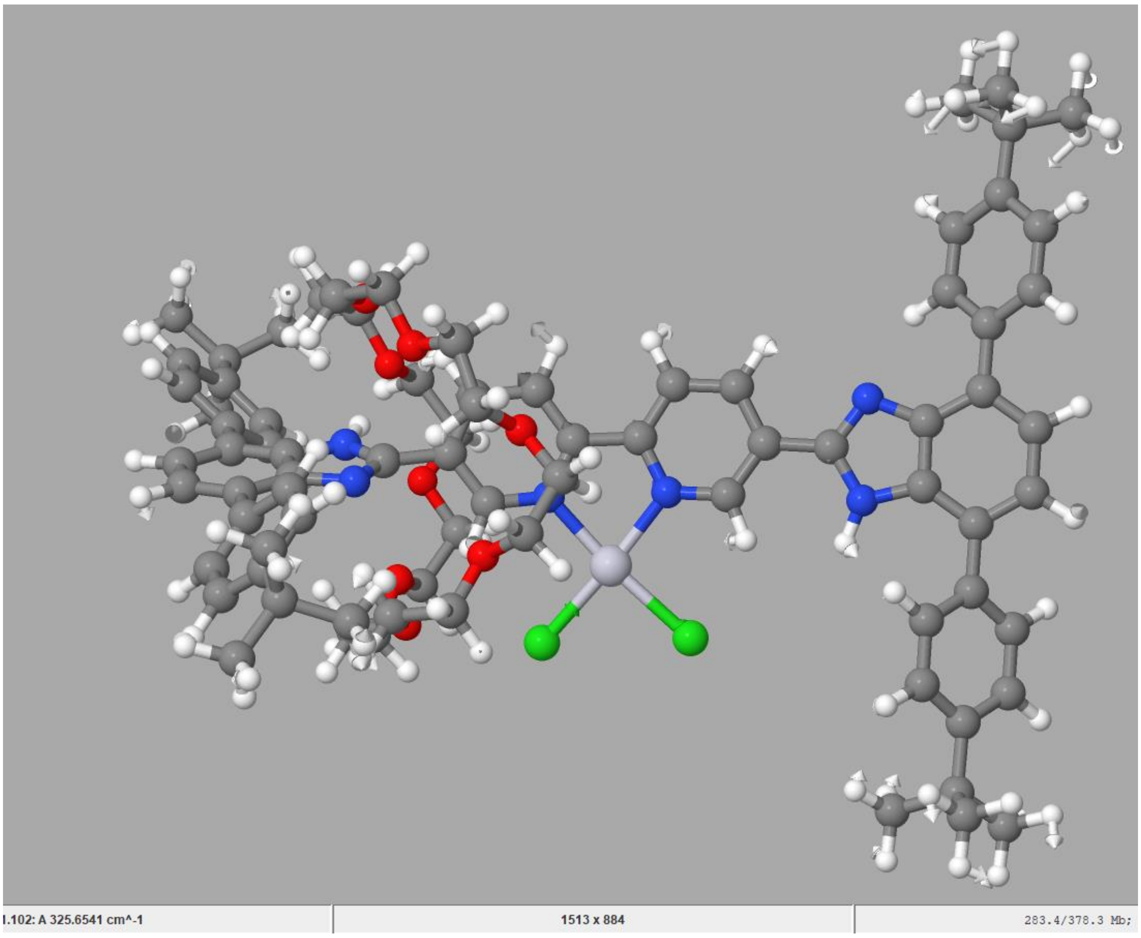
.102: A 325.6541 cm^-1
1513 x 884
283.4/378.3 Mb;

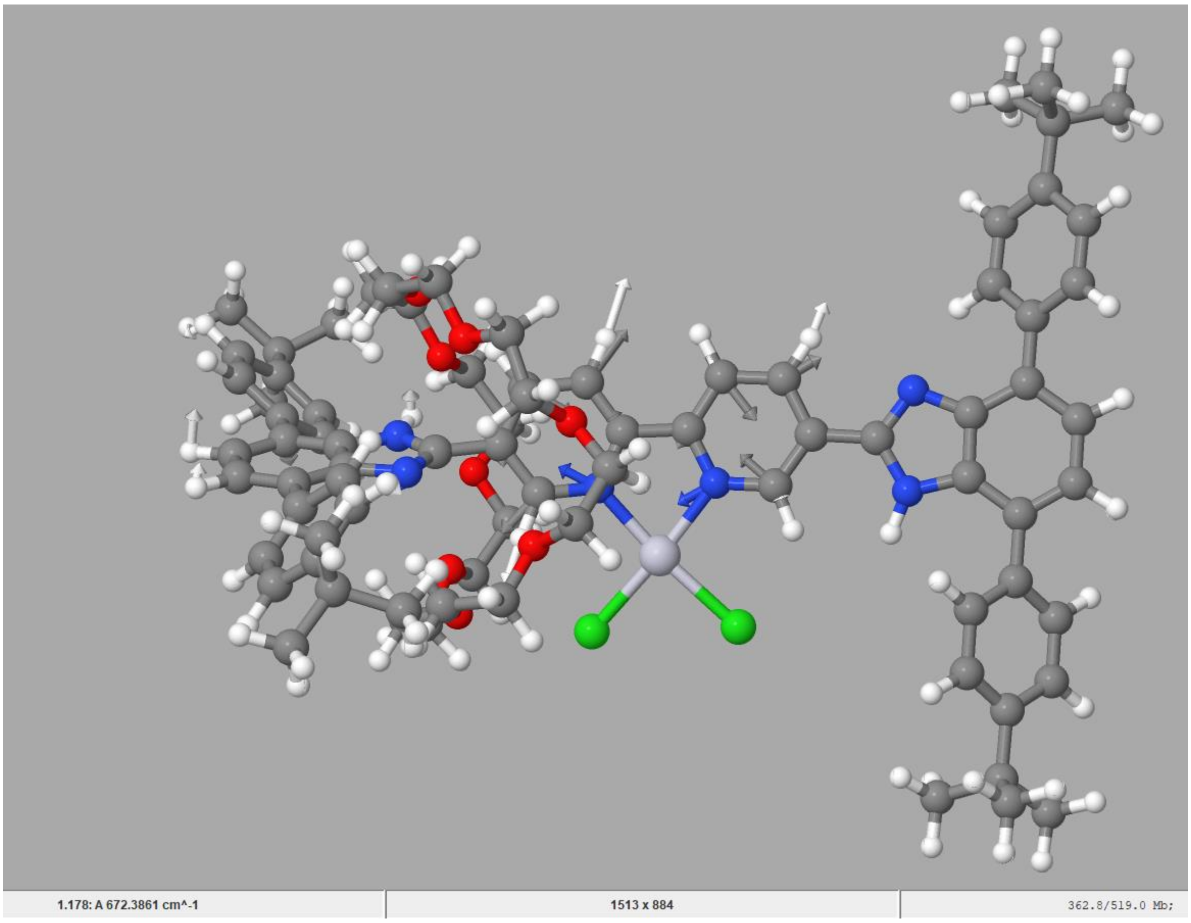
1.178: A 672.3861 cm^-1
1513 x 884
362.8/519.0 Mb;

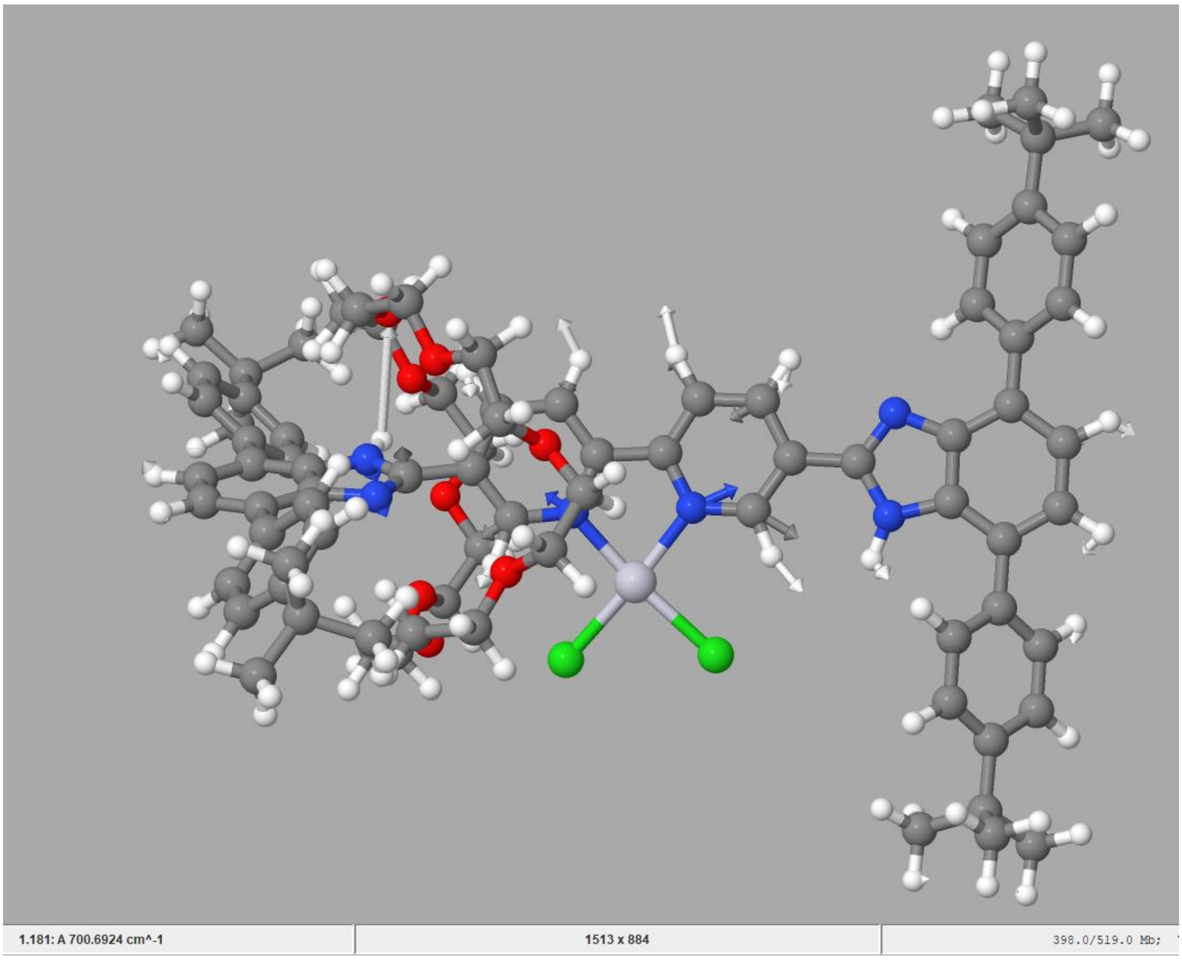
1.181: A 700.6924 cm^-1
1513 x 884
398.0/519.0 Mb;

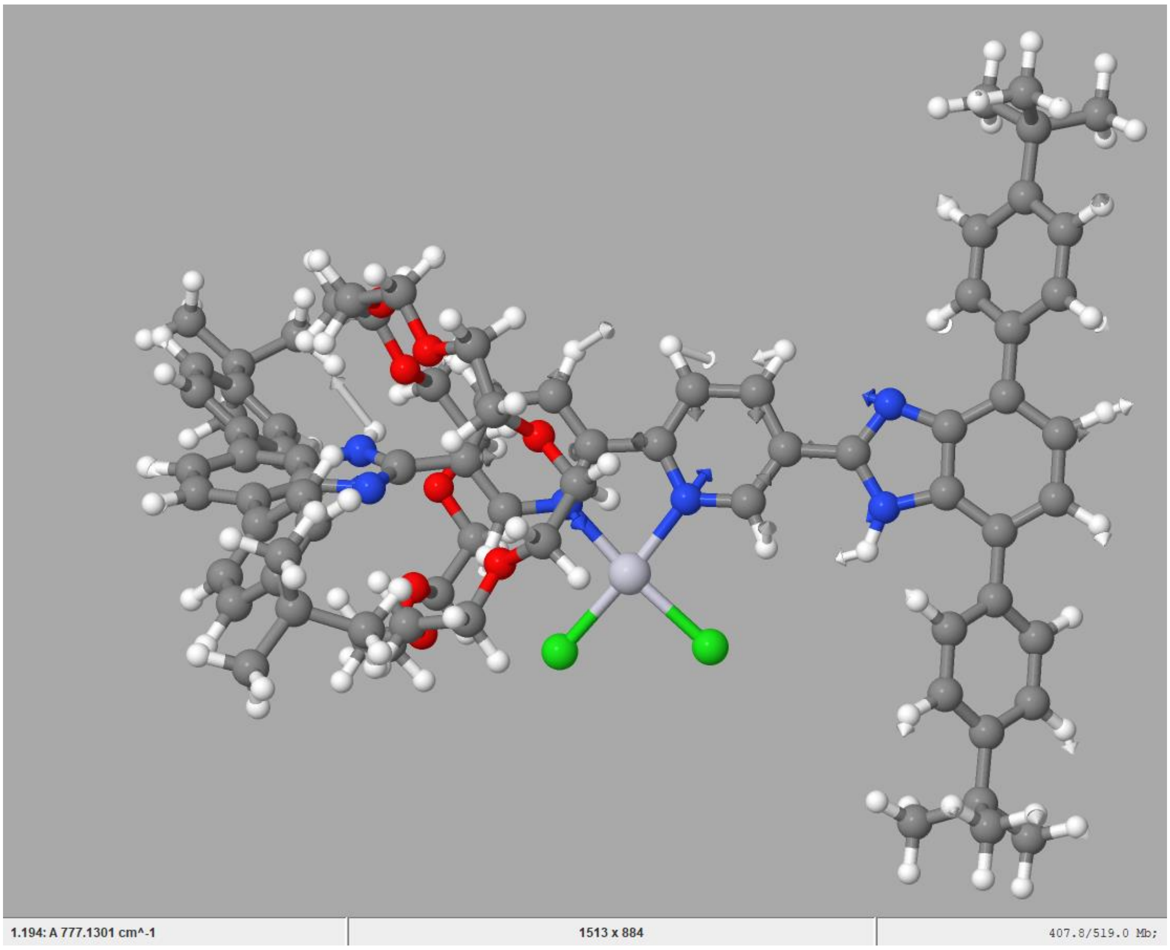
1.194: A 777.1301 cm^-1
1513 x 884
407.8/519.0 Mb;

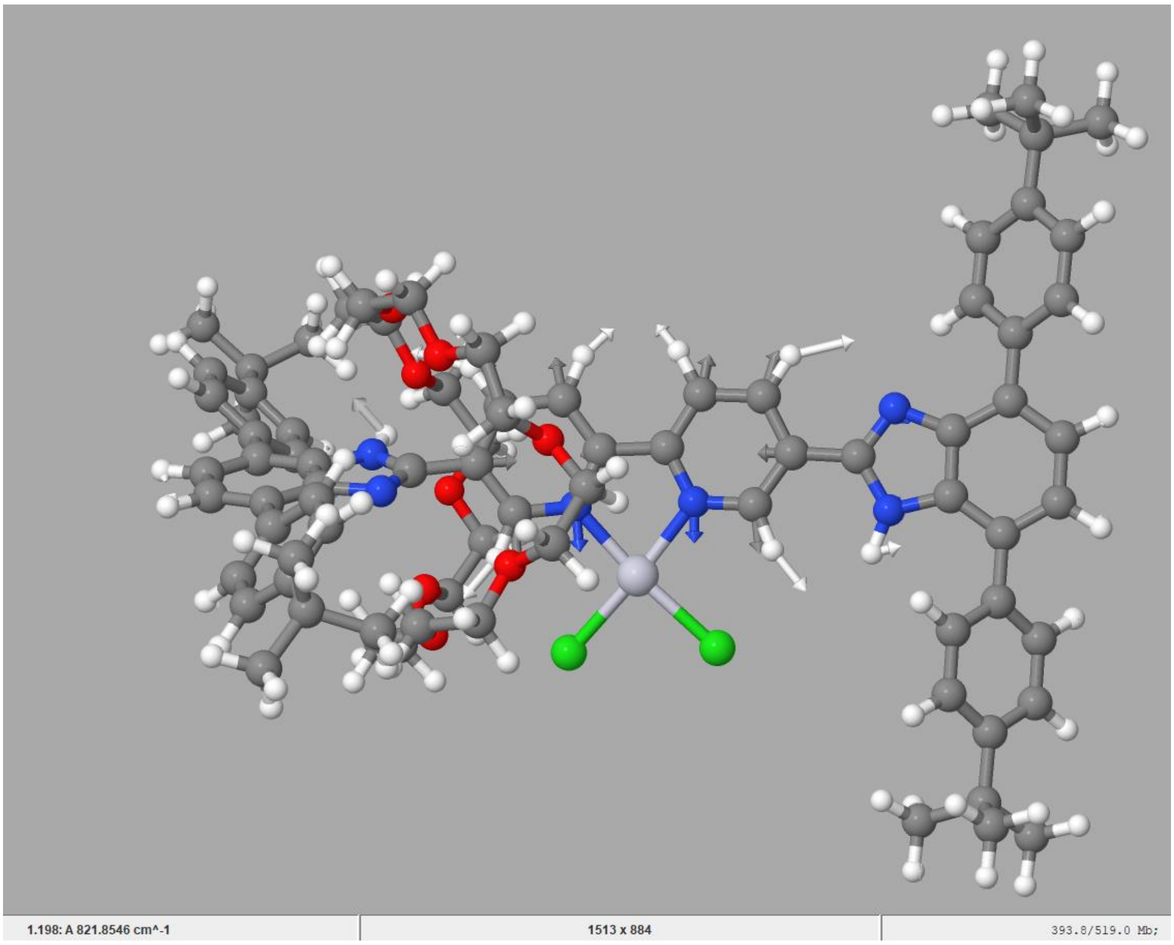
1.198: A 821.8546 cm^-1
1513 x 884
393.8/519.0 Mb;

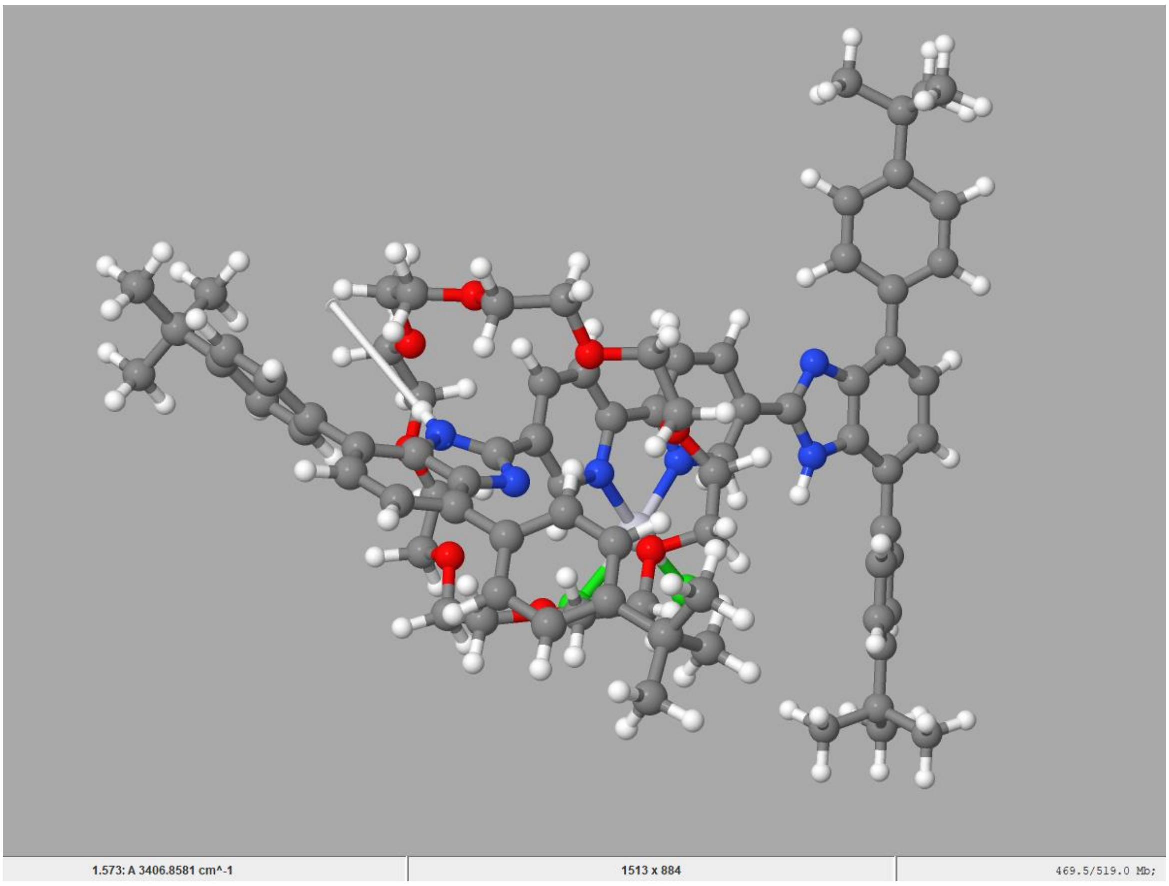
1.573: A 3406.8581 cm^-1
1513 x 884
469.5/519.0 Mb;

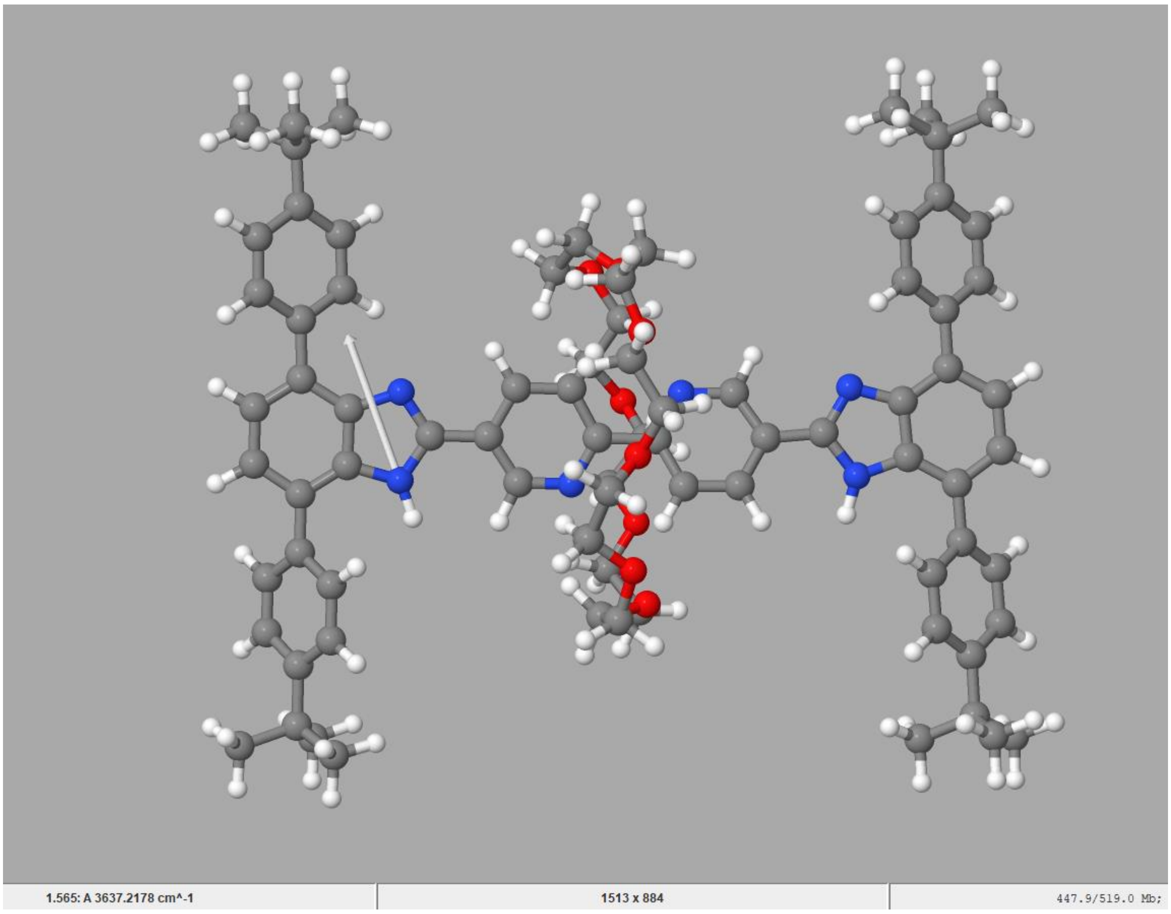
1.565: A 3637.2178 cm^-1
1513 x 884
447.9/519.0 Mb;

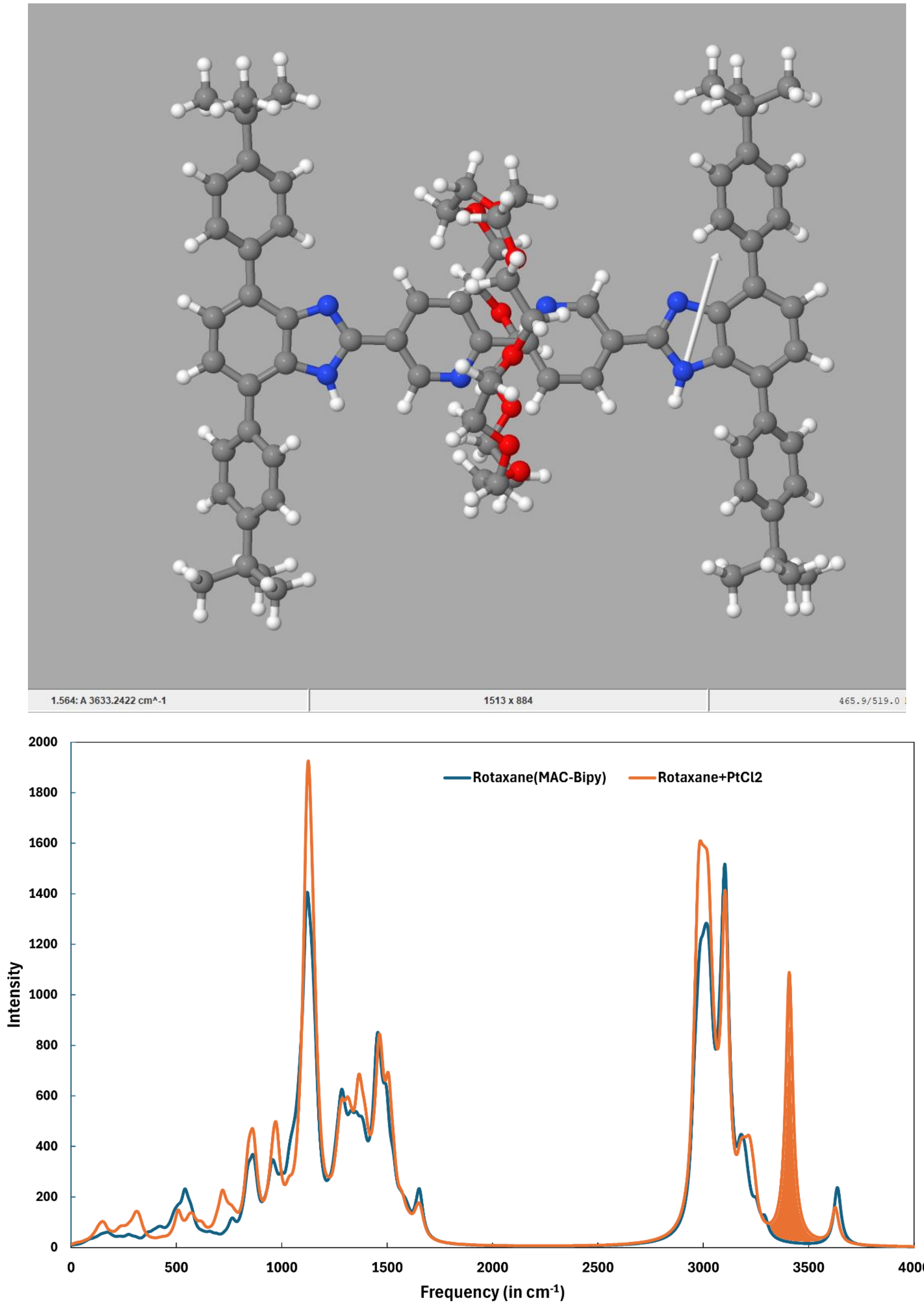

Computed IR absorption spectra - at C-PCM/B3LYP(D3)/cc-pVTZ level of theory - for the investigated Stop-[Bzi-Bipy(24C8)-Bzi]-Stop [Rotaxane(MAC-Bipy)] and Stop-[Bzi(24C8)-Bipy($PtCl_2$)-Bzi]-Stop (Rotaxane+$PtCl_2$) systems. Please note that the observed blue shift (from ~ 3410 to ~3630 cm^{-1}) following the complexation of the N-H chemical group of the Bzi station is highlighed.

MD section: topology files used for the Stop-[Bzi-Bipy-Bzi]-Stop [2] molecular thread (TRD residue) and 24C8 macrocycle (MAC residue), respectively:

;
; GENERATED BY LigParGen Server
; Jorgensen Lab @ Yale University
;
[moleculetype]
; Name nrexcl
TRD 3
[atoms]

; nr	type	resnr	residue	atom	cgnr	charge	mass
1	opls_800	1	TRD	H00	1	0.1831	1.0080
2	opls_801	1	TRD	C01	1	-0.1571	12.0110
3	opls_802	1	TRD	C02	1	-0.0504	12.0110
4	opls_803	1	TRD	C03	1	0.1269	12.0110
5	opls_804	1	TRD	H04	1	0.1797	1.0080
6	opls_805	1	TRD	C05	1	-0.0707	12.0110
7	opls_806	1	TRD	N06	1	-0.4186	14.0070
8	opls_807	1	TRD	C07	1	0.1213	12.0110
9	opls_808	1	TRD	C08	1	0.1011	12.0110
10	opls_809	1	TRD	C09	1	0.2648	12.0110
11	opls_810	1	TRD	N0A	1	-0.4182	14.0070
12	opls_811	1	TRD	C0B	1	-0.1487	12.0110
13	opls_812	1	TRD	H0C	1	0.1668	1.0080
14	opls_813	1	TRD	N0D	1	-0.3371	14.0070
15	opls_814	1	TRD	N0E	1	-0.6355	14.0070
16	opls_815	1	TRD	C0F	1	0.1057	12.0110
17	opls_816	1	TRD	H0G	1	0.1869	1.0080
18	opls_817	1	TRD	C0H	1	-0.0493	12.0110
19	opls_818	1	TRD	C0I	1	0.0044	12.0110
20	opls_819	1	TRD	H0J	1	0.4699	1.0080
21	opls_820	1	TRD	C0K	1	0.0888	12.0110
22	opls_821	1	TRD	H0M	1	0.1787	1.0080
23	opls_822	1	TRD	C0N	1	-0.0732	12.0110
24	opls_823	1	TRD	H0O	1	0.1765	1.0080
25	opls_824	1	TRD	C0P	1	0.0471	12.0110
26	opls_825	1	TRD	C0Q	1	-0.0458	12.0110
27	opls_826	1	TRD	C0R	1	0.2661	12.0110
28	opls_827	1	TRD	C0S	1	-0.1566	12.0110
29	opls_828	1	TRD	C0T	1	-0.0416	12.0110
30	opls_829	1	TRD	C0U	1	-0.0977	12.0110
31	opls_830	1	TRD	C0V	1	-0.0449	12.0110
32	opls_831	1	TRD	N0W	1	-0.4064	14.0070
33	opls_832	1	TRD	N0X	2	-0.5839	14.0070
34	opls_833	1	TRD	H0Y	2	0.1455	1.0080
35	opls_834	1	TRD	C0Z	2	-0.1026	12.0110
36	opls_835	1	TRD	C10	2	-0.1236	12.0110
37	opls_836	1	TRD	H11	2	0.1475	1.0080
38	opls_837	1	TRD	C12	2	-0.1216	12.0110
39	opls_838	1	TRD	C13	2	-0.1422	12.0110
40	opls_839	1	TRD	C14	2	0.0425	12.0110
41	opls_840	1	TRD	H15	2	0.4543	1.0080
42	opls_841	1	TRD	C16	2	0.0584	12.0110
43	opls_842	1	TRD	H17	2	0.1645	1.0080
44	opls_843	1	TRD	C18	2	-0.1452	12.0110
45	opls_844	1	TRD	H19	2	0.1337	1.0080
46	opls_845	1	TRD	C1A	2	-0.144	12.0110
47	opls_846	1	TRD	H1B	2	0.1452	1.0080
48	opls_847	1	TRD	C1C	2	-0.1354	12.0110
49	opls_848	1	TRD	H1D	2	0.1435	1.0080
50	opls_849	1	TRD	C1E	2	-0.1351	12.0110

51	opls_850	1	TRD	C1F	2	0.041	12.0110
52	opls_851	1	TRD	C1G	2	-0.032	12.0110
53	opls_852	1	TRD	H1H	2	0.1388	1.0080
54	opls_853	1	TRD	C1I	2	-0.0556	12.0110
55	opls_854	1	TRD	H1J	2	0.1372	1.0080
56	opls_855	1	TRD	H1K	2	0.1423	1.0080
57	opls_856	1	TRD	C1M	2	-0.0595	12.0110
58	opls_857	1	TRD	H1N	2	0.1396	1.0080
59	opls_858	1	TRD	C1O	2	-0.1491	12.0110
60	opls_859	1	TRD	C1P	2	-0.0426	12.0110
61	opls_860	1	TRD	C1Q	2	-0.1091	12.0110
62	opls_861	1	TRD	C1R	2	-0.0415	12.0110
63	opls_862	1	TRD	C1S	2	-0.0073	12.0110
64	opls_863	1	TRD	C1T	2	-0.0036	12.0110
65	opls_864	1	TRD	H1U	2	0.1464	1.0080
66	opls_865	1	TRD	C1V	3	-0.0932	12.0110
67	opls_866	1	TRD	C1W	3	-0.1264	12.0110
68	opls_867	1	TRD	H1X	3	0.1456	1.0080
69	opls_868	1	TRD	C1Y	3	-0.1226	12.0110
70	opls_869	1	TRD	C1Z	3	-0.1437	12.0110
71	opls_870	1	TRD	C20	3	-0.2313	12.0110
72	opls_871	1	TRD	C21	3	-0.2306	12.0110
73	opls_872	1	TRD	C22	3	-0.2288	12.0110
74	opls_873	1	TRD	H23	3	0.085	1.0080
75	opls_874	1	TRD	H24	3	0.085	1.0080
76	opls_875	1	TRD	H25	3	0.085	1.0080
77	opls_876	1	TRD	H26	3	0.0853	1.0080
78	opls_877	1	TRD	H27	3	0.0853	1.0080
79	opls_878	1	TRD	H28	3	0.0853	1.0080
80	opls_879	1	TRD	H29	3	0.0853	1.0080
81	opls_880	1	TRD	H2A	3	0.0853	1.0080
82	opls_881	1	TRD	H2B	3	0.0853	1.0080
83	opls_882	1	TRD	C2C	3	-0.2328	12.0110
84	opls_883	1	TRD	C2D	3	-0.2299	12.0110
85	opls_884	1	TRD	C2E	3	-0.2329	12.0110
86	opls_885	1	TRD	H2F	3	0.0857	1.0080
87	opls_886	1	TRD	H2G	3	0.0857	1.0080
88	opls_887	1	TRD	H2H	3	0.0857	1.0080
89	opls_888	1	TRD	H2I	3	0.0877	1.0080
90	opls_889	1	TRD	H2J	3	0.0877	1.0080
91	opls_890	1	TRD	H2K	3	0.0877	1.0080
92	opls_891	1	TRD	H2M	3	0.0866	1.0080
93	opls_892	1	TRD	H2N	3	0.0866	1.0080
94	opls_893	1	TRD	H2O	3	0.0866	1.0080
95	opls_894	1	TRD	H2P	3	0.1613	1.0080
96	opls_895	1	TRD	C2Q	3	-0.1409	12.0110
97	opls_896	1	TRD	H2R	3	0.1366	1.0080
98	opls_897	1	TRD	C2S	3	-0.143	12.0110
99	opls_898	1	TRD	H2T	4	0.146	1.0080
100	opls_899	1	TRD	C2U	4	-0.1335	12.0110
101	opls_9500	1	TRD	H2V	4	0.1361	1.0080
102	opls_9501	1	TRD	C2W	4	-0.133	12.0110
103	opls_9502	1	TRD	H2X	4	0.1391	1.0080
104	opls_9503	1	TRD	C2Y	4	-0.0595	12.0110
105	opls_9504	1	TRD	H2Z	4	0.136	1.0080
106	opls_9505	1	TRD	H30	4	0.1377	1.0080
107	opls_9506	1	TRD	C31	4	-0.0613	12.0110
108	opls_9507	1	TRD	H32	4	0.1403	1.0080
109	opls_9508	1	TRD	C33	4	-0.0052	12.0110
110	opls_9509	1	TRD	C34	4	-0.0025	12.0110
111	opls_9510	1	TRD	C35	4	-0.2305	12.0110
112	opls_9511	1	TRD	C36	4	-0.2284	12.0110
113	opls_9512	1	TRD	C37	4	-0.2316	12.0110
114	opls_9513	1	TRD	H38	4	0.0847	1.0080
115	opls_9514	1	TRD	H39	4	0.0847	1.0080

116	opls_9515	1	TRD	H3A	4	0.0847	1.0080
117	opls_9516	1	TRD	H3B	4	0.0853	1.0080
118	opls_9517	1	TRD	H3C	4	0.0853	1.0080
119	opls_9518	1	TRD	H3D	4	0.0853	1.0080
120	opls_9519	1	TRD	H3E	4	0.0857	1.0080
121	opls_9520	1	TRD	H3F	4	0.0857	1.0080
122	opls_9521	1	TRD	H3G	4	0.0857	1.0080
123	opls_9522	1	TRD	C3H	4	-0.235	12.0110
124	opls_9523	1	TRD	C3I	4	-0.2307	12.0110
125	opls_9524	1	TRD	C3J	4	-0.2285	12.0110
126	opls_9525	1	TRD	H3K	4	0.0863	1.0080
127	opls_9526	1	TRD	H3M	4	0.0863	1.0080
128	opls_9527	1	TRD	H3N	4	0.0863	1.0080
129	opls_9528	1	TRD	H3O	4	0.0867	1.0080
130	opls_9529	1	TRD	H3P	4	0.0867	1.0080
131	opls_9530	1	TRD	H3Q	4	0.0867	1.0080
132	opls_9531	1	TRD	H3R	5	0.0871	1.0080
133	opls_9532	1	TRD	H3S	5	0.0871	1.0080
134	opls_9533	1	TRD	H3T	5	0.0871	1.0080

[bonds]

2	1	1	0.1080	307105.600
3	2	1	0.1400	392459.200
4	2	1	0.1400	392459.200
5	3	1	0.1080	307105.600
6	3	1	0.1400	392459.200
7	4	1	0.1339	404174.400
8	4	1	0.1460	322168.000
9	6	1	0.1400	392459.200
10	6	1	0.1460	322168.000
11	8	1	0.1339	404174.400
12	8	1	0.1400	392459.200
13	9	1	0.1080	307105.600
14	10	1	0.1335	408358.400
15	10	1	0.1343	399153.600
16	11	1	0.1339	404174.400
17	12	1	0.1080	307105.600
18	12	1	0.1400	392459.200
19	14	1	0.1394	343088.000
20	15	1	0.1010	363171.200
21	15	1	0.1374	364844.800
22	16	1	0.1080	307105.600
23	16	1	0.1400	392459.200
24	18	1	0.1080	307105.600
25	19	1	0.1460	322168.000
26	21	1	0.1400	392459.200
27	23	1	0.1460	322168.000
28	25	1	0.1400	392459.200
29	25	1	0.1460	322168.000
30	26	1	0.1400	392459.200
31	26	1	0.1460	322168.000
32	27	1	0.1335	408358.400
33	27	1	0.1343	399153.600
34	28	1	0.1080	307105.600
35	29	1	0.1400	392459.200
36	29	1	0.1400	392459.200
37	30	1	0.1080	307105.600
38	31	1	0.1400	392459.200
39	31	1	0.1400	392459.200
40	32	1	0.1394	343088.000
41	33	1	0.1010	363171.200
42	33	1	0.1374	364844.800
43	35	1	0.1080	307105.600
44	35	1	0.1400	392459.200
45	36	1	0.1080	307105.600

46	36	1	0.1400	392459.200
47	38	1	0.1080	307105.600
48	38	1	0.1400	392459.200
49	39	1	0.1080	307105.600
50	39	1	0.1400	392459.200
51	40	1	0.1460	322168.000
52	42	1	0.1400	392459.200
53	44	1	0.1080	307105.600
54	44	1	0.1400	392459.200
55	46	1	0.1080	307105.600
56	48	1	0.1080	307105.600
57	48	1	0.1400	392459.200
58	50	1	0.1080	307105.600
59	51	1	0.1400	392459.200
60	51	1	0.1460	322168.000
61	52	1	0.1400	392459.200
62	52	1	0.1460	322168.000
63	54	1	0.1510	265265.600
64	57	1	0.1510	265265.600
65	59	1	0.1080	307105.600
66	60	1	0.1400	392459.200
67	60	1	0.1400	392459.200
68	61	1	0.1080	307105.600
69	62	1	0.1400	392459.200
70	62	1	0.1400	392459.200
71	63	1	0.1529	224262.400
72	63	1	0.1529	224262.400
73	63	1	0.1529	224262.400
74	71	1	0.1090	284512.000
75	71	1	0.1090	284512.000
76	71	1	0.1090	284512.000
77	72	1	0.1090	284512.000
78	72	1	0.1090	284512.000
79	72	1	0.1090	284512.000
80	73	1	0.1090	284512.000
81	73	1	0.1090	284512.000
82	73	1	0.1090	284512.000
83	64	1	0.1529	224262.400
84	64	1	0.1529	224262.400
85	64	1	0.1529	224262.400
86	83	1	0.1090	284512.000
87	83	1	0.1090	284512.000
88	83	1	0.1090	284512.000
89	84	1	0.1090	284512.000
90	84	1	0.1090	284512.000
91	84	1	0.1090	284512.000
92	85	1	0.1090	284512.000
93	85	1	0.1090	284512.000
94	85	1	0.1090	284512.000
95	66	1	0.1080	307105.600
96	66	1	0.1400	392459.200
97	67	1	0.1080	307105.600
98	67	1	0.1400	392459.200
99	69	1	0.1080	307105.600
100	69	1	0.1400	392459.200
101	70	1	0.1080	307105.600
102	70	1	0.1400	392459.200
103	96	1	0.1080	307105.600
104	96	1	0.1400	392459.200
105	98	1	0.1080	307105.600
106	100	1	0.1080	307105.600
107	100	1	0.1400	392459.200
108	102	1	0.1080	307105.600
109	104	1	0.1510	265265.600
110	107	1	0.1510	265265.600

111	109	1	0.1529	224262.400
112	109	1	0.1529	224262.400
113	109	1	0.1529	224262.400
114	111	1	0.1090	284512.000
115	111	1	0.1090	284512.000
116	111	1	0.1090	284512.000
117	112	1	0.1090	284512.000
118	112	1	0.1090	284512.000
119	112	1	0.1090	284512.000
120	113	1	0.1090	284512.000
121	113	1	0.1090	284512.000
122	113	1	0.1090	284512.000
123	110	1	0.1529	224262.400
124	110	1	0.1529	224262.400
125	110	1	0.1529	224262.400
126	123	1	0.1090	284512.000
127	123	1	0.1090	284512.000
128	123	1	0.1090	284512.000
129	124	1	0.1090	284512.000
130	124	1	0.1090	284512.000
131	124	1	0.1090	284512.000
132	125	1	0.1090	284512.000
133	125	1	0.1090	284512.000
134	125	1	0.1090	284512.000
9	7	1	0.1339	404174.400
23	18	1	0.1400	392459.200
21	19	1	0.1404	392459.200
30	28	1	0.1400	392459.200
42	40	1	0.1404	392459.200
54	46	1	0.1400	392459.200
57	50	1	0.1400	392459.200
61	59	1	0.1400	392459.200
104	98	1	0.1400	392459.200
107	102	1	0.1400	392459.200

[angles]

; ai	aj	ak	funct	c0	c1	c2	c3
1	2	3	1	120.000	292.880		
1	2	4	1	120.000	292.880		
2	3	5	1	120.000	292.880		
2	3	6	1	120.000	527.184		
2	4	7	1	124.000	585.760		
2	4	8	1	120.000	527.184		
3	6	9	1	120.000	527.184		
3	6	10	1	120.000	527.184		
4	8	11	1	124.000	585.760		
4	8	12	1	120.000	527.184		
6	9	13	1	120.000	292.880		
6	10	14	1	130.000	585.760		
6	10	15	1	125.000	585.760		
8	11	16	1	117.000	585.760		
8	12	17	1	120.000	292.880		
8	12	18	1	120.000	527.184		
10	14	19	1	110.000	585.760		
10	15	20	1	120.000	292.880		
10	15	21	1	109.800	585.760		
11	16	22	1	116.000	292.880		
11	16	23	1	124.000	585.760		
12	18	24	1	120.000	292.880		
14	19	25	1	111.000	585.760		
15	21	26	1	108.700	585.760		
16	23	27	1	120.000	527.184		
19	25	28	1	120.000	527.184		
19	25	29	1	120.000	527.184		
21	26	30	1	120.000	527.184		

21	26	31	1	120.000	527.184
23	27	32	1	130.000	585.760
23	27	33	1	125.000	585.760
25	28	34	1	120.000	292.880
25	29	35	1	120.000	527.184
25	29	36	1	120.000	527.184
26	30	37	1	120.000	292.880
26	31	38	1	120.000	527.184
26	31	39	1	120.000	527.184
27	32	40	1	110.000	585.760
27	33	41	1	120.000	292.880
27	33	42	1	109.800	585.760
29	35	43	1	120.000	292.880
29	35	44	1	120.000	527.184
29	36	45	1	120.000	292.880
29	36	46	1	120.000	527.184
31	38	47	1	120.000	292.880
31	38	48	1	120.000	527.184
31	39	49	1	120.000	292.880
31	39	50	1	120.000	527.184
32	40	51	1	111.000	585.760
33	42	52	1	108.700	585.760
35	44	53	1	120.000	292.880
35	44	54	1	120.000	527.184
36	46	55	1	120.000	292.880
38	48	56	1	120.000	292.880
38	48	57	1	120.000	527.184
39	50	58	1	120.000	292.880
40	51	59	1	120.000	527.184
40	51	60	1	120.000	527.184
42	52	61	1	120.000	527.184
42	52	62	1	120.000	527.184
44	54	63	1	120.000	585.760
48	57	64	1	120.000	585.760
51	59	65	1	120.000	292.880
51	60	66	1	120.000	527.184
51	60	67	1	120.000	527.184
52	61	68	1	120.000	292.880
52	62	69	1	120.000	527.184
52	62	70	1	120.000	527.184
54	63	71	1	114.000	527.184
54	63	72	1	114.000	527.184
54	63	73	1	114.000	527.184
63	71	74	1	110.700	313.800
63	71	75	1	110.700	313.800
63	71	76	1	110.700	313.800
63	72	77	1	110.700	313.800
63	72	78	1	110.700	313.800
63	72	79	1	110.700	313.800
63	73	80	1	110.700	313.800
63	73	81	1	110.700	313.800
63	73	82	1	110.700	313.800
57	64	83	1	114.000	527.184
57	64	84	1	114.000	527.184
57	64	85	1	114.000	527.184
64	83	86	1	110.700	313.800
64	83	87	1	110.700	313.800
64	83	88	1	110.700	313.800
64	84	89	1	110.700	313.800
64	84	90	1	110.700	313.800
64	84	91	1	110.700	313.800
64	85	92	1	110.700	313.800
64	85	93	1	110.700	313.800
64	85	94	1	110.700	313.800
60	66	95	1	120.000	292.880

60	66	96	1	120.000	527.184
60	67	97	1	120.000	292.880
60	67	98	1	120.000	527.184
62	69	99	1	120.000	292.880
62	69	100	1	120.000	527.184
62	70	101	1	120.000	292.880
62	70	102	1	120.000	527.184
66	96	103	1	120.000	292.880
66	96	104	1	120.000	527.184
67	98	105	1	120.000	292.880
69	100	106	1	120.000	292.880
69	100	107	1	120.000	527.184
70	102	108	1	120.000	292.880
96	104	109	1	120.000	585.760
100	107	110	1	120.000	585.760
104	109	111	1	114.000	527.184
104	109	112	1	114.000	527.184
104	109	113	1	114.000	527.184
109	111	114	1	110.700	313.800
109	111	115	1	110.700	313.800
109	111	116	1	110.700	313.800
109	112	117	1	110.700	313.800
109	112	118	1	110.700	313.800
109	112	119	1	110.700	313.800
109	113	120	1	110.700	313.800
109	113	121	1	110.700	313.800
109	113	122	1	110.700	313.800
107	110	123	1	114.000	527.184
107	110	124	1	114.000	527.184
107	110	125	1	114.000	527.184
110	123	126	1	110.700	313.800
110	123	127	1	110.700	313.800
110	123	128	1	110.700	313.800
110	124	129	1	110.700	313.800
110	124	130	1	110.700	313.800
110	124	131	1	110.700	313.800
110	125	132	1	110.700	313.800
110	125	133	1	110.700	313.800
110	125	134	1	110.700	313.800
52	61	59	1	120.000	527.184
7	4	8	1	124.000	585.760
54	46	55	1	120.000	292.880
28	30	37	1	120.000	292.880
71	63	72	1	112.700	488.273
129	124	131	1	107.800	276.144
45	36	46	1	120.000	292.880
127	123	128	1	107.800	276.144
14	19	21	1	111.000	585.760
74	71	76	1	107.800	276.144
57	50	58	1	120.000	292.880
77	72	78	1	107.800	276.144
59	61	68	1	120.000	292.880
23	18	24	1	120.000	292.880
106	100	107	1	120.000	292.880
80	73	82	1	107.800	276.144
87	83	88	1	107.800	276.144
50	57	64	1	120.000	585.760
15	21	19	1	108.700	585.760
72	63	73	1	112.700	488.273
111	109	112	1	112.700	488.273
83	64	84	1	112.700	488.273
120	113	122	1	107.800	276.144
84	64	85	1	112.700	488.273
89	84	90	1	107.800	276.144
90	84	91	1	107.800	276.144

40	42	52	1	134.900	711.280
61	59	65	1	120.000	292.880
18	23	27	1	120.000	527.184
33	42	40	1	108.700	585.760
20	15	21	1	125.800	251.040
53	44	54	1	120.000	292.880
56	48	57	1	120.000	292.880
69	62	70	1	120.000	527.184
126	123	127	1	107.800	276.144
98	104	109	1	120.000	585.760
3	2	4	1	120.000	527.184
114	111	116	1	107.800	276.144
102	107	110	1	120.000	585.760
115	111	116	1	107.800	276.144
99	69	100	1	120.000	292.880
9	6	10	1	120.000	527.184
81	73	82	1	107.800	276.144
66	60	67	1	120.000	527.184
114	111	115	1	107.800	276.144
28	25	29	1	120.000	527.184
101	70	102	1	120.000	292.880
126	123	128	1	107.800	276.144
132	125	133	1	107.800	276.144
103	96	104	1	120.000	292.880
5	3	6	1	120.000	292.880
121	113	122	1	107.800	276.144
117	112	118	1	107.800	276.144
97	67	98	1	120.000	292.880
123	110	124	1	112.700	488.273
111	109	113	1	112.700	488.273
43	35	44	1	120.000	292.880
117	112	119	1	107.800	276.144
35	29	36	1	120.000	527.184
124	110	125	1	112.700	488.273
123	110	125	1	112.700	488.273
25	28	30	1	120.000	527.184
86	83	88	1	107.800	276.144
118	112	119	1	107.800	276.144
74	71	75	1	107.800	276.144
78	72	79	1	107.800	276.144
11	8	12	1	124.000	585.760
36	46	54	1	120.000	527.184
17	12	18	1	120.000	292.880
47	38	48	1	120.000	292.880
71	63	73	1	112.700	488.273
80	73	81	1	107.800	276.144
32	27	33	1	120.000	585.760
30	28	34	1	120.000	292.880
32	40	42	1	111.000	585.760
16	23	18	1	120.000	527.184
49	39	50	1	120.000	292.880
75	71	76	1	107.800	276.144
61	52	62	1	120.000	527.184
26	30	28	1	120.000	527.184
92	85	93	1	107.800	276.144
39	50	57	1	120.000	527.184
51	59	61	1	120.000	527.184
83	64	85	1	112.700	488.273
92	85	94	1	107.800	276.144
38	31	39	1	120.000	527.184
42	40	51	1	120.000	527.184
96	104	98	1	120.000	527.184
21	19	25	1	120.000	527.184
70	102	107	1	120.000	527.184
46	54	63	1	120.000	585.760

95	66	96	1	120.000	292.880
100	107	102	1	120.000	527.184
132	125	134	1	107.800	276.144
133	125	134	1	107.800	276.144
30	26	31	1	120.000	527.184
67	98	104	1	120.000	527.184
44	54	46	1	120.000	527.184
77	72	79	1	107.800	276.144
41	33	42	1	125.800	251.040
129	124	130	1	107.800	276.144
6	9	7	1	124.000	585.760
112	109	113	1	112.700	488.273
7	9	13	1	116.000	292.880
93	85	94	1	107.800	276.144
89	84	91	1	107.800	276.144
4	7	9	1	117.000	585.760
130	124	131	1	107.800	276.144
104	98	105	1	120.000	292.880
19	21	26	1	134.900	711.280
120	113	121	1	107.800	276.144
12	18	23	1	120.000	527.184
48	57	50	1	120.000	527.184
59	51	60	1	120.000	527.184
107	102	108	1	120.000	292.880
22	16	23	1	120.000	292.880
86	83	87	1	107.800	276.144
14	10	15	1	120.000	585.760

[dihedrals]

; IMPROPER DIHEDRAL ANGLES

; ai	aj	ak	al	funct	c0	c1	c2	c3	c4	c5
8	4	2	7	4	180.000	10.460	2			
62	52	42	61	4	180.000	10.460	2			
31	26	21	30	4	180.000	10.460	2			
29	25	19	28	4	180.000	10.460	2			
60	51	40	59	4	180.000	10.460	2			
6	3	2	5	4	180.000	10.460	2			
4	2	1	3	4	180.000	10.460	2			
23	16	11	22	4	180.000	10.460	2			
26	21	15	19	4	180.000	10.460	2			
52	42	33	40	4	180.000	10.460	2			
25	19	14	21	4	180.000	10.460	2			
51	40	32	42	4	180.000	10.460	2			
70	62	52	69	4	180.000	10.460	2			
67	60	51	66	4	180.000	10.460	2			
36	29	25	35	4	180.000	10.460	2			
39	31	26	38	4	180.000	10.460	2			
12	8	4	11	4	180.000	10.460	2			
100	69	62	99	4	180.000	10.460	2			
44	35	29	43	4	180.000	10.460	2			
46	36	29	45	4	180.000	10.460	2			
102	70	62	101	4	180.000	10.460	2			
98	67	60	97	4	180.000	10.460	2			
96	66	60	95	4	180.000	10.460	2			
48	38	31	47	4	180.000	10.460	2			
18	12	8	17	4	180.000	10.460	2			
50	39	31	49	4	180.000	10.460	2			
57	48	38	56	4	180.000	10.460	2			
54	44	35	53	4	180.000	10.460	2			
107	100	69	106	4	180.000	10.460	2			
104	96	66	103	4	180.000	10.460	2			
21	15	10	20	4	180.000	10.460	2			
42	33	27	41	4	180.000	10.460	2			
10	6	3	9	4	180.000	10.460	2			
27	23	16	18	4	180.000	10.460	2			

64	57	48	50	4	180.000	10.460	2
63	54	44	46	4	180.000	10.460	2
109	104	96	98	4	180.000	10.460	2
110	107	100	102	4	180.000	10.460	2
34	28	25	30	4	180.000	10.460	2
65	59	51	61	4	180.000	10.460	2
37	30	26	28	4	180.000	10.460	2
68	61	52	59	4	180.000	10.460	2
13	9	6	7	4	180.000	10.460	2
24	18	12	23	4	180.000	10.460	2
105	98	67	104	4	180.000	10.460	2
55	46	36	54	4	180.000	10.460	2
58	50	39	57	4	180.000	10.460	2
108	102	70	107	4	180.000	10.460	2
15	10	6	14	4	180.000	10.460	2
33	27	23	32	4	180.000	10.460	2

[dihedrals]

; PROPER DIHEDRAL ANGLES

; ai	aj	ak	al	funct	c0	c1	c2	c3	c4	c5
62	52	61	59	3	30.334	0.000	-30.334	-0.000	-0.000	0.000
31	26	30	28	3	30.334	0.000	-30.334	-0.000	-0.000	0.000
8	4	2	3	3	30.334	0.000	-30.334	-0.000	-0.000	0.000
8	4	2	1	3	30.334	0.000	-30.334	-0.000	-0.000	0.000
62	52	42	40	3	30.334	0.000	-30.334	-0.000	-0.000	0.000
31	26	21	19	3	30.334	0.000	-30.334	-0.000	-0.000	0.000
62	52	42	33	3	30.334	0.000	-30.334	-0.000	-0.000	0.000
31	26	21	15	3	30.334	0.000	-30.334	-0.000	-0.000	0.000
60	51	40	42	3	9.079	0.000	-9.079	-0.000	-0.000	0.000
29	25	19	21	3	9.079	0.000	-9.079	-0.000	-0.000	0.000
60	51	40	32	3	9.079	0.000	-9.079	-0.000	-0.000	0.000
29	25	19	14	3	9.079	0.000	-9.079	-0.000	-0.000	0.000
6	3	2	4	3	30.334	0.000	-30.334	-0.000	-0.000	0.000
23	18	12	8	3	30.334	0.000	-30.334	-0.000	-0.000	0.000
26	30	28	25	3	30.334	0.000	-30.334	-0.000	-0.000	0.000
52	61	59	51	3	30.334	0.000	-30.334	-0.000	-0.000	0.000
23	18	12	17	3	30.334	0.000	-30.334	-0.000	-0.000	0.000
6	3	2	1	3	30.334	0.000	-30.334	-0.000	-0.000	0.000
23	16	11	8	3	30.334	0.000	-30.334	-0.000	-0.000	0.000
6	9	7	4	3	30.334	0.000	-30.334	-0.000	-0.000	0.000
26	21	19	25	3	30.334	0.000	-30.334	-0.000	-0.000	0.000
52	42	40	51	3	30.334	0.000	-30.334	-0.000	-0.000	0.000
52	42	40	32	3	9.079	0.000	-9.079	-0.000	-0.000	0.000
26	21	19	14	3	9.079	0.000	-9.079	-0.000	-0.000	0.000
26	21	15	10	3	30.334	0.000	-30.334	-0.000	-0.000	0.000
52	42	33	27	3	30.334	0.000	-30.334	-0.000	-0.000	0.000
52	42	33	41	3	20.920	0.000	-20.920	-0.000	-0.000	0.000
26	21	15	20	3	20.920	0.000	-20.920	-0.000	-0.000	0.000
51	40	42	33	3	30.334	0.000	-30.334	-0.000	-0.000	0.000
25	19	21	15	3	30.334	0.000	-30.334	-0.000	-0.000	0.000
51	40	32	27	3	30.334	0.000	-30.334	-0.000	-0.000	0.000
25	19	14	10	3	30.334	0.000	-30.334	-0.000	-0.000	0.000
36	29	25	28	3	9.079	0.000	-9.079	-0.000	-0.000	0.000
35	29	25	28	3	9.079	0.000	-9.079	-0.000	-0.000	0.000
69	62	52	61	3	9.079	0.000	-9.079	-0.000	-0.000	0.000
70	62	52	61	3	9.079	0.000	-9.079	-0.000	-0.000	0.000
12	8	4	2	3	9.079	0.000	-9.079	-0.000	-0.000	0.000
67	60	51	59	3	9.079	0.000	-9.079	-0.000	-0.000	0.000
66	60	51	59	3	9.079	0.000	-9.079	-0.000	-0.000	0.000
39	31	26	30	3	9.079	0.000	-9.079	-0.000	-0.000	0.000
38	31	26	30	3	9.079	0.000	-9.079	-0.000	-0.000	0.000
69	62	52	42	3	9.079	0.000	-9.079	-0.000	-0.000	0.000
70	62	52	42	3	9.079	0.000	-9.079	-0.000	-0.000	0.000
39	31	26	21	3	9.079	0.000	-9.079	-0.000	-0.000	0.000
38	31	26	21	3	9.079	0.000	-9.079	-0.000	-0.000	0.000

36	29	25	19	3	9.079	0.000	-9.079	-0.000	-0.000	0.000
35	29	25	19	3	9.079	0.000	-9.079	-0.000	-0.000	0.000
67	60	51	40	3	9.079	0.000	-9.079	-0.000	-0.000	0.000
66	60	51	40	3	9.079	0.000	-9.079	-0.000	-0.000	0.000
12	8	4	7	3	9.079	0.000	-9.079	-0.000	-0.000	0.000
16	23	18	12	3	30.334	0.000	-30.334	-0.000	-0.000	0.000
9	6	3	2	3	30.334	0.000	-30.334	-0.000	-0.000	0.000
9	6	3	5	3	30.334	0.000	-30.334	-0.000	-0.000	0.000
18	23	16	11	3	30.334	0.000	-30.334	-0.000	-0.000	0.000
61	52	42	40	3	9.079	0.000	-9.079	-0.000	-0.000	0.000
30	26	21	19	3	9.079	0.000	-9.079	-0.000	-0.000	0.000
61	52	42	33	3	9.079	0.000	-9.079	-0.000	-0.000	0.000
30	26	21	15	3	9.079	0.000	-9.079	-0.000	-0.000	0.000
59	51	40	42	3	9.079	0.000	-9.079	-0.000	-0.000	0.000
28	25	19	21	3	9.079	0.000	-9.079	-0.000	-0.000	0.000
28	25	19	14	3	9.079	0.000	-9.079	-0.000	-0.000	0.000
59	51	40	32	3	9.079	0.000	-9.079	-0.000	-0.000	0.000
96	66	60	51	3	30.334	0.000	-30.334	-0.000	-0.000	0.000
102	70	62	52	3	30.334	0.000	-30.334	-0.000	-0.000	0.000
100	69	62	52	3	30.334	0.000	-30.334	-0.000	-0.000	0.000
61	59	51	60	3	30.334	0.000	-30.334	-0.000	-0.000	0.000
98	67	60	51	3	30.334	0.000	-30.334	-0.000	-0.000	0.000
30	28	25	29	3	30.334	0.000	-30.334	-0.000	-0.000	0.000
18	12	8	4	3	30.334	0.000	-30.334	-0.000	-0.000	0.000
50	39	31	26	3	30.334	0.000	-30.334	-0.000	-0.000	0.000
44	35	29	25	3	30.334	0.000	-30.334	-0.000	-0.000	0.000
48	38	31	26	3	30.334	0.000	-30.334	-0.000	-0.000	0.000
46	36	29	25	3	30.334	0.000	-30.334	-0.000	-0.000	0.000
44	35	29	36	3	30.334	0.000	-30.334	-0.000	-0.000	0.000
50	39	31	38	3	30.334	0.000	-30.334	-0.000	-0.000	0.000
48	38	31	39	3	30.334	0.000	-30.334	-0.000	-0.000	0.000
102	70	62	69	3	30.334	0.000	-30.334	-0.000	-0.000	0.000
100	69	62	70	3	30.334	0.000	-30.334	-0.000	-0.000	0.000
96	66	60	67	3	30.334	0.000	-30.334	-0.000	-0.000	0.000
98	67	60	66	3	30.334	0.000	-30.334	-0.000	-0.000	0.000
46	36	29	35	3	30.334	0.000	-30.334	-0.000	-0.000	0.000
28	30	26	21	3	30.334	0.000	-30.334	-0.000	-0.000	0.000
59	61	52	42	3	30.334	0.000	-30.334	-0.000	-0.000	0.000
30	28	25	19	3	30.334	0.000	-30.334	-0.000	-0.000	0.000
61	59	51	40	3	30.334	0.000	-30.334	-0.000	-0.000	0.000
18	12	8	11	3	30.334	0.000	-30.334	-0.000	-0.000	0.000
57	50	39	31	3	30.334	0.000	-30.334	-0.000	-0.000	0.000
104	98	67	60	3	30.334	0.000	-30.334	-0.000	-0.000	0.000
54	44	35	29	3	30.334	0.000	-30.334	-0.000	-0.000	0.000
107	100	69	62	3	30.334	0.000	-30.334	-0.000	-0.000	0.000
104	96	66	60	3	30.334	0.000	-30.334	-0.000	-0.000	0.000
54	46	36	29	3	30.334	0.000	-30.334	-0.000	-0.000	0.000
107	102	70	62	3	30.334	0.000	-30.334	-0.000	-0.000	0.000
57	48	38	31	3	30.334	0.000	-30.334	-0.000	-0.000	0.000
96	104	98	67	3	30.334	0.000	-30.334	-0.000	-0.000	0.000
98	104	96	66	3	30.334	0.000	-30.334	-0.000	-0.000	0.000
50	57	48	38	3	30.334	0.000	-30.334	-0.000	-0.000	0.000
44	54	46	36	3	30.334	0.000	-30.334	-0.000	-0.000	0.000
48	57	50	39	3	30.334	0.000	-30.334	-0.000	-0.000	0.000
100	107	102	70	3	30.334	0.000	-30.334	-0.000	-0.000	0.000
102	107	100	69	3	30.334	0.000	-30.334	-0.000	-0.000	0.000
46	54	44	35	3	30.334	0.000	-30.334	-0.000	-0.000	0.000
54	46	36	45	3	30.334	0.000	-30.334	-0.000	-0.000	0.000
57	48	38	47	3	30.334	0.000	-30.334	-0.000	-0.000	0.000
54	44	35	43	3	30.334	0.000	-30.334	-0.000	-0.000	0.000
107	102	70	101	3	30.334	0.000	-30.334	-0.000	-0.000	0.000
104	98	67	97	3	30.334	0.000	-30.334	-0.000	-0.000	0.000
104	96	66	95	3	30.334	0.000	-30.334	-0.000	-0.000	0.000
107	100	69	99	3	30.334	0.000	-30.334	-0.000	-0.000	0.000
57	50	39	49	3	30.334	0.000	-30.334	-0.000	-0.000	0.000

9	7	4	8	3	30.334	0.000	-30.334	-0.000	-0.000	0.000
16	11	8	4	3	30.334	0.000	-30.334	-0.000	-0.000	0.000
16	11	8	12	3	30.334	0.000	-30.334	-0.000	-0.000	0.000
9	7	4	2	3	30.334	0.000	-30.334	-0.000	-0.000	0.000
42	40	32	27	3	30.334	0.000	-30.334	-0.000	-0.000	0.000
21	19	14	10	3	30.334	0.000	-30.334	-0.000	-0.000	0.000
21	15	10	6	3	0.000	0.000	0.000	-0.000	-0.000	0.000
42	33	27	23	3	0.000	0.000	0.000	-0.000	-0.000	0.000
42	33	27	32	3	41.840	0.000	-41.840	-0.000	-0.000	0.000
21	15	10	14	3	41.840	0.000	-41.840	-0.000	-0.000	0.000
27	23	18	12	3	30.334	0.000	-30.334	-0.000	-0.000	0.000
10	6	3	2	3	30.334	0.000	-30.334	-0.000	-0.000	0.000
27	23	18	24	3	30.334	0.000	-30.334	-0.000	-0.000	0.000
27	23	16	22	3	30.334	0.000	-30.334	-0.000	-0.000	0.000
10	6	3	5	3	30.334	0.000	-30.334	-0.000	-0.000	0.000
10	6	9	7	3	30.334	0.000	-30.334	-0.000	-0.000	0.000
27	23	16	11	3	30.334	0.000	-30.334	-0.000	-0.000	0.000
63	54	44	35	3	30.334	0.000	-30.334	-0.000	-0.000	0.000
63	54	46	36	3	30.334	0.000	-30.334	-0.000	-0.000	0.000
64	57	50	39	3	30.334	0.000	-30.334	-0.000	-0.000	0.000
110	107	100	69	3	30.334	0.000	-30.334	-0.000	-0.000	0.000
109	104	96	66	3	30.334	0.000	-30.334	-0.000	-0.000	0.000
109	104	98	67	3	30.334	0.000	-30.334	-0.000	-0.000	0.000
110	107	102	70	3	30.334	0.000	-30.334	-0.000	-0.000	0.000
64	57	48	38	3	30.334	0.000	-30.334	-0.000	-0.000	0.000
109	104	96	103	3	30.334	0.000	-30.334	-0.000	-0.000	0.000
109	104	98	105	3	30.334	0.000	-30.334	-0.000	-0.000	0.000
110	107	100	106	3	30.334	0.000	-30.334	-0.000	-0.000	0.000
64	57	50	58	3	30.334	0.000	-30.334	-0.000	-0.000	0.000
64	57	48	56	3	30.334	0.000	-30.334	-0.000	-0.000	0.000
63	54	44	53	3	30.334	0.000	-30.334	-0.000	-0.000	0.000
63	54	46	55	3	30.334	0.000	-30.334	-0.000	-0.000	0.000
110	107	102	108	3	30.334	0.000	-30.334	-0.000	-0.000	0.000
85	64	57	48	3	0.000	0.000	0.000	-0.000	-0.000	0.000
124	110	107	100	3	0.000	0.000	0.000	-0.000	-0.000	0.000
71	63	54	44	3	0.000	0.000	0.000	-0.000	-0.000	0.000
111	109	104	96	3	0.000	0.000	0.000	-0.000	-0.000	0.000
125	110	107	100	3	0.000	0.000	0.000	-0.000	-0.000	0.000
111	109	104	98	3	0.000	0.000	0.000	-0.000	-0.000	0.000
125	110	107	102	3	0.000	0.000	0.000	-0.000	-0.000	0.000
84	64	57	50	3	0.000	0.000	0.000	-0.000	-0.000	0.000
85	64	57	50	3	0.000	0.000	0.000	-0.000	-0.000	0.000
73	63	54	44	3	0.000	0.000	0.000	-0.000	-0.000	0.000
123	110	107	100	3	0.000	0.000	0.000	-0.000	-0.000	0.000
84	64	57	48	3	0.000	0.000	0.000	-0.000	-0.000	0.000
123	110	107	102	3	0.000	0.000	0.000	-0.000	-0.000	0.000
112	109	104	98	3	0.000	0.000	0.000	-0.000	-0.000	0.000
72	63	54	44	3	0.000	0.000	0.000	-0.000	-0.000	0.000
113	109	104	98	3	0.000	0.000	0.000	-0.000	-0.000	0.000
83	64	57	48	3	0.000	0.000	0.000	-0.000	-0.000	0.000
83	64	57	50	3	0.000	0.000	0.000	-0.000	-0.000	0.000
113	109	104	96	3	0.000	0.000	0.000	-0.000	-0.000	0.000
72	63	54	46	3	0.000	0.000	0.000	-0.000	-0.000	0.000
71	63	54	46	3	0.000	0.000	0.000	-0.000	-0.000	0.000
124	110	107	102	3	0.000	0.000	0.000	-0.000	-0.000	0.000
112	109	104	96	3	0.000	0.000	0.000	-0.000	-0.000	0.000
73	63	54	46	3	0.000	0.000	0.000	-0.000	-0.000	0.000
40	42	33	27	3	30.334	0.000	-30.334	-0.000	-0.000	0.000
19	21	15	10	3	30.334	0.000	-30.334	-0.000	-0.000	0.000
19	14	10	6	3	30.334	0.000	-30.334	-0.000	-0.000	0.000
40	32	27	23	3	30.334	0.000	-30.334	-0.000	-0.000	0.000
19	14	10	15	3	41.840	0.000	-41.840	-0.000	-0.000	0.000
40	32	27	33	3	41.840	0.000	-41.840	-0.000	-0.000	0.000
20	15	21	19	3	20.920	0.000	-20.920	-0.000	-0.000	0.000
41	33	42	40	3	20.920	0.000	-20.920	-0.000	-0.000	0.000

41	33	27	23	3	20.920	0.000	-20.920	-0.000	-0.000	0.000
20	15	10	6	3	20.920	0.000	-20.920	-0.000	-0.000	0.000
20	15	10	14	3	20.920	0.000	-20.920	-0.000	-0.000	0.000
41	33	27	32	3	20.920	0.000	-20.920	-0.000	-0.000	0.000
95	66	60	51	3	30.334	0.000	-30.334	-0.000	-0.000	0.000
17	12	8	4	3	30.334	0.000	-30.334	-0.000	-0.000	0.000
101	70	62	52	3	30.334	0.000	-30.334	-0.000	-0.000	0.000
65	59	51	60	3	30.334	0.000	-30.334	-0.000	-0.000	0.000
68	61	52	62	3	30.334	0.000	-30.334	-0.000	-0.000	0.000
47	38	31	26	3	30.334	0.000	-30.334	-0.000	-0.000	0.000
37	30	26	31	3	30.334	0.000	-30.334	-0.000	-0.000	0.000
34	28	25	29	3	30.334	0.000	-30.334	-0.000	-0.000	0.000
97	67	60	51	3	30.334	0.000	-30.334	-0.000	-0.000	0.000
49	39	31	26	3	30.334	0.000	-30.334	-0.000	-0.000	0.000
45	36	29	25	3	30.334	0.000	-30.334	-0.000	-0.000	0.000
99	69	62	52	3	30.334	0.000	-30.334	-0.000	-0.000	0.000
43	35	29	25	3	30.334	0.000	-30.334	-0.000	-0.000	0.000
49	39	31	38	3	30.334	0.000	-30.334	-0.000	-0.000	0.000
97	67	60	66	3	30.334	0.000	-30.334	-0.000	-0.000	0.000
99	69	62	70	3	30.334	0.000	-30.334	-0.000	-0.000	0.000
13	9	6	3	3	30.334	0.000	-30.334	-0.000	-0.000	0.000
22	16	23	18	3	30.334	0.000	-30.334	-0.000	-0.000	0.000
45	36	29	35	3	30.334	0.000	-30.334	-0.000	-0.000	0.000
43	35	29	36	3	30.334	0.000	-30.334	-0.000	-0.000	0.000
101	70	62	69	3	30.334	0.000	-30.334	-0.000	-0.000	0.000
47	38	31	39	3	30.334	0.000	-30.334	-0.000	-0.000	0.000
24	18	23	16	3	30.334	0.000	-30.334	-0.000	-0.000	0.000
95	66	60	67	3	30.334	0.000	-30.334	-0.000	-0.000	0.000
68	61	52	42	3	30.334	0.000	-30.334	-0.000	-0.000	0.000
37	30	26	21	3	30.334	0.000	-30.334	-0.000	-0.000	0.000
13	9	6	10	3	30.334	0.000	-30.334	-0.000	-0.000	0.000
34	28	25	19	3	30.334	0.000	-30.334	-0.000	-0.000	0.000
65	59	51	40	3	30.334	0.000	-30.334	-0.000	-0.000	0.000
17	12	8	11	3	30.334	0.000	-30.334	-0.000	-0.000	0.000
58	50	39	31	3	30.334	0.000	-30.334	-0.000	-0.000	0.000
65	59	61	52	3	30.334	0.000	-30.334	-0.000	-0.000	0.000
103	96	66	60	3	30.334	0.000	-30.334	-0.000	-0.000	0.000
55	46	36	29	3	30.334	0.000	-30.334	-0.000	-0.000	0.000
106	100	69	62	3	30.334	0.000	-30.334	-0.000	-0.000	0.000
5	3	2	4	3	30.334	0.000	-30.334	-0.000	-0.000	0.000
68	61	59	51	3	30.334	0.000	-30.334	-0.000	-0.000	0.000
108	102	70	62	3	30.334	0.000	-30.334	-0.000	-0.000	0.000
24	18	12	8	3	30.334	0.000	-30.334	-0.000	-0.000	0.000
34	28	30	26	3	30.334	0.000	-30.334	-0.000	-0.000	0.000
37	30	28	25	3	30.334	0.000	-30.334	-0.000	-0.000	0.000
105	98	67	60	3	30.334	0.000	-30.334	-0.000	-0.000	0.000
53	44	35	29	3	30.334	0.000	-30.334	-0.000	-0.000	0.000
56	48	38	31	3	30.334	0.000	-30.334	-0.000	-0.000	0.000
58	50	57	48	3	30.334	0.000	-30.334	-0.000	-0.000	0.000
56	48	57	50	3	30.334	0.000	-30.334	-0.000	-0.000	0.000
103	96	104	98	3	30.334	0.000	-30.334	-0.000	-0.000	0.000
55	46	54	44	3	30.334	0.000	-30.334	-0.000	-0.000	0.000
106	100	107	102	3	30.334	0.000	-30.334	-0.000	-0.000	0.000
53	44	54	46	3	30.334	0.000	-30.334	-0.000	-0.000	0.000
105	98	104	96	3	30.334	0.000	-30.334	-0.000	-0.000	0.000
108	102	107	100	3	30.334	0.000	-30.334	-0.000	-0.000	0.000
105	98	67	97	3	30.334	0.000	-30.334	-0.000	-0.000	0.000
55	46	36	45	3	30.334	0.000	-30.334	-0.000	-0.000	0.000
5	3	2	1	3	30.334	0.000	-30.334	-0.000	-0.000	0.000
106	100	69	99	3	30.334	0.000	-30.334	-0.000	-0.000	0.000
53	44	35	43	3	30.334	0.000	-30.334	-0.000	-0.000	0.000
58	50	39	49	3	30.334	0.000	-30.334	-0.000	-0.000	0.000
68	61	59	65	3	30.334	0.000	-30.334	-0.000	-0.000	0.000
37	30	28	34	3	30.334	0.000	-30.334	-0.000	-0.000	0.000
108	102	70	101	3	30.334	0.000	-30.334	-0.000	-0.000	0.000

24	18	12	17	3	30.334	0.000	-30.334	-0.000	-0.000	0.000
103	96	66	95	3	30.334	0.000	-30.334	-0.000	-0.000	0.000
56	48	38	47	3	30.334	0.000	-30.334	-0.000	-0.000	0.000
22	16	11	8	3	30.334	0.000	-30.334	-0.000	-0.000	0.000
13	9	7	4	3	30.334	0.000	-30.334	-0.000	-0.000	0.000
74	71	63	54	3	0.967	2.900	0.000	-3.866	-0.000	0.000
80	73	63	54	3	0.967	2.900	0.000	-3.866	-0.000	0.000
81	73	63	54	3	0.967	2.900	0.000	-3.866	-0.000	0.000
77	72	63	54	3	0.967	2.900	0.000	-3.866	-0.000	0.000
90	84	64	57	3	0.967	2.900	0.000	-3.866	-0.000	0.000
130	124	110	107	3	0.967	2.900	0.000	-3.866	-0.000	0.000
82	73	63	54	3	0.967	2.900	0.000	-3.866	-0.000	0.000
92	85	64	57	3	0.967	2.900	0.000	-3.866	-0.000	0.000
89	84	64	57	3	0.967	2.900	0.000	-3.866	-0.000	0.000
94	85	64	57	3	0.967	2.900	0.000	-3.866	-0.000	0.000
86	83	64	57	3	0.967	2.900	0.000	-3.866	-0.000	0.000
93	85	64	57	3	0.967	2.900	0.000	-3.866	-0.000	0.000
118	112	109	104	3	0.967	2.900	0.000	-3.866	-0.000	0.000
91	84	64	57	3	0.967	2.900	0.000	-3.866	-0.000	0.000
126	123	110	107	3	0.967	2.900	0.000	-3.866	-0.000	0.000
128	123	110	107	3	0.967	2.900	0.000	-3.866	-0.000	0.000
133	125	110	107	3	0.967	2.900	0.000	-3.866	-0.000	0.000
122	113	109	104	3	0.967	2.900	0.000	-3.866	-0.000	0.000
78	72	63	54	3	0.967	2.900	0.000	-3.866	-0.000	0.000
88	83	64	57	3	0.967	2.900	0.000	-3.866	-0.000	0.000
87	83	64	57	3	0.967	2.900	0.000	-3.866	-0.000	0.000
76	71	63	54	3	0.967	2.900	0.000	-3.866	-0.000	0.000
132	125	110	107	3	0.967	2.900	0.000	-3.866	-0.000	0.000
75	71	63	54	3	0.967	2.900	0.000	-3.866	-0.000	0.000
129	124	110	107	3	0.967	2.900	0.000	-3.866	-0.000	0.000
134	125	110	107	3	0.967	2.900	0.000	-3.866	-0.000	0.000
119	112	109	104	3	0.967	2.900	0.000	-3.866	-0.000	0.000
120	113	109	104	3	0.967	2.900	0.000	-3.866	-0.000	0.000
117	112	109	104	3	0.967	2.900	0.000	-3.866	-0.000	0.000
115	111	109	104	3	0.967	2.900	0.000	-3.866	-0.000	0.000
114	111	109	104	3	0.967	2.900	0.000	-3.866	-0.000	0.000
127	123	110	107	3	0.967	2.900	0.000	-3.866	-0.000	0.000
131	124	110	107	3	0.967	2.900	0.000	-3.866	-0.000	0.000
121	113	109	104	3	0.967	2.900	0.000	-3.866	-0.000	0.000
116	111	109	104	3	0.967	2.900	0.000	-3.866	-0.000	0.000
79	72	63	54	3	0.967	2.900	0.000	-3.866	-0.000	0.000
94	85	64	83	3	0.628	1.883	0.000	-2.510	-0.000	0.000
93	85	64	84	3	0.628	1.883	0.000	-2.510	-0.000	0.000
119	112	109	113	3	0.628	1.883	0.000	-2.510	-0.000	0.000
114	111	109	112	3	0.628	1.883	0.000	-2.510	-0.000	0.000
132	125	110	123	3	0.628	1.883	0.000	-2.510	-0.000	0.000
74	71	63	72	3	0.628	1.883	0.000	-2.510	-0.000	0.000
131	124	110	125	3	0.628	1.883	0.000	-2.510	-0.000	0.000
88	83	64	85	3	0.628	1.883	0.000	-2.510	-0.000	0.000
128	123	110	124	3	0.628	1.883	0.000	-2.510	-0.000	0.000
119	112	109	111	3	0.628	1.883	0.000	-2.510	-0.000	0.000
90	84	64	85	3	0.628	1.883	0.000	-2.510	-0.000	0.000
134	125	110	124	3	0.628	1.883	0.000	-2.510	-0.000	0.000
92	85	64	83	3	0.628	1.883	0.000	-2.510	-0.000	0.000
130	124	110	125	3	0.628	1.883	0.000	-2.510	-0.000	0.000
114	111	109	113	3	0.628	1.883	0.000	-2.510	-0.000	0.000
120	113	109	111	3	0.628	1.883	0.000	-2.510	-0.000	0.000
117	112	109	113	3	0.628	1.883	0.000	-2.510	-0.000	0.000
127	123	110	125	3	0.628	1.883	0.000	-2.510	-0.000	0.000
77	72	63	71	3	0.628	1.883	0.000	-2.510	-0.000	0.000
82	73	63	72	3	0.628	1.883	0.000	-2.510	-0.000	0.000
91	84	64	83	3	0.628	1.883	0.000	-2.510	-0.000	0.000
89	84	64	85	3	0.628	1.883	0.000	-2.510	-0.000	0.000
76	71	63	73	3	0.628	1.883	0.000	-2.510	-0.000	0.000
92	85	64	84	3	0.628	1.883	0.000	-2.510	-0.000	0.000

82	73	63	71	3	0.628	1.883	0.000	-2.510	-0.000	0.000
81	73	63	72	3	0.628	1.883	0.000	-2.510	-0.000	0.000
129	124	110	123	3	0.628	1.883	0.000	-2.510	-0.000	0.000
93	85	64	83	3	0.628	1.883	0.000	-2.510	-0.000	0.000
118	112	109	111	3	0.628	1.883	0.000	-2.510	-0.000	0.000
81	73	63	71	3	0.628	1.883	0.000	-2.510	-0.000	0.000
115	111	109	113	3	0.628	1.883	0.000	-2.510	-0.000	0.000
117	112	109	111	3	0.628	1.883	0.000	-2.510	-0.000	0.000
118	112	109	113	3	0.628	1.883	0.000	-2.510	-0.000	0.000
132	125	110	124	3	0.628	1.883	0.000	-2.510	-0.000	0.000
134	125	110	123	3	0.628	1.883	0.000	-2.510	-0.000	0.000
133	125	110	124	3	0.628	1.883	0.000	-2.510	-0.000	0.000
78	72	63	73	3	0.628	1.883	0.000	-2.510	-0.000	0.000
133	125	110	123	3	0.628	1.883	0.000	-2.510	-0.000	0.000
78	72	63	71	3	0.628	1.883	0.000	-2.510	-0.000	0.000
128	123	110	125	3	0.628	1.883	0.000	-2.510	-0.000	0.000
79	72	63	73	3	0.628	1.883	0.000	-2.510	-0.000	0.000
86	83	64	84	3	0.628	1.883	0.000	-2.510	-0.000	0.000
115	111	109	112	3	0.628	1.883	0.000	-2.510	-0.000	0.000
80	73	63	72	3	0.628	1.883	0.000	-2.510	-0.000	0.000
89	84	64	83	3	0.628	1.883	0.000	-2.510	-0.000	0.000
80	73	63	71	3	0.628	1.883	0.000	-2.510	-0.000	0.000
116	111	109	113	3	0.628	1.883	0.000	-2.510	-0.000	0.000
77	72	63	73	3	0.628	1.883	0.000	-2.510	-0.000	0.000
79	72	63	71	3	0.628	1.883	0.000	-2.510	-0.000	0.000
88	83	64	84	3	0.628	1.883	0.000	-2.510	-0.000	0.000
122	113	109	112	3	0.628	1.883	0.000	-2.510	-0.000	0.000
76	71	63	72	3	0.628	1.883	0.000	-2.510	-0.000	0.000
122	113	109	111	3	0.628	1.883	0.000	-2.510	-0.000	0.000
121	113	109	111	3	0.628	1.883	0.000	-2.510	-0.000	0.000
121	113	109	112	3	0.628	1.883	0.000	-2.510	-0.000	0.000
116	111	109	112	3	0.628	1.883	0.000	-2.510	-0.000	0.000
86	83	64	85	3	0.628	1.883	0.000	-2.510	-0.000	0.000
90	84	64	83	3	0.628	1.883	0.000	-2.510	-0.000	0.000
75	71	63	73	3	0.628	1.883	0.000	-2.510	-0.000	0.000
120	113	109	112	3	0.628	1.883	0.000	-2.510	-0.000	0.000
126	123	110	124	3	0.628	1.883	0.000	-2.510	-0.000	0.000
94	85	64	84	3	0.628	1.883	0.000	-2.510	-0.000	0.000
127	123	110	124	3	0.628	1.883	0.000	-2.510	-0.000	0.000
75	71	63	72	3	0.628	1.883	0.000	-2.510	-0.000	0.000
131	124	110	123	3	0.628	1.883	0.000	-2.510	-0.000	0.000
129	124	110	125	3	0.628	1.883	0.000	-2.510	-0.000	0.000
130	124	110	123	3	0.628	1.883	0.000	-2.510	-0.000	0.000
87	83	64	84	3	0.628	1.883	0.000	-2.510	-0.000	0.000
126	123	110	125	3	0.628	1.883	0.000	-2.510	-0.000	0.000
91	84	64	85	3	0.628	1.883	0.000	-2.510	-0.000	0.000
74	71	63	73	3	0.628	1.883	0.000	-2.510	-0.000	0.000
87	83	64	85	3	0.628	1.883	0.000	-2.510	-0.000	0.000
15	21	19	14	3	9.079	0.000	-9.079	-0.000	-0.000	0.000
33	42	40	32	3	9.079	0.000	-9.079	-0.000	-0.000	0.000
15	10	6	3	3	9.079	0.000	-9.079	-0.000	-0.000	0.000
33	27	23	18	3	9.079	0.000	-9.079	-0.000	-0.000	0.000
15	10	6	9	3	9.079	0.000	-9.079	-0.000	-0.000	0.000
33	27	23	16	3	9.079	0.000	-9.079	-0.000	-0.000	0.000
32	27	23	16	3	9.079	0.000	-9.079	-0.000	-0.000	0.000
32	27	23	18	3	9.079	0.000	-9.079	-0.000	-0.000	0.000
14	10	6	9	3	9.079	0.000	-9.079	-0.000	-0.000	0.000
14	10	6	3	3	9.079	0.000	-9.079	-0.000	-0.000	0.000
11	8	4	2	3	9.079	0.000	-9.079	-0.000	-0.000	0.000
11	8	4	7	3	9.079	0.000	-9.079	-0.000	-0.000	0.000
7	4	2	3	3	30.334	0.000	-30.334	-0.000	-0.000	0.000
7	4	2	1	3	30.334	0.000	-30.334	-0.000	-0.000	0.000
7	9	6	3	3	30.334	0.000	-30.334	-0.000	-0.000	0.000

[pairs]

1	5	1
1	6	1
1	7	1
4	5	1
1	8	1
4	6	1
3	7	1
3	8	1
2	9	1
2	10	1
2	11	1
5	9	1
2	12	1
5	10	1
3	13	1
8	9	1
7	10	1
4	13	1
3	14	1
7	11	1
3	15	1
7	12	1
4	16	1
4	17	1
4	18	1
10	13	1
9	14	1
9	15	1
6	19	1
6	20	1
6	21	1
12	16	1
11	17	1
11	18	1
8	22	1
8	23	1
8	24	1
14	20	1
10	25	1
10	26	1
11	27	1
19	20	1
12	27	1
18	22	1
17	23	1
16	24	1
15	25	1
14	26	1
17	24	1
14	28	1
14	29	1
15	30	1
20	26	1
15	31	1
16	32	1
22	27	1
21	28	1
19	30	1
16	33	1
21	29	1
19	31	1
18	32	1
25	26	1
24	27	1
18	33	1

19	34	1
19	35	1
19	36	1
21	37	1
29	30	1
28	31	1
21	38	1
26	34	1
21	39	1
25	37	1
29	34	1
28	35	1
23	40	1
28	36	1
23	41	1
23	42	1
31	37	1
30	38	1
25	43	1
30	39	1
25	44	1
25	45	1
34	37	1
25	46	1
32	41	1
26	47	1
26	48	1
26	49	1
26	50	1
27	51	1
36	43	1
27	52	1
36	44	1
35	45	1
40	41	1
35	46	1
29	53	1
29	54	1
33	51	1
32	52	1
29	55	1
39	47	1
39	48	1
38	49	1
31	56	1
38	50	1
31	57	1
31	58	1
32	59	1
32	60	1
41	52	1
33	61	1
33	62	1
43	53	1
43	54	1
35	63	1
46	53	1
45	54	1
44	55	1
36	63	1
45	55	1
42	59	1
40	61	1
42	60	1
40	62	1

38	64	1
51	52	1
47	56	1
39	64	1
47	57	1
40	65	1
50	56	1
49	57	1
48	58	1
40	66	1
49	58	1
40	67	1
42	68	1
42	69	1
42	70	1
44	71	1
53	63	1
44	72	1
52	65	1
46	71	1
44	73	1
55	63	1
46	72	1
51	68	1
46	73	1
56	64	1
60	61	1
59	62	1
58	64	1
60	65	1
59	66	1
59	67	1
54	74	1
54	75	1
62	68	1
61	69	1
54	76	1
61	70	1
54	77	1
48	83	1
54	78	1
48	84	1
65	68	1
54	79	1
50	83	1
48	85	1
54	80	1
50	84	1
54	81	1
50	85	1
54	82	1
57	86	1
57	87	1
57	88	1
72	74	1
57	89	1
51	95	1
73	74	1
72	75	1
57	90	1
51	96	1
73	75	1
72	76	1
71	77	1
57	91	1

51	97	1
73	76	1
71	78	1
57	92	1
51	98	1
73	77	1
71	79	1
57	93	1
73	78	1
71	80	1
57	94	1
52	99	1
73	79	1
72	80	1
71	81	1
52	100	1
72	81	1
71	82	1
52	101	1
72	82	1
52	102	1
67	95	1
67	96	1
66	97	1
60	103	1
66	98	1
60	104	1
60	105	1
62	106	1
70	99	1
62	107	1
84	86	1
70	100	1
69	101	1
62	108	1
85	86	1
84	87	1
69	102	1
85	87	1
84	88	1
83	89	1
85	88	1
83	90	1
85	89	1
83	91	1
85	90	1
83	92	1
66	109	1
85	91	1
84	92	1
83	93	1
67	109	1
84	93	1
83	94	1
84	94	1
69	110	1
70	110	1
95	103	1
95	104	1
98	103	1
97	104	1
96	105	1
97	105	1
99	106	1
99	107	1

96	111	1
102	106	1
101	107	1
100	108	1
96	112	1
101	108	1
98	111	1
96	113	1
98	112	1
98	113	1
103	109	1
105	109	1
106	110	1
108	110	1
104	114	1
104	115	1
104	116	1
104	117	1
104	118	1
104	119	1
100	123	1
104	120	1
100	124	1
104	121	1
102	123	1
100	125	1
112	114	1
104	122	1
102	124	1
113	114	1
112	115	1
102	125	1
113	115	1
112	116	1
111	117	1
113	116	1
111	118	1
113	117	1
111	119	1
113	118	1
111	120	1
113	119	1
112	120	1
111	121	1
112	121	1
111	122	1
107	126	1
112	122	1
107	127	1
107	128	1
107	129	1
107	130	1
107	131	1
107	132	1
107	133	1
107	134	1
124	126	1
125	126	1
124	127	1
125	127	1
124	128	1
123	129	1
125	128	1
123	130	1
125	129	1

123	131	1
125	130	1
123	132	1
125	131	1
124	132	1
123	133	1
124	133	1
123	134	1
124	134	1

;
; GENERATED BY LigParGen Server
; Jorgensen Lab @ Yale University
;
[moleculetype]
; Name nrexcl
MAC 3
[atoms]

; nr	type	resnr	residue	atom	cgnr	charge	mass
1	opls_800	1	MAC	H00	1	0.0768	1.0080
2	opls_801	1	MAC	C01	1	0.0138	12.0110
3	opls_802	1	MAC	H02	1	0.0768	1.0080
4	opls_803	1	MAC	O03	1	-0.3442	15.9990
5	opls_804	1	MAC	C04	1	0.0119	12.0110
6	opls_805	1	MAC	C05	1	0.0091	12.0110
7	opls_806	1	MAC	H06	1	0.0785	1.0080
8	opls_807	1	MAC	H07	1	0.0785	1.0080
9	opls_808	1	MAC	O08	1	-0.3345	15.9990
10	opls_809	1	MAC	H09	1	0.0867	1.0080
11	opls_810	1	MAC	H0A	1	0.0867	1.0080
12	opls_811	1	MAC	C0B	1	0.0011	12.0110
13	opls_812	1	MAC	C0C	1	0.014	12.0110
14	opls_813	1	MAC	H0D	1	0.0802	1.0080
15	opls_814	1	MAC	H0E	1	0.0802	1.0080
16	opls_815	1	MAC	O0F	1	-0.3282	15.9990
17	opls_816	1	MAC	H0G	1	0.0697	1.0080
18	opls_817	1	MAC	H0H	1	0.0697	1.0080
19	opls_818	1	MAC	C0I	1	0.0318	12.0110
20	opls_819	1	MAC	C0J	1	0.0025	12.0110
21	opls_820	1	MAC	H0K	1	0.0525	1.0080
22	opls_821	1	MAC	H0M	1	0.0525	1.0080
23	opls_822	1	MAC	O0N	1	-0.2963	15.9990
24	opls_823	1	MAC	H0O	1	0.0783	1.0080
25	opls_824	1	MAC	H0P	1	0.0783	1.0080
26	opls_825	1	MAC	C0Q	1	0.01	12.0110
27	opls_826	1	MAC	C0R	1	0.0024	12.0110
28	opls_827	1	MAC	H0S	1	0.0819	1.0080
29	opls_828	1	MAC	H0T	1	0.0819	1.0080
30	opls_829	1	MAC	O0U	1	-0.3443	15.9990
31	opls_830	1	MAC	H0V	1	0.093	1.0080
32	opls_831	1	MAC	H0W	1	0.093	1.0080
33	opls_832	1	MAC	C0X	2	-0.056	12.0110
34	opls_833	1	MAC	C0Y	2	0.0058	12.0110
35	opls_834	1	MAC	H0Z	2	0.1162	1.0080
36	opls_835	1	MAC	H10	2	0.1162	1.0080
37	opls_836	1	MAC	O11	2	-0.395	15.9990
38	opls_837	1	MAC	H12	2	0.0853	1.0080
39	opls_838	1	MAC	H13	2	0.0853	1.0080
40	opls_839	1	MAC	C14	2	0.012	12.0110
41	opls_840	1	MAC	C15	2	-0.0322	12.0110
42	opls_841	1	MAC	H16	2	0.0751	1.0080

43	opls_842	1	MAC	H17	2	0.0751	1.0080
44	opls_843	1	MAC	O18	2	-0.3316	15.9990
45	opls_844	1	MAC	H19	2	0.119	1.0080
46	opls_845	1	MAC	H1A	2	0.119	1.0080
47	opls_846	1	MAC	C1B	2	0.0097	12.0110
48	opls_847	1	MAC	C1C	2	0.0054	12.0110
49	opls_848	1	MAC	H1D	2	0.0717	1.0080
50	opls_849	1	MAC	H1E	2	0.0717	1.0080
51	opls_850	1	MAC	O1F	2	-0.3299	15.9990
52	opls_851	1	MAC	H1G	2	0.0821	1.0080
53	opls_852	1	MAC	H1H	2	0.0821	1.0080
54	opls_853	1	MAC	C1I	2	0.0218	12.0110
55	opls_854	1	MAC	H1J	2	0.0734	1.0080
56	opls_855	1	MAC	H1K	2	0.0734	1.0080

[bonds]

2	1	1	0.1090	284512.000
3	2	1	0.1090	284512.000
4	2	1	0.1410	267776.000
5	2	1	0.1529	224262.400
6	4	1	0.1410	267776.000
7	5	1	0.1090	284512.000
8	5	1	0.1090	284512.000
9	5	1	0.1410	267776.000
10	6	1	0.1090	284512.000
11	6	1	0.1090	284512.000
12	6	1	0.1529	224262.400
13	9	1	0.1410	267776.000
14	12	1	0.1090	284512.000
15	12	1	0.1090	284512.000
16	12	1	0.1410	267776.000
17	13	1	0.1090	284512.000
18	13	1	0.1090	284512.000
19	13	1	0.1529	224262.400
20	16	1	0.1410	267776.000
21	19	1	0.1090	284512.000
22	19	1	0.1090	284512.000
23	19	1	0.1410	267776.000
24	20	1	0.1090	284512.000
25	20	1	0.1090	284512.000
26	20	1	0.1529	224262.400
27	23	1	0.1410	267776.000
28	26	1	0.1090	284512.000
29	26	1	0.1090	284512.000
30	26	1	0.1410	267776.000
31	27	1	0.1090	284512.000
32	27	1	0.1090	284512.000
33	27	1	0.1529	224262.400
34	30	1	0.1410	267776.000
35	33	1	0.1090	284512.000
36	33	1	0.1090	284512.000
37	33	1	0.1410	267776.000
38	34	1	0.1090	284512.000
39	34	1	0.1090	284512.000
40	34	1	0.1529	224262.400
41	37	1	0.1410	267776.000
42	40	1	0.1090	284512.000
43	40	1	0.1090	284512.000
44	40	1	0.1410	267776.000
45	41	1	0.1090	284512.000
46	41	1	0.1090	284512.000
47	41	1	0.1529	224262.400
48	44	1	0.1410	267776.000
49	47	1	0.1090	284512.000
50	47	1	0.1090	284512.000
51	47	1	0.1410	267776.000

52	48	1	0.1090	284512.000
53	48	1	0.1090	284512.000
54	48	1	0.1529	224262.400
55	54	1	0.1090	284512.000
56	54	1	0.1090	284512.000
54	51	1	0.1410	267776.000

[angles]

; ai	aj	ak	funct	c0	c1	c2	c3
1	2	3	1	107.800	276.144		
1	2	4	1	109.500	292.880		
1	2	5	1	110.700	313.800		
2	4	6	1	109.500	502.080		
2	5	7	1	110.700	313.800		
2	5	8	1	110.700	313.800		
2	5	9	1	109.500	418.400		
4	6	10	1	109.500	292.880		
4	6	11	1	109.500	292.880		
4	6	12	1	109.500	418.400		
5	9	13	1	109.500	502.080		
6	12	14	1	110.700	313.800		
6	12	15	1	110.700	313.800		
6	12	16	1	109.500	418.400		
9	13	17	1	109.500	292.880		
9	13	18	1	109.500	292.880		
9	13	19	1	109.500	418.400		
12	16	20	1	109.500	502.080		
13	19	21	1	110.700	313.800		
13	19	22	1	110.700	313.800		
13	19	23	1	109.500	418.400		
16	20	24	1	109.500	292.880		
16	20	25	1	109.500	292.880		
16	20	26	1	109.500	418.400		
19	23	27	1	109.500	502.080		
20	26	28	1	110.700	313.800		
20	26	29	1	110.700	313.800		
20	26	30	1	109.500	418.400		
23	27	31	1	109.500	292.880		
23	27	32	1	109.500	292.880		
23	27	33	1	109.500	418.400		
26	30	34	1	109.500	502.080		
27	33	35	1	110.700	313.800		
27	33	36	1	110.700	313.800		
27	33	37	1	109.500	418.400		
30	34	38	1	109.500	292.880		
30	34	39	1	109.500	292.880		
30	34	40	1	109.500	418.400		
33	37	41	1	109.500	502.080		
34	40	42	1	110.700	313.800		
34	40	43	1	110.700	313.800		
34	40	44	1	109.500	418.400		
37	41	45	1	109.500	292.880		
37	41	46	1	109.500	292.880		
37	41	47	1	109.500	418.400		
40	44	48	1	109.500	502.080		
41	47	49	1	110.700	313.800		
41	47	50	1	110.700	313.800		
41	47	51	1	109.500	418.400		
44	48	52	1	109.500	292.880		
44	48	53	1	109.500	292.880		
44	48	54	1	109.500	418.400		
48	54	55	1	110.700	313.800		
48	54	56	1	110.700	313.800		
29	26	30	1	109.500	292.880		
7	5	9	1	109.500	292.880		

17	13	19	1	110.700	313.800
25	20	26	1	110.700	313.800
38	34	40	1	110.700	313.800
47	51	54	1	109.500	502.080
28	26	29	1	107.800	276.144
48	54	51	1	109.500	418.400
49	47	51	1	109.500	292.880
15	12	16	1	109.500	292.880
11	6	12	1	110.700	313.800
53	48	54	1	110.700	313.800
22	19	23	1	109.500	292.880
10	6	12	1	110.700	313.800
51	54	55	1	109.500	292.880
4	2	5	1	109.500	418.400
8	5	9	1	109.500	292.880
52	48	54	1	110.700	313.800
7	5	8	1	107.800	276.144
24	20	26	1	110.700	313.800
18	13	19	1	110.700	313.800
14	12	16	1	109.500	292.880
42	40	43	1	107.800	276.144
46	41	47	1	110.700	313.800
10	6	11	1	107.800	276.144
52	48	53	1	107.800	276.144
35	33	36	1	107.800	276.144
38	34	39	1	107.800	276.144
31	27	32	1	107.800	276.144
51	54	56	1	109.500	292.880
42	40	44	1	109.500	292.880
50	47	51	1	109.500	292.880
31	27	33	1	110.700	313.800
14	12	15	1	107.800	276.144
32	27	33	1	110.700	313.800
45	41	46	1	107.800	276.144
43	40	44	1	109.500	292.880
3	2	5	1	110.700	313.800
35	33	37	1	109.500	292.880
39	34	40	1	110.700	313.800
17	13	18	1	107.800	276.144
55	54	56	1	107.800	276.144
28	26	30	1	109.500	292.880
24	20	25	1	107.800	276.144
21	19	23	1	109.500	292.880
36	33	37	1	109.500	292.880
3	2	4	1	109.500	292.880
21	19	22	1	107.800	276.144
49	47	50	1	107.800	276.144
45	41	47	1	110.700	313.800

[dihedrals]

; IMPROPER DIHEDRAL ANGLES

; ai	aj	ak	al	funct	c0	c1	c2	c3	c4	c5

[dihedrals]

; PROPER DIHEDRAL ANGLES

; ai	aj	ak	al	funct	c0	c1	c2	c3	c4	c5
19	13	9	5	3	1.715	2.845	1.046	-5.607	-0.000	0.000
40	34	30	26	3	1.715	2.845	1.046	-5.607	-0.000	0.000
54	48	44	40	3	1.715	2.845	1.046	-5.607	-0.000	0.000
33	27	23	19	3	1.715	2.845	1.046	-5.607	-0.000	0.000
12	6	4	2	3	1.715	2.845	1.046	-5.607	-0.000	0.000
47	41	37	33	3	1.715	2.845	1.046	-5.607	-0.000	0.000
26	20	16	12	3	1.715	2.845	1.046	-5.607	-0.000	0.000
48	54	51	47	3	1.715	2.845	1.046	-5.607	-0.000	0.000
20	16	12	6	3	1.715	2.845	1.046	-5.607	-0.000	0.000

41	37	33	27	3	1.715	2.845	1.046	-5.607	-0.000	0.000
6	4	2	5	3	1.715	2.845	1.046	-5.607	-0.000	0.000
48	44	40	34	3	1.715	2.845	1.046	-5.607	-0.000	0.000
13	9	5	2	3	1.715	2.845	1.046	-5.607	-0.000	0.000
27	23	19	13	3	1.715	2.845	1.046	-5.607	-0.000	0.000
34	30	26	20	3	1.715	2.845	1.046	-5.607	-0.000	0.000
54	51	47	41	3	1.715	2.845	1.046	-5.607	-0.000	0.000
34	30	26	28	3	1.590	4.770	0.000	-6.360	-0.000	0.000
48	44	40	42	3	1.590	4.770	0.000	-6.360	-0.000	0.000
13	9	5	7	3	1.590	4.770	0.000	-6.360	-0.000	0.000
48	44	40	43	3	1.590	4.770	0.000	-6.360	-0.000	0.000
13	9	5	8	3	1.590	4.770	0.000	-6.360	-0.000	0.000
27	23	19	22	3	1.590	4.770	0.000	-6.360	-0.000	0.000
20	16	12	14	3	1.590	4.770	0.000	-6.360	-0.000	0.000
54	51	47	49	3	1.590	4.770	0.000	-6.360	-0.000	0.000
6	4	2	3	3	1.590	4.770	0.000	-6.360	-0.000	0.000
41	37	33	36	3	1.590	4.770	0.000	-6.360	-0.000	0.000
34	30	26	29	3	1.590	4.770	0.000	-6.360	-0.000	0.000
41	37	33	35	3	1.590	4.770	0.000	-6.360	-0.000	0.000
54	51	47	50	3	1.590	4.770	0.000	-6.360	-0.000	0.000
20	16	12	15	3	1.590	4.770	0.000	-6.360	-0.000	0.000
6	4	2	1	3	1.590	4.770	0.000	-6.360	-0.000	0.000
27	23	19	21	3	1.590	4.770	0.000	-6.360	-0.000	0.000
35	33	27	31	3	0.628	1.883	0.000	-2.510	-0.000	0.000
14	12	6	10	3	0.628	1.883	0.000	-2.510	-0.000	0.000
42	40	34	38	3	0.628	1.883	0.000	-2.510	-0.000	0.000
50	47	41	46	3	0.628	1.883	0.000	-2.510	-0.000	0.000
49	47	41	45	3	0.628	1.883	0.000	-2.510	-0.000	0.000
21	19	13	17	3	0.628	1.883	0.000	-2.510	-0.000	0.000
14	12	6	11	3	0.628	1.883	0.000	-2.510	-0.000	0.000
28	26	20	24	3	0.628	1.883	0.000	-2.510	-0.000	0.000
49	47	41	46	3	0.628	1.883	0.000	-2.510	-0.000	0.000
56	54	48	53	3	0.628	1.883	0.000	-2.510	-0.000	0.000
42	40	34	39	3	0.628	1.883	0.000	-2.510	-0.000	0.000
8	5	2	3	3	0.628	1.883	0.000	-2.510	-0.000	0.000
43	40	34	38	3	0.628	1.883	0.000	-2.510	-0.000	0.000
55	54	48	52	3	0.628	1.883	0.000	-2.510	-0.000	0.000
43	40	34	39	3	0.628	1.883	0.000	-2.510	-0.000	0.000
36	33	27	32	3	0.628	1.883	0.000	-2.510	-0.000	0.000
7	5	2	3	3	0.628	1.883	0.000	-2.510	-0.000	0.000
29	26	20	24	3	0.628	1.883	0.000	-2.510	-0.000	0.000
7	5	2	1	3	0.628	1.883	0.000	-2.510	-0.000	0.000
28	26	20	25	3	0.628	1.883	0.000	-2.510	-0.000	0.000
50	47	41	45	3	0.628	1.883	0.000	-2.510	-0.000	0.000
22	19	13	17	3	0.628	1.883	0.000	-2.510	-0.000	0.000
21	19	13	18	3	0.628	1.883	0.000	-2.510	-0.000	0.000
29	26	20	25	3	0.628	1.883	0.000	-2.510	-0.000	0.000
22	19	13	18	3	0.628	1.883	0.000	-2.510	-0.000	0.000
15	12	6	10	3	0.628	1.883	0.000	-2.510	-0.000	0.000
8	5	2	1	3	0.628	1.883	0.000	-2.510	-0.000	0.000
56	54	48	52	3	0.628	1.883	0.000	-2.510	-0.000	0.000
55	54	48	53	3	0.628	1.883	0.000	-2.510	-0.000	0.000
35	33	27	32	3	0.628	1.883	0.000	-2.510	-0.000	0.000
15	12	6	11	3	0.628	1.883	0.000	-2.510	-0.000	0.000
36	33	27	31	3	0.628	1.883	0.000	-2.510	-0.000	0.000
50	47	41	37	3	0.979	2.937	0.000	-3.916	-0.000	0.000
14	12	6	4	3	0.979	2.937	0.000	-3.916	-0.000	0.000
15	12	6	4	3	0.979	2.937	0.000	-3.916	-0.000	0.000
29	26	20	16	3	0.979	2.937	0.000	-3.916	-0.000	0.000
8	5	2	4	3	0.979	2.937	0.000	-3.916	-0.000	0.000
49	47	41	37	3	0.979	2.937	0.000	-3.916	-0.000	0.000
56	54	48	44	3	0.979	2.937	0.000	-3.916	-0.000	0.000
53	48	54	51	3	0.979	2.937	0.000	-3.916	-0.000	0.000
35	33	27	23	3	0.979	2.937	0.000	-3.916	-0.000	0.000
22	19	13	9	3	0.979	2.937	0.000	-3.916	-0.000	0.000

42	40	34	30	3	0.979	2.937	0.000	-3.916	-0.000	0.000
55	54	48	44	3	0.979	2.937	0.000	-3.916	-0.000	0.000
36	33	27	23	3	0.979	2.937	0.000	-3.916	-0.000	0.000
43	40	34	30	3	0.979	2.937	0.000	-3.916	-0.000	0.000
52	48	54	51	3	0.979	2.937	0.000	-3.916	-0.000	0.000
21	19	13	9	3	0.979	2.937	0.000	-3.916	-0.000	0.000
28	26	20	16	3	0.979	2.937	0.000	-3.916	-0.000	0.000
7	5	2	4	3	0.979	2.937	0.000	-3.916	-0.000	0.000
56	54	51	47	3	1.590	4.770	0.000	-6.360	-0.000	0.000
53	48	44	40	3	1.590	4.770	0.000	-6.360	-0.000	0.000
39	34	30	26	3	1.590	4.770	0.000	-6.360	-0.000	0.000
45	41	37	33	3	1.590	4.770	0.000	-6.360	-0.000	0.000
18	13	9	5	3	1.590	4.770	0.000	-6.360	-0.000	0.000
11	6	4	2	3	1.590	4.770	0.000	-6.360	-0.000	0.000
24	20	16	12	3	1.590	4.770	0.000	-6.360	-0.000	0.000
32	27	23	19	3	1.590	4.770	0.000	-6.360	-0.000	0.000
46	41	37	33	3	1.590	4.770	0.000	-6.360	-0.000	0.000
38	34	30	26	3	1.590	4.770	0.000	-6.360	-0.000	0.000
52	48	44	40	3	1.590	4.770	0.000	-6.360	-0.000	0.000
17	13	9	5	3	1.590	4.770	0.000	-6.360	-0.000	0.000
10	6	4	2	3	1.590	4.770	0.000	-6.360	-0.000	0.000
25	20	16	12	3	1.590	4.770	0.000	-6.360	-0.000	0.000
31	27	23	19	3	1.590	4.770	0.000	-6.360	-0.000	0.000
55	54	51	47	3	1.590	4.770	0.000	-6.360	-0.000	0.000
37	33	27	32	3	0.979	2.937	0.000	-3.916	-0.000	0.000
9	5	2	3	3	0.979	2.937	0.000	-3.916	-0.000	0.000
30	26	20	24	3	0.979	2.937	0.000	-3.916	-0.000	0.000
37	33	27	31	3	0.979	2.937	0.000	-3.916	-0.000	0.000
23	19	13	17	3	0.979	2.937	0.000	-3.916	-0.000	0.000
16	12	6	10	3	0.979	2.937	0.000	-3.916	-0.000	0.000
23	19	13	18	3	0.979	2.937	0.000	-3.916	-0.000	0.000
30	26	20	25	3	0.979	2.937	0.000	-3.916	-0.000	0.000
51	47	41	45	3	0.979	2.937	0.000	-3.916	-0.000	0.000
44	40	34	39	3	0.979	2.937	0.000	-3.916	-0.000	0.000
44	40	34	38	3	0.979	2.937	0.000	-3.916	-0.000	0.000
16	12	6	11	3	0.979	2.937	0.000	-3.916	-0.000	0.000
9	5	2	1	3	0.979	2.937	0.000	-3.916	-0.000	0.000
51	47	41	46	3	0.979	2.937	0.000	-3.916	-0.000	0.000
44	40	34	30	3	-1.151	1.151	0.000	-0.000	-0.000	0.000
16	12	6	4	3	-1.151	1.151	0.000	-0.000	-0.000	0.000
23	19	13	9	3	-1.151	1.151	0.000	-0.000	-0.000	0.000
9	5	2	4	3	-1.151	1.151	0.000	-0.000	-0.000	0.000
51	54	48	44	3	-1.151	1.151	0.000	-0.000	-0.000	0.000
37	33	27	23	3	-1.151	1.151	0.000	-0.000	-0.000	0.000
30	26	20	16	3	-1.151	1.151	0.000	-0.000	-0.000	0.000
51	47	41	37	3	-1.151	1.151	0.000	-0.000	-0.000	0.000

[pairs]

1	6	1
1	7	1
3	6	1
1	8	1
3	7	1
1	9	1
5	6	1
4	7	1
3	8	1
4	8	1
3	9	1
2	10	1
4	9	1
2	11	1
2	12	1
2	13	1
4	14	1

4	15	1
7	13	1
4	16	1
8	13	1
5	17	1
5	18	1
10	14	1
5	19	1
11	14	1
10	15	1
11	15	1
10	16	1
6	20	1
11	16	1
9	21	1
9	22	1
9	23	1
14	20	1
15	20	1
12	24	1
12	25	1
17	21	1
12	26	1
18	21	1
17	22	1
18	22	1
17	23	1
13	27	1
18	23	1
16	28	1
16	29	1
16	30	1
21	27	1
22	27	1
19	31	1
19	32	1
24	28	1
19	33	1
25	28	1
24	29	1
25	29	1
24	30	1
20	34	1
25	30	1
23	35	1
23	36	1
23	37	1
28	34	1
29	34	1
26	38	1
26	39	1
31	35	1
26	40	1
32	35	1
31	36	1
32	36	1
31	37	1
27	41	1
32	37	1
30	42	1
30	43	1
30	44	1
35	41	1
36	41	1
33	45	1

33	46	1
38	42	1
33	47	1
39	42	1
38	43	1
39	43	1
38	44	1
34	48	1
39	44	1
37	49	1
37	50	1
37	51	1
42	48	1
43	48	1
40	52	1
40	53	1
45	49	1
40	54	1
47	48	1
46	49	1
45	50	1
44	51	1
41	54	1
46	50	1
45	51	1
46	51	1
44	55	1
44	56	1
47	55	1
51	52	1
49	54	1
47	56	1
51	53	1
50	54	1
52	55	1
53	55	1
52	56	1
53	56	1

Optimized geometry - at C-PCM(DMF)/B3LYP(D3)/cc-pVTZ level of theory - of the investigated Stop-[Bzi(24C8)-Bipy($PtCl_2$)-Bzi]-Stop complex:

Atom list:

1(Pt) --> Charge: 18.000000 x,y,z(Bohr): 1.835869 -2.663940 -2.607729
2(Cl) --> Charge: 17.000000 x,y,z(Bohr): -1.116476 -5.135660 -4.788648
3(Cl) --> Charge: 17.000000 x,y,z(Bohr): 5.140744 -5.195856 -4.228074
4(N) --> Charge: 7.000000 x,y,z(Bohr): 4.191206 -0.076553 -0.986563
5(N) --> Charge: 7.000000 x,y,z(Bohr): -0.754501 -0.314666 -0.988502
6(N) --> Charge: 7.000000 x,y,z(Bohr): -8.493980 -1.346515 2.001277
7(N) --> Charge: 7.000000 x,y,z(Bohr): -9.396421 1.952854 -0.501372
8(H) --> Charge: 1.000000 x,y,z(Bohr): -9.042604 3.530793 -1.565788
9(N) --> Charge: 7.000000 x,y,z(Bohr): 12.308226 -0.510466 -0.890954
10(H) --> Charge: 1.000000 x,y,z(Bohr): 11.592715 -2.020746 -1.823101
11(N) --> Charge: 7.000000 x,y,z(Bohr): 12.512095 3.287818 0.978207
12(C) --> Charge: 6.000000 x,y,z(Bohr): 3.001456 2.040077 -0.107504
13(C) --> Charge: 6.000000 x,y,z(Bohr): 4.416726 4.069017 0.833295
14(H) --> Charge: 1.000000 x,y,z(Bohr): 3.466167 5.752668 1.534311
15(C) --> Charge: 6.000000 x,y,z(Bohr): 7.033320 3.930968 0.881038
16(H) --> Charge: 1.000000 x,y,z(Bohr): 8.166925 5.490628 1.602422
17(C) --> Charge: 6.000000 x,y,z(Bohr): 8.241424 1.735419 -0.002987
18(C) --> Charge: 6.000000 x,y,z(Bohr): 6.725270 -0.227582 -0.912643
19(H) --> Charge: 1.000000 x,y,z(Bohr): 7.496000 -1.990466 -1.638867
20(C) --> Charge: 6.000000 x,y,z(Bohr): 10.996733 1.534876 0.036260

21(C) --> Charge: 6.000000 x,y,z(Bohr): 14.846866 -0.040021 -0.527667
22(C) --> Charge: 6.000000 x,y,z(Bohr): 14.939745 2.375912 0.639486
23(C) --> Charge: 6.000000 x,y,z(Bohr): 17.295384 3.493284 1.268885
24(C) --> Charge: 6.000000 x,y,z(Bohr): 19.438570 2.062006 0.650025
25(H) --> Charge: 1.000000 x,y,z(Bohr): 21.305945 2.840191 1.051332
26(C) --> Charge: 6.000000 x,y,z(Bohr): 19.300484 -0.327733 -0.511863
27(H) --> Charge: 1.000000 x,y,z(Bohr): 21.055151 -1.334022 -0.918108
28(C) --> Charge: 6.000000 x,y,z(Bohr): 17.000072 -1.479698 -1.137638
29(C) --> Charge: 6.000000 x,y,z(Bohr): 16.832627 -3.988886 -2.368215
30(C) --> Charge: 6.000000 x,y,z(Bohr): 18.481604 -4.667051 -4.333182
31(H) --> Charge: 1.000000 x,y,z(Bohr): 19.893307 -3.311591 -4.989013
32(C) --> Charge: 6.000000 x,y,z(Bohr): 18.316485 -7.029269 -5.505191
33(H) --> Charge: 1.000000 x,y,z(Bohr): 19.636039 -7.443670 -7.030439
34(C) --> Charge: 6.000000 x,y,z(Bohr): 16.499038 -8.820864 -4.777053
35(C) --> Charge: 6.000000 x,y,z(Bohr): 14.861962 -8.135911 -2.794171
36(H) --> Charge: 1.000000 x,y,z(Bohr): 13.429381 -9.465669 -2.134720
37(C) --> Charge: 6.000000 x,y,z(Bohr): 15.020007 -5.787506 -1.612327
38(H) --> Charge: 1.000000 x,y,z(Bohr): 13.749430 -5.372831 -0.038648
39(C) --> Charge: 6.000000 x,y,z(Bohr): 16.241317 -11.426965 -6.037726
40(C) --> Charge: 6.000000 x,y,z(Bohr): 13.566756 -11.678307 -7.186444
41(H) --> Charge: 1.000000 x,y,z(Bohr): 13.240011 -10.212041 -8.628928
42(H) --> Charge: 1.000000 x,y,z(Bohr): 12.082535 -11.497077 -5.740011
43(H) --> Charge: 1.000000 x,y,z(Bohr): 13.345089 -13.541276 -8.092549
44(C) --> Charge: 6.000000 x,y,z(Bohr): 18.188492 -11.823344 -8.160613
45(H) --> Charge: 1.000000 x,y,z(Bohr): 17.928582 -13.708659 -9.002760
46(H) --> Charge: 1.000000 x,y,z(Bohr): 20.141409 -11.716005 -7.448287
47(H) --> Charge: 1.000000 x,y,z(Bohr): 17.970625 -10.419035 -9.681486
48(C) --> Charge: 6.000000 x,y,z(Bohr): 16.635424 -13.512886 -4.030571
49(H) --> Charge: 1.000000 x,y,z(Bohr): 15.235566 -13.386216 -2.496960
50(H) --> Charge: 1.000000 x,y,z(Bohr): 18.532634 -13.375165 -3.182591
51(H) --> Charge: 1.000000 x,y,z(Bohr): 16.449734 -15.395368 -4.903846
52(C) --> Charge: 6.000000 x,y,z(Bohr): 17.507281 6.003853 2.490717
53(C) --> Charge: 6.000000 x,y,z(Bohr): 19.415893 6.496861 4.281536
54(H) --> Charge: 1.000000 x,y,z(Bohr): 20.708219 4.987700 4.839980
55(C) --> Charge: 6.000000 x,y,z(Bohr): 19.656648 8.868252 5.399769
56(H) --> Charge: 1.000000 x,y,z(Bohr): 21.158000 9.147282 6.787414
57(C) --> Charge: 6.000000 x,y,z(Bohr): 18.016012 10.876289 4.797939
58(C) --> Charge: 6.000000 x,y,z(Bohr): 16.114424 10.373661 3.017217
59(H) --> Charge: 1.000000 x,y,z(Bohr): 14.787841 11.848870 2.465073
60(C) --> Charge: 6.000000 x,y,z(Bohr): 15.857071 7.997269 1.891416
61(H) --> Charge: 1.000000 x,y,z(Bohr): 14.356151 7.689216 0.516018
62(C) --> Charge: 6.000000 x,y,z(Bohr): 18.351078 13.463480 6.079506
63(C) --> Charge: 6.000000 x,y,z(Bohr): 21.015990 14.499838 5.480383
64(H) --> Charge: 1.000000 x,y,z(Bohr): 22.512803 13.229223 6.167978
65(H) --> Charge: 1.000000 x,y,z(Bohr): 21.289930 16.353562 6.391579
66(H) --> Charge: 1.000000 x,y,z(Bohr): 21.271460 14.741982 3.427607
67(C) --> Charge: 6.000000 x,y,z(Bohr): 18.059809 13.153258 8.969932
68(H) --> Charge: 1.000000 x,y,z(Bohr): 16.170482 12.418902 9.448483
69(H) --> Charge: 1.000000 x,y,z(Bohr): 18.301215 14.991907 9.920111
70(H) --> Charge: 1.000000 x,y,z(Bohr): 19.474558 11.844833 9.753766
71(C) --> Charge: 6.000000 x,y,z(Bohr): 16.390741 15.409009 5.169422
72(H) --> Charge: 1.000000 x,y,z(Bohr): 16.534793 15.750905 3.121220
73(H) --> Charge: 1.000000 x,y,z(Bohr): 16.708263 17.225232 6.135030
74(H) --> Charge: 1.000000 x,y,z(Bohr): 14.445320 14.802273 5.593347
75(C) --> Charge: 6.000000 x,y,z(Bohr): 0.235769 1.920543 -0.131621
76(C) --> Charge: 6.000000 x,y,z(Bohr): -1.331785 3.842309 0.749102
77(H) --> Charge: 1.000000 x,y,z(Bohr): -0.531723 5.636558 1.358143
78(C) --> Charge: 6.000000 x,y,z(Bohr): -3.922898 3.432963 0.906287
79(H) --> Charge: 1.000000 x,y,z(Bohr): -5.200952 4.893984 1.585454
80(C) --> Charge: 6.000000 x,y,z(Bohr): -4.871197 1.058429 0.251683
81(C) --> Charge: 6.000000 x,y,z(Bohr): -3.237791 -0.780026 -0.715996
82(H) --> Charge: 1.000000 x,y,z(Bohr): -3.941015 -2.611046 -1.330619
83(C) --> Charge: 6.000000 x,y,z(Bohr): -7.568572 0.492117 0.594214
84(C) --> Charge: 6.000000 x,y,z(Bohr): -11.698873 0.970345 0.215595
85(C) --> Charge: 6.000000 x,y,z(Bohr): -11.092291 -1.075659 1.840494

86(C) --> Charge: 6.000000 x,y,z(Bohr): -13.015099 -2.519680 3.004778
87(C) --> Charge: 6.000000 x,y,z(Bohr): -15.504782 -1.825757 2.419808
88(H) --> Charge: 1.000000 x,y,z(Bohr): -17.060829 -2.876110 3.277456
89(C) --> Charge: 6.000000 x,y,z(Bohr): -16.074934 0.197748 0.784555
90(H) --> Charge: 1.000000 x,y,z(Bohr): -18.054187 0.626700 0.387359
91(C) --> Charge: 6.000000 x,y,z(Bohr): -14.197456 1.662646 -0.384385
92(C) --> Charge: 6.000000 x,y,z(Bohr): -14.758281 3.795726 -2.108588
93(C) --> Charge: 6.000000 x,y,z(Bohr): -16.690850 5.532635 -1.572426
94(H) --> Charge: 1.000000 x,y,z(Bohr): -17.814284 5.302184 0.143922
95(C) --> Charge: 6.000000 x,y,z(Bohr): -17.156524 7.597895 -3.154812
96(H) --> Charge: 1.000000 x,y,z(Bohr): -18.658393 8.902959 -2.624513
97(C) --> Charge: 6.000000 x,y,z(Bohr): -15.723714 8.015403 -5.348455
98(C) --> Charge: 6.000000 x,y,z(Bohr): -13.816820 6.241059 -5.894425
99(H) --> Charge: 1.000000 x,y,z(Bohr): -12.668192 6.455781 -7.594293
100(C) --> Charge: 6.000000 x,y,z(Bohr): -13.326811 4.191101 -4.318648
101(H) --> Charge: 1.000000 x,y,z(Bohr): -11.807686 2.889692 -4.821827
102(C) --> Charge: 6.000000 x,y,z(Bohr): -16.126265 10.295943 -7.103391
103(C) --> Charge: 6.000000 x,y,z(Bohr): -16.762006 9.353685 -9.794023
104(H) --> Charge: 1.000000 x,y,z(Bohr): -17.044172 10.971771 -11.075833
105(H) --> Charge: 1.000000 x,y,z(Bohr): -18.508435 8.219065 -9.787166
106(H) --> Charge: 1.000000 x,y,z(Bohr): -15.236951 8.180110 -10.584345
107(C) --> Charge: 6.000000 x,y,z(Bohr): -13.671677 11.879615 -7.184265
108(H) --> Charge: 1.000000 x,y,z(Bohr): -12.062218 10.776106 -7.905322
109(H) --> Charge: 1.000000 x,y,z(Bohr): -13.173023 12.569268 -5.283051
110(H) --> Charge: 1.000000 x,y,z(Bohr): -13.917528 13.531033 -8.430659
111(C) --> Charge: 6.000000 x,y,z(Bohr): -18.291560 12.014051 -6.201434
112(H) --> Charge: 1.000000 x,y,z(Bohr): -17.918263 12.798603 -4.309989
113(H) --> Charge: 1.000000 x,y,z(Bohr): -20.107646 10.998731 -6.139343
114(H) --> Charge: 1.000000 x,y,z(Bohr): -18.508173 13.613941 -7.514772
115(C) --> Charge: 6.000000 x,y,z(Bohr): -12.360274 -4.654849 4.690519
116(C) --> Charge: 6.000000 x,y,z(Bohr): -13.601633 -6.990482 4.519496
117(H) --> Charge: 1.000000 x,y,z(Bohr): -15.134170 -7.229313 3.156574
118(C) --> Charge: 6.000000 x,y,z(Bohr): -12.849833 -9.052911 5.996228
119(H) --> Charge: 1.000000 x,y,z(Bohr): -13.854876 -10.833919 5.753803
120(C) --> Charge: 6.000000 x,y,z(Bohr): -10.831482 -8.870246 7.707532
121(C) --> Charge: 6.000000 x,y,z(Bohr): -9.639363 -6.497844 7.917125
122(H) --> Charge: 1.000000 x,y,z(Bohr): -8.071542 -6.246113 9.235041
123(C) --> Charge: 6.000000 x,y,z(Bohr): -10.376344 -4.440563 6.454145
124(H) --> Charge: 1.000000 x,y,z(Bohr): -9.377526 -2.648918 6.638261
125(C) --> Charge: 6.000000 x,y,z(Bohr): -9.866307 -11.117008 9.277532
126(C) --> Charge: 6.000000 x,y,z(Bohr): -11.431578 -13.527490 8.837664
127(C) --> Charge: 6.000000 x,y,z(Bohr): -9.987460 -10.453002 12.119137
128(H) --> Charge: 1.000000 x,y,z(Bohr): -8.822520 -8.791889 12.578081
129(H) --> Charge: 1.000000 x,y,z(Bohr): -9.290078 -12.049124 13.262545
130(C) --> Charge: 6.000000 x,y,z(Bohr): -7.096230 -11.671954 8.527478
131(H) --> Charge: 1.000000 x,y,z(Bohr): -6.352614 -13.273947 9.633180
132(H) --> Charge: 1.000000 x,y,z(Bohr): -5.860617 -10.033476 8.871032
133(H) --> Charge: 1.000000 x,y,z(Bohr): -6.963163 -12.160032 6.506858
134(H) --> Charge: 1.000000 x,y,z(Bohr): -11.945390 -10.045413 12.700742
135(H) --> Charge: 1.000000 x,y,z(Bohr): -10.674048 -15.076594 10.003243
136(H) --> Charge: 1.000000 x,y,z(Bohr): -13.427111 -13.257719 9.365359
137(H) --> Charge: 1.000000 x,y,z(Bohr): -11.360785 -14.143501 6.850790
138(O) --> Charge: 8.000000 x,y,z(Bohr): -5.873648 2.595030 6.209925
139(C) --> Charge: 6.000000 x,y,z(Bohr): -3.881324 1.812883 7.834053
140(H) --> Charge: 1.000000 x,y,z(Bohr): -4.572752 1.753399 9.811293
141(H) --> Charge: 1.000000 x,y,z(Bohr): -2.287468 3.170878 7.759510
142(C) --> Charge: 6.000000 x,y,z(Bohr): -2.930454 -0.804094 7.158047
143(H) --> Charge: 1.000000 x,y,z(Bohr): -1.867767 -1.530772 8.809992
144(H) --> Charge: 1.000000 x,y,z(Bohr): -4.552825 -2.076623 6.820077
145(O) --> Charge: 8.000000 x,y,z(Bohr): -1.330311 -0.709426 5.002445
146(C) --> Charge: 6.000000 x,y,z(Bohr): 0.337686 -2.802934 4.786063
147(H) --> Charge: 1.000000 x,y,z(Bohr): 1.418876 -2.474234 3.045161
148(H) --> Charge: 1.000000 x,y,z(Bohr): 1.687273 -2.845192 6.391831
149(C) --> Charge: 6.000000 x,y,z(Bohr): -0.942454 -5.378581 4.642746
150(H) --> Charge: 1.000000 x,y,z(Bohr): -1.786358 -5.900742 6.489331

151(H) --> Charge: 1.000000 x,y,z(Bohr):   0.530677 -6.808973   4.215399
152(O) --> Charge: 8.000000 x,y,z(Bohr): -2.839011 -5.323819   2.758391
153(C) --> Charge: 6.000000 x,y,z(Bohr): -4.050028 -7.697645   2.404059
154(H) --> Charge: 1.000000 x,y,z(Bohr): -2.868602 -8.936461   1.196408
155(H) --> Charge: 1.000000 x,y,z(Bohr): -4.321399 -8.660142   4.242606
156(C) --> Charge: 6.000000 x,y,z(Bohr): -6.629502 -7.309339   1.234699
157(H) --> Charge: 1.000000 x,y,z(Bohr): -7.620902 -9.158697   1.182455
158(H) --> Charge: 1.000000 x,y,z(Bohr): -7.730781 -5.997951   2.417003
159(O) --> Charge: 8.000000 x,y,z(Bohr): -6.361118 -6.319574 -1.244974
160(C) --> Charge: 6.000000 x,y,z(Bohr): -8.713411 -5.888940 -2.452453
161(H) --> Charge: 1.000000 x,y,z(Bohr): -9.933582 -4.657222 -1.292666
162(H) --> Charge: 1.000000 x,y,z(Bohr): -9.726290 -7.703639 -2.744095
163(C) --> Charge: 6.000000 x,y,z(Bohr): -8.255631 -4.689350 -5.004195
164(H) --> Charge: 1.000000 x,y,z(Bohr): -6.839915 -5.820174 -6.051763
165(H) --> Charge: 1.000000 x,y,z(Bohr): -10.044155 -4.711694 -6.097278
166(O) --> Charge: 8.000000 x,y,z(Bohr): -7.373886 -2.168962 -4.705614
167(C) --> Charge: 6.000000 x,y,z(Bohr): -6.712106 -1.071640 -7.059596
168(H) --> Charge: 1.000000 x,y,z(Bohr): -8.427722 -0.679907 -8.200190
169(H) --> Charge: 1.000000 x,y,z(Bohr): -5.502559 -2.387472 -8.148458
170(C) --> Charge: 6.000000 x,y,z(Bohr): -5.224408   1.336482 -6.686922
171(H) --> Charge: 1.000000 x,y,z(Bohr): -3.596899   0.968631 -5.428210
172(H) --> Charge: 1.000000 x,y,z(Bohr): -4.464599   1.925328 -8.548716
173(O) --> Charge: 8.000000 x,y,z(Bohr): -6.776301   3.288414 -5.677829
174(C) --> Charge: 6.000000 x,y,z(Bohr): -5.420644   5.593640 -5.430497
175(H) --> Charge: 1.000000 x,y,z(Bohr): -4.661654   6.209253 -7.283426
176(H) --> Charge: 1.000000 x,y,z(Bohr): -3.803353   5.344443 -4.125994
177(C) --> Charge: 6.000000 x,y,z(Bohr): -7.161894   7.623617 -4.430099
178(H) --> Charge: 1.000000 x,y,z(Bohr): -6.098225   9.417844 -4.287811
179(H) --> Charge: 1.000000 x,y,z(Bohr): -8.775402   7.918802 -5.722831
180(O) --> Charge: 8.000000 x,y,z(Bohr): -8.050428   6.866606 -2.007865
181(C) --> Charge: 6.000000 x,y,z(Bohr): -9.367267   8.799505 -0.671961
182(H) --> Charge: 1.000000 x,y,z(Bohr): -10.946167   9.540310 -1.824959
183(H) --> Charge: 1.000000 x,y,z(Bohr): -8.055586 10.372977 -0.248534
184(C) --> Charge: 6.000000 x,y,z(Bohr): -10.429170   7.708349   1.747337
185(H) --> Charge: 1.000000 x,y,z(Bohr): -11.502299   9.207348   2.742114
186(H) --> Charge: 1.000000 x,y,z(Bohr): -11.767563   6.171498   1.306139
187(O) --> Charge: 8.000000 x,y,z(Bohr): -8.422718   6.802306   3.287687
188(C) --> Charge: 6.000000 x,y,z(Bohr): -9.317315   5.496139   5.463809
189(H) --> Charge: 1.000000 x,y,z(Bohr): -10.377260   3.780073   4.905890
190(H) --> Charge: 1.000000 x,y,z(Bohr): -10.612671   6.734095   6.546527
191(C) --> Charge: 6.000000 x,y,z(Bohr): -7.125362   4.773941   7.154035
192(H) --> Charge: 1.000000 x,y,z(Bohr): -5.805657   6.394984   7.301006
193(H) --> Charge: 1.000000 x,y,z(Bohr): -7.868344   4.389059   9.075290

Note: Orbital   367 is HOMO, energy: -0.206445 a.u.   -5.617652 eV
Orbital   368 is LUMO, energy: -0.103263 a.u.   -2.809922 eV
HOMO-LUMO gap: 0.103182 a.u.   2.807730 eV   270.904866 kJ/mol

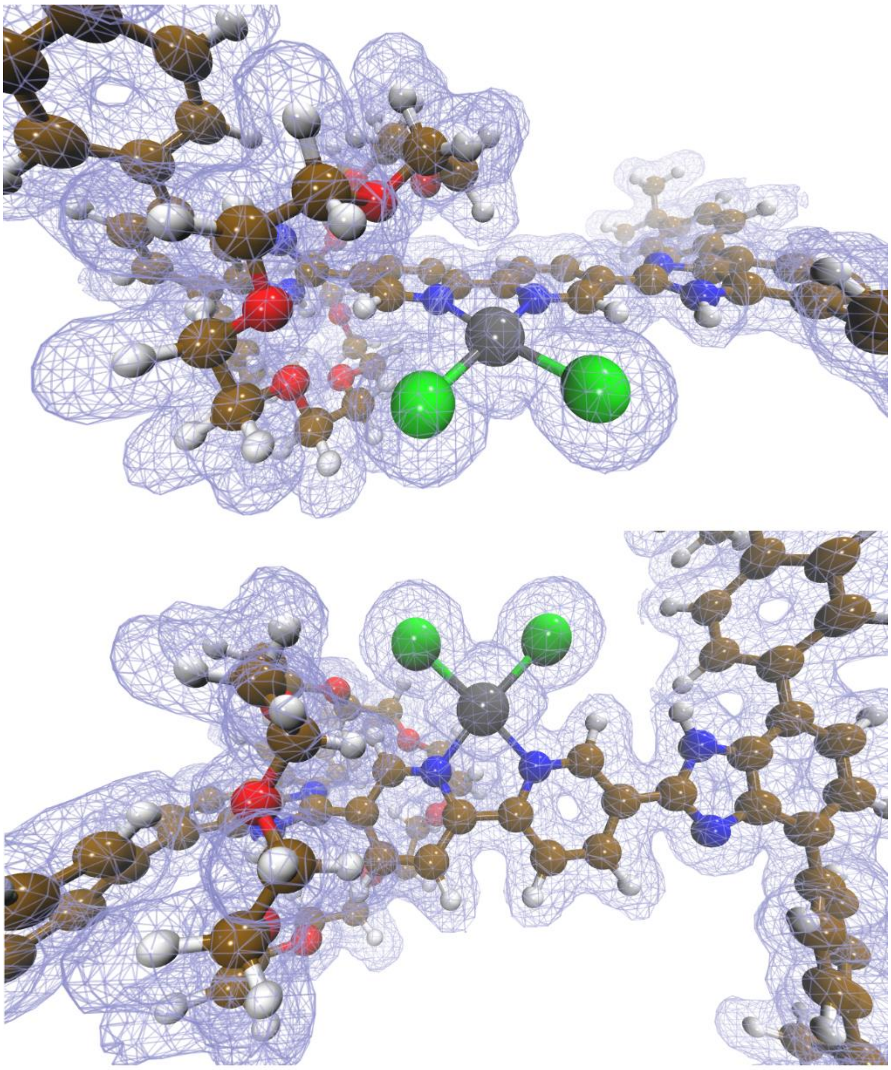

Figure S1. 3D Plot of the *H(0,ISO)* taking as reference the optimized geometry of the Stop-[Bzi(24C8)-Bipy($PtCl_2$)-Bzi]-Stop complex at C-PCM(DMF)/B3LYP(D3)/cc-pVTZ level of theory.
